# A frog peptide provides new strategies for the intervention against skin wound healing

**DOI:** 10.1186/s11658-023-00468-3

**Published:** 2023-07-28

**Authors:** Chao Li, Zhe Fu, Tao Jin, Yixiang Liu, Naixin Liu, Saige Yin, Zhuo Wang, Yubing Huang, Yinglei Wang, Yingxuan Zhang, Jiayi Li, Yutong Wu, Li He, Jing Tang, Ying Wang, Xinwang Yang

**Affiliations:** 1 Department of Anatomy and Histology & Embryology, Faculty of Basic Medical Science, Kunming Medical University, Kunming, 650500 Yunnan China; 2 Key Laboratory of Chemistry in Ethnic Medicinal Resources & Key Laboratory of Natural Products Synthetic Biology of Ethnic Medicinal Endophytes, State Ethnic Affairs Commission & Ministry of Education, School of Ethnic Medicine, Yunnan Minzu University, Kunming, 650504 Yunnan China; 3 Department of Biochemistry and Molecular Biology, Faculty of Basic Medical Science, Kunming Medical University, Kunming, 650500 Yunnan China; 4 Department of Dermatology, First Affiliated Hospital of Kunming Medical University, Kunming, 650032 Yunnan China; 5 Department of Orthopedics, 920th Hospital of Joint Logistics Support Force of PLA, Kunming, 650032 Yunnan China

**Keywords:** Pro-healing peptide, Amphibians, Skin wounds, TLR4/MAPK, Inflammation, miR-632/Wnt/β-catenin

## Abstract

**Background:**

Amphibian derived pro-healing peptides as molecular probes might provide a promising strategy for development of drug candidates and elucidation of cellular and molecular mechanisms of skin wound healing. A novel skin amphibian peptide, OA-RD17, was tested for modulation of cellular and molecular mechanisms associated with skin wound healing.

**Methods:**

Cell scratch, cell proliferation, trans-well, and colony formation assays were used to explore the pro-healing ability of peptide OA-RD17 and microRNA-632 (miR-632). Then, the therapeutic effects of OA-RD17 and miR-632 were assessed in mice, diabetic patient ex vivo skin wounds and SD rats. Moreover, hematoxylin and eosin (H&E), enzyme-linked immunosorbent assay (ELISA), immunohistochemistry, and immunofluorescence staining were performed to detect skin wound tissue regeneration, inflammatory factors expression, and macrophage polarization. Finally, RNA sequencing, molecular docking, co-localization, dual luciferase reporter, real-time quantitative reverse transcription PCR (RT-qPCR), and Western blotting were used to explore the mechanism of OA-RD17 and miR-632 on facilitating skin wound healing.

**Results:**

The non-toxic peptide (OA-RD17) promoted macrophage proliferation and migration by activating MAPK and suppressed inflammation by inhibiting NF-κB. In keratinocytes, OA-RD17 inhibited excessive inflammation, and activated MAPK via the Toll-like receptor 4 (TLR4) to promote proliferation and migration, as well as up-regulate the expression of miR-632, which targeted GSK3β to activate Wnt/β-catenin to boost proliferation and migration in a positive feedback manner. Notably, OA-RD17 promoted transition from the inflammatory to proliferative stage, accelerated epidermal and granulation regeneration, and exhibited therapeutic effects on mouse and diabetic patient ex vivo skin wounds. MiR-632 activated Wnt/β-catenin to promote full-thickness skin wound healing in rats.

**Conclusions:**

OA-RD17 exhibited promising therapeutic effects on mice (full-thickness, deep second-degree burns), and ex vivo skin wounds in diabetic patients by regulating macrophages proliferation, migration, and polarization (MAPK, NF-κB), and keratinocytes proliferation and migration (TLR4/MAPK/miR-632/Wnt/β-catenin molecular axis). Moreover, miR-632 also activated Wnt/β-catenin to promote full-thickness skin wound healing in rats. Notably, our results indicate that OA-RD17 and miR-632 are promising pro-healing drug candidates.

**Graphical Abstract:**

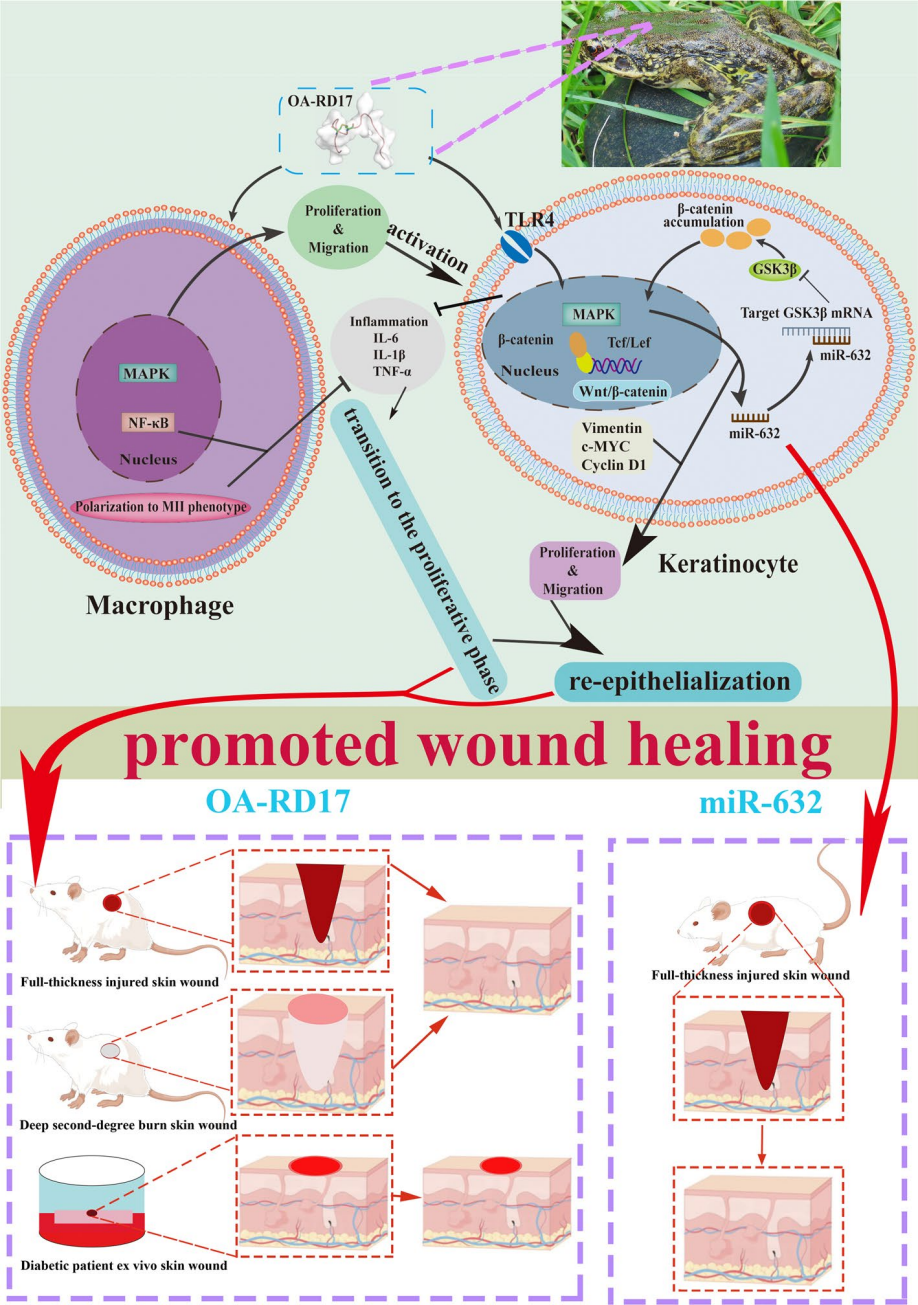

**Supplementary Information:**

The online version contains supplementary material available at 10.1186/s11658-023-00468-3.

## Background

As the largest organ of the body, skin not only forms the primary natural protective barrier, but also plays a vital role in various physiological processes [1]. Once damaged, the organism immediately initiates the complex process of wound healing, which is susceptible to the development of chronic non-healing wounds as a result of disease and external environmental influences [2, 3]. Chronic non-healing wounds represent a huge burden on global healthcare systems due to the lack of efficient and targeted treatment and associated high clinical costs [3, 4]. Despite enormous efforts to improve the rehabilitation of skin wounds, available therapies for chronic skin wound treatment remain a considerable challenge [3, 5–7].

Amphibian-derived peptides are considered as a veritable treasure trove for the development of peptide-based drugs [8–14]. Notably, amphibian-derived pro-healing peptides provide a promising intervention strategy for chronic wound healing [15]. For example, RL-QN15, OA-GP11 dimer, and OA-GL17d exhibit significant therapeutic effects in skin wound healing, even at low concentrations (nM scale) [16–18]. Therefore, exploring the expression of endogenous genes using highly specific exogenous pro-healing peptide as probe may help in the discovery of new therapeutic strategies and drug targets for skin wound repair. At present, however, research is still in the preliminary stage and extensive investigations are required.

Skin wound healing is divided into four precisely regulated processes: i.e., hemostasis, inflammation, proliferation, and tissue-remodeling [4, 19]. Regeneration of defective epidermis and formation of granulation tissue are the result of organism-regulated functional cell behaviors [20–22]. Macrophages play important regulatory roles in wound healing, especially the transition from the inflammatory to proliferative phase [23–25]. Keratinocytes, the main cellular component of the epidermis, play an important role in the proliferative phase of wound healing, which not only have immunoregulatory functions but also contribute to epidermal regeneration through their proliferation and migration [4, 26]. In chronic wounds, the persistent excessive inflammatory response, which severely impairs the proliferation, migration, and immunomodulatory functions of macrophages and keratinocytes, significantly impedes wound healing. Therefore, promoting the proliferation and migration of macrophages and keratinocytes, as well as inhibiting excessive inflammation in wounds are considered critical to wound repair.

TLR4 mainly expressed in keratinocytes, regulates inflammatory responses by mediating the NF-κB and MAPK signaling pathways [26]. TLR4-deficient mice significantly delayed wound healing, along with reduced inflammatory factor and increased risk of infection [27]. Moreover, the NF-κB and MAPK signaling pathways are tightly associated with immune response, cell proliferation and migration [17, 28]. Although ‘TLR4/MAPK’ regulates the expression of inflammatory factors, the regulation of keratinocyte proliferation and migration by the ‘TLR4/MAPK’ pathway, thus promoting re-epithelialization, remains unknown. Thus, probing the molecular mechanism of TLR4 regulating keratinocytes will provide new insights for skin wound healing.

MicroRNAs (miRNAs) are important for skin wound healing, and modulating the expression of miRNA in animal and cultured human tissues have shown great therapeutic effects on skin wounds, with potential as novel therapeutic targets and nucleic acid drugs [4, 29]. The expression levels of miRNAs are regulated by specific signaling pathways, and miRNAs usually exert biological functions by targeting specific genes [30]. For example, miR-632 can target GSK3β to regulate the Wnt/β-catenin signaling pathway [31]. Furthermore, the Wnt/β-catenin signaling pathway is closely related to cell proliferation, migration, and differentiation, and has an important regulatory effect in skin wound regeneration [32]. However, the effects of the Wnt/β-catenin signaling pathway in promoting keratinocyte proliferation and migration to accelerate skin wound healing remain controversial and require further study [33–35].

In the current study, we identified a novel peptide (OA-RD17) from skin tissue of *Odorrana andersonii*. OA-RD17 exhibited excellent therapeutic on skin wounds in mice and ex vivo skin wounds in diabetic patients, highlighting its important role in promoting skin wound regeneration. OA-RD17 facilitated the proliferation, migration, and polarization of macrophages, significantly inhibited excessive inflammatory reaction, and promoted the transition of wound repair from inflammatory phase to proliferative phase. Moreover, using OA-RD17 as a molecular probe, our study provides the first evidence that both the ‘TLR4/MAPK’ and ‘miR-632/Wnt/β-catenin’ signaling pathways are closely involved in the proliferation and migration of keratinocytes, thereby facilitating skin wound regeneration. Our study also indicates that OA-RD17 and miR-632 are promising skin wound regeneration drug candidates and suggests that amphibian-derived peptides may play crucial roles in providing novel strategies for the treatment of skin wounds.

## Materials and methods

### Screening of cDNA-encoded peptide OA-RD17 from *Odorrana andersonii* skin cDNA library

The cDNA sequence of the peptide OA-RD17 (RDYCTPEDCDYDFSFPI) was obtained from the *Odorrana andersonii* skin cDNA library previously constructed by our team [36]. Briefly, clones with inserts > 300 bp in the cDNA library were randomly selected and then subjected to DNA sequencing on an Applied Biosystems DNA sequencer (ABI 3730xl, Foster City, CA) to obtain the cDNA sequence of the peptides.

### Peptide synthesis and structure prediction

OA-RD17, FITC-labeled OA-RD17, and scrambled peptide (RCDYDEDYFCFPTPDSI) with a purity of > 95% were commercially synthesized by Wuhan Bioyeargene Biotechnology Co., Ltd. (Wuhan, China). Structure prediction of OA-RD17 was performed according to previous study [17].

### Hemolytic and acute activities of OA-RD17

The hemolytic activity of OA-RD17 (100 pM, 1 nM, 10 nM, 100 nM, and 1 μM) against Kunming mouse red blood cells was determined according to previous study [37]. To examine the acute toxicity of OA-RD17, Kunming mice were intraperitoneally injected with OA-RD17 (0.1 μg/kg, 1 μg/kg, 10 μg/kg, 100 μg/kg, 1 mg/kg) or phosphate-buffered saline (PBS, vehicle) to detect mouse death and body weight changes after 1 week.

### Primary mouse keratinocytes and macrophages isolation

Primary keratinocytes were isolated from the skin of neonatal Kunming mice according to previous study [38]. Briefly, the skin of newborn mice was removed and placed in a centrifuge tube containing 10 mL dispase II (Thermo Fisher Scientifi, China) for 12 h at 4 °C. The skin epidermis was isolated and keratinocytes from the epidermis were digested by pancreatin. Finally, the supernatant was collected and centrifuged (1000 *g*, 3 min) to obtain primary keratinocytes.

Primary macrophages from Kunming mice were extracted according to previous study [39]. Briefly, Kunming mice were sacrificed, sterilized, and then intraperitoneally injected with 3 mL high glucose DMEM (BI, Israel) containing antibodies (100 units/mL penicillin and 100 units/mL streptomycin). Next, mice intraperitoneal fluid was collected and then centrifuged (1000 *g*, 3 min) to obtain primary macrophages.

### Cell culture

The human immortalized keratinocyte cell line (HaCaT, KCB04045YJ) and mouse leukemic monocyte/macrophage cell line (RAW264.7, KCB200603YJ) were obtained from the cell bank of Kunming Institute of Zoology (Yunnan, China). The keratinocytes and primary keratinocytes were cultured in Dulbecco’s Modified Eagle Medium/nutrient mixture F12 (DMEM/F12; BI, Israel) supplemented with 10% (V/V) fetal bovine serum (FBS, Hy-Clone, USA) and antibiotics (100 units/mL penicillin and 100 units/mL streptomycin) at 37 °C in a humidified atmosphere of 5% CO_2_. The macrophages and primary macrophages were cultured in high glucose DMEM (BI, Israel) supplemented with inactivated 10% (V/V) FBS (HyClone, USA) and antibiotics (100 units/mL penicillin and 100 units/mL streptomycin) at 37 °C in a humidified atmosphere of 5% CO_2_.

### Cell scratch

Cell scratch assays were performed to investigate the effects of OA-RD17 (1 nM), miR-632 mimic (50 nM), and miR-632 inhibitor (100 nM) (riboFECT™ CP Transfection Kit, RiboBio, China) on keratinocyte scratch repair following previous study [16]. In addition, the underlying mechanisms of OA-RD17- and miR-632-induced keratinocyte scratch healing were determined using a specific TLR4 inhibitor (1 μM, HY-11109, MedChemExpress, USA), MAPK signaling pathway inhibitor (1 μM, HY-N1966, MedChemExpress, USA), or β-catenin inhibitor (10 μM, HY-120697, MedChemExpress, USA).

### Cell proliferation

The pro-proliferative effects of OA-RD17 (1 nM) on keratinocytes, macrophages, primary macrophages, and primary keratinocytes were determined according to previous study [16]. The effects of miR-632 mimic (50 nM), and miR-632 inhibitor (100 nM) (riboFECT™ CP Transfection Kit, RiboBio, China) on keratinocyte proliferation were explored according to the manufacturer’s instructions. The underlying mechanism of OA-RD17-induced proliferation against keratinocyte and macrophage was investigated using a specific MAPK signaling pathway inhibitor (10 μM, HY-N1966, MedChemExpress, USA). Furthermore, the mechanisms related to OA-RD17- and miR-632-induced proliferation of keratinocyte were detected using a specific TLR4 inhibitor (1 μM, HY-11109, MedChemExpress, USA) or β-catenin inhibitor (10 μM, HY-120697, MedChemExpress, USA).

### Cell migration

Trans-well assays were performed to investigate the effects of OA-RD17 (1 nM) on the migration capacity of keratinocytes, macrophages, primary macrophages, and primary keratinocytes according to previous research [2]. The effects of miR-632 mimic (50 nM), miR-632 inhibitor (100 nM) (riboFECT™ CP Transfection Kit, RiboBio, China), and OA-RD17 (1 nM) on keratinocyte migration were determined following the manufacturer’s instructions. Additionally, the mechanism of OA-RD17-induced macrophage migration was investigated using a specific MAPK signaling pathway inhibitor (10 μM, HY-N1966, MedChemExpress, USA).

### Colony formation

The effects of OA-RD17 (1 nM), miR-632 mimic (50 nM), and miR-632 inhibitor (100 nM) (riboFECT™ CP Transfection Kit, Reebok, China) on keratinocytes colony formation were determined according to previous study [40].

### Molecular docking

Molecular docking of OA-RD17 and the TLR4 was explored to investigate the possible mechanism underlying the pro-healing effects of OA-RD17. Robetta (http://www.robetta.org/) was used to download the X-ray crystal structure of OA-RD17. The structure of the TLR4 (PDB ID: 3fxi) was downloaded from the PDB database (http://www.rcsb.org/pdb). Molecular docking was performed using zdock, with the ligand set to flexible and acceptor set to rigid. Conformation with the highest score (affinity =  − 104 kcal/mol) was selected as the docking conformation and analyzed.

### Co-localization of TLR4 and OA-RD17 by confocal microscopy

Keratinocytes (2 × 10^3^ cells/well) were seeded in 12-well plates containing circle microscope cover glass (14 mm, Nest). After attachment, keratinocytes were treated with FITC labeled OA-RD17 (1 μM) for 1 h, and fluorescent staining was performed according to previous study [41]. Briefly, keratinocytes were incubated with TLR4 primary antibody (Affinity, China, 1:300) for 12 h at 4 °C, and then incubated with secondary antibody (Goat anti rabbit IgG Fluor594-conjugated, Affinity, China, 1:300) for 1 h at 37 °C. After DAPI staining and sealing, Zeiss Laser Confocal Microscope Imaging System (Zeiss LSM800, Germany) was used for observation.

### Enzyme-linked immunosorbent assay (ELISA)

ELISA was performed to explore the effects of OA-RD17 (1 nM) on the secretion of inflammatory factors in keratinocytes and macrophages as per previous research [1]. The detection of inflammatory factors, including tumor necrosis factor alpha (TNF-α), interleukin-1β (IL-1β), and interleukin-6 (IL-6), in cell supernatants was performed according to the ELISA kit instructions (NeoBioscience, Shanghai, China).

### RNA sequencing (RNA-seq)

Keratinocytes (2 × 10^6^ cells/well) were seeded in 6-well plates and treated with phosphate-buffered saline (PBS; vehicle) and OA-RD17 (1 nM) for 24 h. Total RNA was extracted using a total RNA extraction kit (Tiangen, Beijing, China) and the specimens were sent to Fruit Shell Biotechnology for RNA-seq. DEG-seq (differentially expressed genes (DEGs) from RNA-seq) with Q values < 0.05 were considered statistically significant. Geno Ontology (GO) analysis (http://www.geneontology.org/) was performed to characterize the biological processes, components, and functions of the DEGs. Furthermore, Kyoto Encyclopedia of Genes and Genomes (KEGG) enrichment analysis (http://www.genome.jp/KEGG/) was used to reveal the top 10 signaling pathways enriched in the DEGs to interpret the excellent pro-healing ability of OA-RD17.

### Dual luciferase reporter gene

The targeting relationship between hsa-miR-632 and GSK3β in keratinocytes was determined by Hanbio Biotechnology Co., Ltd. (Shanghai, China). Briefly, the wild-type (WT) or mutant region containing the has-miR-632 target was cloned into the pmiRGLO plasmid (Promega, Madison, WI, USA), named h-GSK3β-3UTR-WT and h-GSK3β-3UTR-MUT, respectively. Then, keratinocytes were transfected with h-GSK3β-3UTR-WT, h-GSK3β-3UTR-MUT, and hsa-miR632/negative control (NC), respectively (Hanbio Biotechnology Co., Ltd., China). After 48 h of transfection, the cells were collected to analyze luciferase activity according to the Promega Dual-Luciferase system and kit instructions. The firefly luciferase data served as an internal control and the detected Renilla luciferase data served as the dual fluorophore reporter gene fluorescence activity.

### RNA extraction and quantitative real-time polymerase chain reaction (RT-qPCR) assay

The expression levels of mRNAs and miRNAs were measured according to previous studies [17, 42]. Briefly, keratinocytes and macrophages (2 × 10^6^ cells/well) were seeded in 6-well culture plates. After adherence, cells were cultured in serum-free medium, and treated with vehicle, LPS (1 μg/mL), OA-RD17, TLR4 inhibitor, MAPK inhibitor, miR-632 Mimic, or miR-632 inhibitor for 24 h. Total RNA and miRNA of macrophages and keratinocytes were extracted using a total RNA extraction kit (Tiangen, Beijing, China) and miRNA extraction kit (Omega Bio-Tek, Shanghai, China), respectively. After reversal of miRNA and total RNA, RT-qPCR kits (GeneCopoeia, USA) were used to detect changes in miRNA and mRNA expression levels on a LightCycle 480 SYBR PCR instrument (Roche Diagnostics, Mannheim, Germany). The relative expression of miR-632 was analyzed using the 2^−ΔΔ^Ct method with U6 small nuclear RNA (snRNA) as the internal control, while IL-6, TNF-α, and IL-1β were analyzed with β-actin as the internal control, and Ki67, Cyclin A1, CDK2, GSK3β, β-catenin, Vimentin, c-MYC, and Cyclin D1 were analyzed with GAPDH as the internal control. The primers used in the study were provided in the Additional file 1: Table S2.

### Effects of OA-RD17 on healing of full-thickness skin wounds in mice

The therapeutic effects of OA-RD17 (1 nM) on full-thickness skin wounds in mice were assessed according to previous research [16]. Mice were randomly divided into four groups: i.e., vehicle (PBS), rh-bFGF (100 ng/mL), OA-RD17 (1 nM), and scrambled peptide (1 nM). OA-RD17 was dissolved in PBS, and then PBS (vehicle), rh-bFGF (positive control), OA-RD17 (1 nM), and scrambled peptide (an isotype control) were applied topically to wounds twice daily using a pipette (20 µL per time). The wound area was photographed every other day until day 8. Wound tissues were collected on postoperative days 4 and 8 for histopathological analysis.

### Effects of OA-RD17 on healing of deep second-degree burns in mice

Deep second-degree burns in mice were established to evaluate the therapeutic effects of OA-RD17 (1 nM) according to previous study [17]. Mice were randomly divided into three groups: i.e., vehicle (PBS), rh-bFGF (100 ng/mL), and OA-RD17 (1 nM). The wounds were treated twice daily by local application (20 µL per time) and photographed on postoperative days 0, 4, 8, 12, and 14. Wound tissue were collected on postoperative days 0, 4, 8, and 14 for histopathological analysis.

### Effects of OA-RD17 on healing of ex vivo skin wounds in diabetic patients

Skin tissues were donated from diabetic patients (49–62 years), with details shown in Additional file 1: Table S3. The therapeutic efficacy of OA-RD17 (1 nM) on diabetic patient ex vivo skin wounds was investigated with reference to previous studies [43, 44]. Briefly, the donated tissues were divided into circular pieces using a 6-mm diameter biopsy device, and 2-mm diameter full-thickness wounds were created in the center of each tissue sample. The modeled skin tissues were randomly divided into three groups: i.e., vehicle (PBS), rh-bFGF (100 ng/mL), and OA-RD17 (1 nM). Skin wounds were topically treated daily (20 µL of treatment), and skin tissues were collected for histopathological analysis on postoperative days 3 and 7.

### Effects of miR-632 on healing of full-thickness skin wounds in SD rats

The therapeutic effects of miR-632 on full-thickness skin wounds in SD rats were validated according to previous study [45]. MiR-632 agomir NC (5 nM) and miR-632 agomir (5 nM, micrON™ miRNA agomir, RiboBio, China) were injected on postoperative days 0 and 4, respectively, and wound conditions were recorded on day 9 after treatment. Dorsal skin wound samples were collected on postoperative day 9 for histopathological analysis.

### Hematoxylin and eosin (H&E) staining

To investigate tissue regeneration of dorsal skin wounds in mice, ex vivo skin wounds in diabetic patients, and dorsal skin wounds in rats after different treatments, H&E staining analysis was performed according to previous study [16]. Light microscopy (Primo Star, Zeiss, Germany) was used at equal magnification (× 40) to record tissue regeneration in skin wounds. To evaluate the regenerated epidermis and granulation tissue of skin wounds, the thickness of five areas of wound epidermis and granulation tissue were randomly measured using ImageJ software and analyzed by GraphPad Prism software.

### Immunohistochemistry

Immunohistochemical staining was performed to investigate epidermal cell proliferation and inflammatory factors expression in regenerating skin tissue, as per previous study [46]. Primary antibodies, including rabbit anti-mouse IL-1β (Affinity, af5103, China, 1:100), IL-6 (Servicebio, gb11117, China, 1:600), TNF-α (Servicebio, gb11188, China, 1:500), and Ki67 (Servicebio, gb11499, China, 1:500) were used following the provided instructions. After incubation, the skin wound tissue was incubated with secondary antibodies (Servicebio, GB23303, China, 1:400) for 1 h at 37 °C, then sealed and observed by light microscopy (Primo Star, Zeiss, Germany). ImageJ software was used to measure positive staining intensity and analyzed by GraphPad Prism software.

### Immunofluorescence

The wound tissues were sectioned (5 μm), sequentially deparaffinized, rehydrated, antigen repaired and stained. After blocking with 5% (w/W) goat serum containing 0.3% TritonX-100 for 1 h, the tissues were incubated using primary antibody (rat anti-mouse) F4/80 (Abcam, ab6640, USA; 1:100) with rabbit anti-mouse iNOS (Abcam, ab178945, USA; 1:300) and Arg-1 (Affinity, df6657, China, 1:200), respectively, for counterstaining at 4 °C for 12 h. The tissues were then incubated with secondary antibodies goat anti-rabbit IgG Alexa Fluor^®^ 488 (Abcam, ab150077, USA; 1:200) and goat anti-rat IgG Alexa Fluor^®^ 647 (Abcam, ab150159, USA; 1:200) at 37 °C for 1 h, then DAPI stained and recorded using the Zeiss Laser Confocal Microscope Imaging System (Zeiss LSM800, Germany).

### Western blotting

After incubation with PBS, OA-RD17 (1 nM), lipopolysaccharide (LPS, 1 μg/mL) (Solarbio, China), specific TLR4 inhibitor (1 μg/mL), miR-632 mimic (50 nM), or miR-632 inhibitor (100 nM) for 24 h, respectively, cell lysates (RIPA: PMSF: phosphatase inhibitor = 100:1:1; RIPA and PMSF, Meilun Biotechnology, Dalian, China; phosphatase inhibitor, Roche, Shanghai, China) were used to extract total protein in keratinocytes and macrophages. Moreover, cell lysates were also used to extract total protein in wound tissues from SD rats. The extracted proteins were quantified using the Bradford method (BCA protein analysis kit, Meilun, Dalian, China). The protein samples were then separated by 10% sodium dodecyl sulfate–polyacrylamide gel electrophoresis (SDS-PAGE), electro-imprinted on polyvinylidene fluoride membranes, and recorded and analyzed quantitatively using the Bole exposure software system. Primary antibodies, including GAPDH, Lamin B1, P38, P-P38, ERK, P-ERK, JNK, P-JNK, IκB, P-IκB, P65, P-P65 (Affinity, China), GSK3β, β-catenin, Cyclin D1, c-MYC, and Vimentin (ZEN BIO, China) were used following the provided instructions.

## Results

### Structural characteristics of OA-RD17

The prepropeptide identified from *Odorrana andersonii* skin contained 64 amino acid residues and encoded by a 363-bp cDNA sequence. The mature peptide amino acid sequence was ‘RDYCTPEDCDYDFSFPI’ (Additional file 1: Fig. S1A). Searching for sequence similarities between ‘RDYCTPEDCDYDFSFPI’ and peptides reported in the NCBI database (data not shown), we found this peptide to be novel and thus named it OA-RD17 (OA, abbreviation of species name; RD, two initial amino acids; 17, length of peptide). As shown in Additional file 1: Fig. S1B, the overall structures of OA-RD17 and RL-QN15, another pro-healing peptide identified in our previous research [16], showed high similarities, but their mature peptides showed marked differences. The PeptideMass database indicated that the molecular weight of OA-RD17 was 2 084.23 Da. The amino acid composition and advanced structure of OA-RD17 are shown in Additional file 1: Fig. S1C. The advanced structure of OA-RD17 revealed the formation of an intramolecular disulfide bond between serine residues at positions 4 and 9 (Additional file 1: Fig. S1D, E). Furthermore, OA-RD17 showed no hemolytic activity against Kunming mouse red blood cells and no acute toxicity against Kunming mice, exhibiting excellent biocompatibility (Additional file 1: Fig. S2, Table S1).

### OA-RD17 markedly facilitated keratinocyte and macrophage proliferation and migration

We firstly investigated the pro-healing activity and sequence specificity of OA-RD17 at the cellular level, focusing on keratinocytes and macrophages, which play important roles in wound healing. The promoting keratinocyte scratch repair activity of OA-RD17 was higher than that of PBS, rh-bFGF, and scrambled peptide (an isotype control) at all time (Additional file 1: Fig. S3A, B). As shown in Additional file 1: Fig. S3C, D, OA-RD17 (1 nM) markedly enhanced keratinocyte migration, with migrating cell numbers 2.45 and 2.42 times higher than that in the vehicle and scrambled peptide groups, respectively. OA-RD17 also promoted keratinocyte proliferation in a concentration-dependent manner, showing significantly greater activity than the vehicle at a concentration of 1 nM (Additional file 1: Fig. S3E). However, scrambled peptide did not exhibit pro-proliferative activity on keratinocyte (Additional file 1: Fig. S3F). Compared to the vehicle and scrambled peptide, OA-RD17 also significantly promoted colony formation of keratinocyte (Additional file 1: Fig. S3G–H). Additionally, OA-RD17 significantly promoted macrophage proliferation and migration (Additional file 1: Fig. S3I–K). More importantly, OA-RD17 (1 nM) also significantly promoted the migration and proliferation of primary mouse keratinocyte and macrophage (Additional file 1: Fig. S4A–F). In conclusion, OA-RD17 exhibited specific promoting activity on the proliferation and migration of macrophage and keratinocyte, and showed potent pro-healing activity.

### OA-RD17 significantly accelerated regeneration of full-thickness wounds in mice

**Fig. 1:**
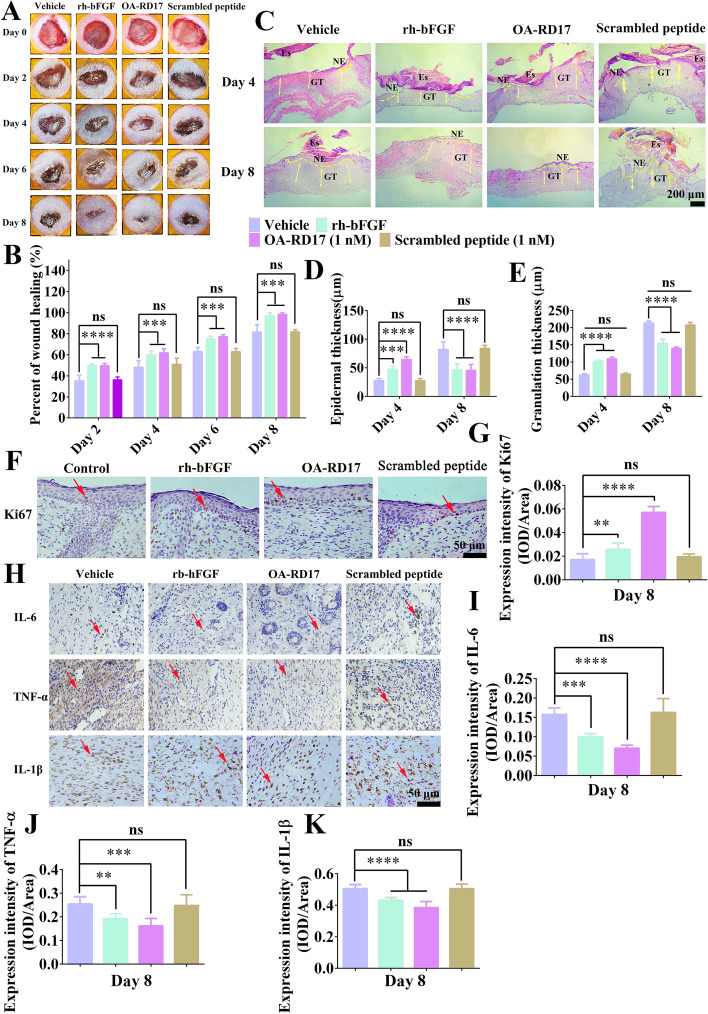
OA-RD17 inhibited inflammation, promoted re-epithelialization and granulation regeneration, and accelerated full-thickness wound healing in mice. **A** Representative images of full-thickness wound healing in mice after local application of PBS, rh-bFGF (100 ng/mL), OA-RD17 (1 nM), or scrambled peptide (1 nM) on postoperative days 0, 2, 4, 6, and 8. **B** Quantification of OA-RD17 on full-thickness wound regeneration in mice on postoperative days 2, 4, 6, and 8. **C** Representative H&E staining of wound tissue in mice on postoperative days 4 and 8. Yellow dotted lines represent areas of neoepithelium; Es: eschar; NE: neoepithelium; GT: regenerated granulation tissue; Yellow arrows represent quantified areas of regenerated granulation tissue; scale bar 200 μm. **D**, **E** Quantification of neo-epidermal and regranulation tissue thickness on postoperative days 4 and 8. **F** Representative images of immunohistochemical staining of epidermal cell proliferation factor Ki67 expression in epidermal region of wound tissues on postoperative day 8; red arrows indicate positive staining, scale bar 50 μm. **G** Quantitative expression of epidermal cell proliferation factor Ki67; positive expression is defined as intensity of positive staining per unit area. **H** Representative immunohistochemical images of inflammatory factors IL-6, TNF-α, and IL-1β in wound area of mice on postoperative day 8; red arrows indicate positive staining, scale bar 50 μm. **I**-**K** Quantification of IL-6, TNF-α, and IL-1β expression in wound area on day 8 after treatment; positive expression is defined as intensity of positive staining per unit area. All data are expressed as mean ± SEM from three independent experiments performed in quintuplicate; ns, no significance; **P < 0.01, ***P < 0.001, and ****P < 0.0001 indicate statistically significant difference compared to vehicle

As OA-RD17 exhibited excellent pro-healing activity at the cellular level, we next explored its pro-regeneration capacity at the animal level. After 8 days of OA-RD17 treatment, the skin wound restoration rate in OA-RD17-treated mice approached 100%, and significantly higher than the vehicle and scrambled peptide (Fig. 1A, B). The skin wounds gradually regenerated new epidermis and granulation tissue after application of PBS, rh-bFGF, OA-RD17, and scrambled peptide (Fig. 1C). The PBS- and scrambled peptide-treated mice still had scabs and thicker neoepidermis and granulation tissues, while the rh-bFGF- and OA-RD17-treated mice had significantly thinner neoepidermis, approaching to that of normal mice (Fig. 1C–E). The new granulation tissue in OA-RD17-treated mice was significantly thinner than that in the rh-bFGF-treated mice, suggesting that OA-RD17 may suppress excessive granulation tissue regeneration, thereby preventing excessive scar formation (Fig. 1D, E). In addition, OA-RD17 treatment for 8 days resulted in a significant increase in neoepidermis Ki67 expression, which also demonstrated its pro-epidermal regenerative activity (Fig. 1F, G).

Immunohistochemical analysis showed that, compared to PBS and scrambled peptide, rh-bFGF and OA-RD17 treatment significantly reduced the expression of inflammatory factors IL-6, IL-1β, and TNF-α on postoperative day 8, with the lowest expression after OA-RD17 treatment (Fig. 1H–K). Immunofluorescence staining of macrophage phenotypes revealed the mechanism of OA-RD17 modulating inflammation in the skin wound (Additional file 1: Fig. S5A). Compared with PBS, scrambled peptide, and rh-bFGF treatment, OA-RD17 significantly decreased the number of MI phenotype macrophages and significantly increased the number of MII phenotype macrophages in the regenerated skin tissue (Additional file 1: Fig. S5B, C). Macrophage polarization to the MII phenotype can inhibit the excessive inflammation and promoted wound healing [47]. In summary, OA-RD17 showed specific effects on wound healing, which reduced inflammatory responses by regulating macrophage polarization, and promoted re-epithelialization and granulation tissue regeneration to accelerate wound healing.

### OA-RD17 markedly promoted skin regeneration of deep second-degree burns in mice

**Fig. 2:**
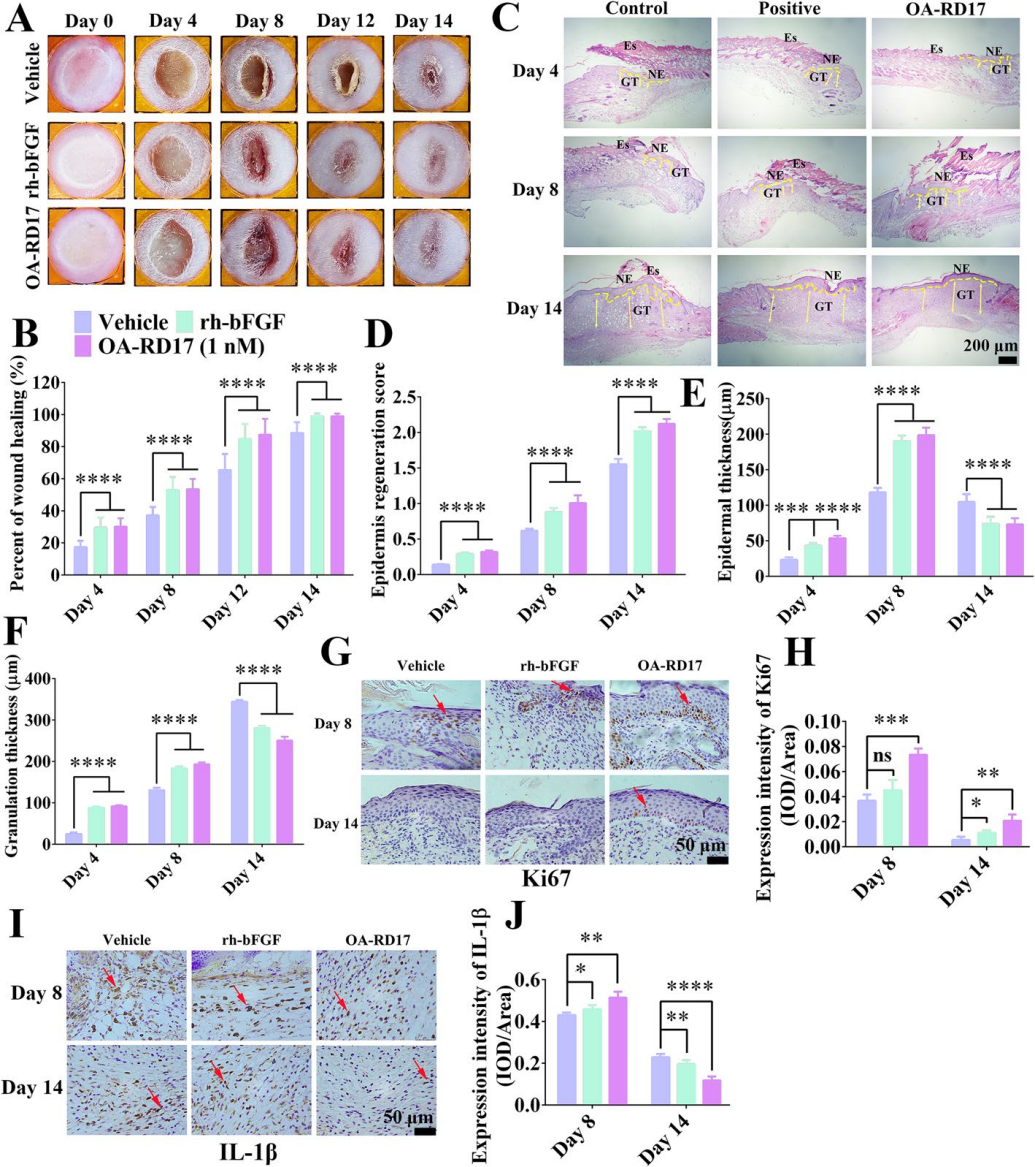
OA-RD17 promoted macrophage polarization to reduce inflammation and epidermal regeneration to accelerate deep second-degree burn healing in mice. **A** Representative images of deep second-degree burn wound recovery on days 0, 4, 8, 12, and 14 in mice locally treated with PBS, rh-bFGF (100 ng/mL), or OA-RD17 (1 nM). **B** Quantification of deep second-degree burn wound repair on days 4, 8, 12, and 14 in mice. **C** Representative H&E staining of deep second-degree burn wounds on days 4, 8, and 14 after treatment with PBS (vehicle), rh-bFGF (100 ng/mL), or OA-RD17 (1 nM) in mice. Yellow dotted lines represent areas of neoepithelium; Es: eschar; NE: neoepithelium; GT: regenerated granulation tissue; Yellow arrows represent quantified areas of regenerated granulation tissue; scale bar 200 μm. **D**-**F** Quantification of regenerated epidermal scores, regenerated epidermal thickness, and regenerated granulation tissue in burned skin areas in mice. **G** Representative images of Ki67 immunohistochemical staining of regenerated epidermis in wound area on days 8 and 14 after treatment with PBS, rh-bFGF (100 ng/mL), or OA-RD17 (1 nM). Red arrows indicate positive staining, scale bar 50 μm. **H** Quantification of Ki67 expression in regenerated epidermis of wound area on days 8 and 14. **I** Representative images of immunohistochemical staining of inflammatory factor IL-1β in wound area on days 8 and 14 after treatment with PBS, rh-bFGF (100 ng/mL), or OA-RD17 (1 nM). Red arrows indicate positive staining, scale bar 50 μm. **J** Quantification of IL-1β expression in wound area; positive expression is defined as intensity of positive staining per unit area. All data are expressed as mean ± SEM from three independent experiments performed in quintuplicate; ns, no significance, *P < 0.05, **P < 0.01, ***P < 0.001, and ****P < 0.0001 indicate statistically significant difference compared to vehicle

OA-RD17 regulated inflammation to accelerate full-thickness skin wound regeneration in mice, we next investigated the treatment of OA-RD17 on deep second-degree burns, which accompanied by a persistent inflammatory response. Compared to normal skin, the epidermis and dermis of mouse burned skin were disrupted, showing edema, thickening and the disappearance of all appendages, and indicating the successful establishment of a deep second-degree burn model (Additional file 1: Fig. S6). After two weeks of treatment, the wound repair rate in PBS-treated mice reached 88.5%, with eschar still present (Fig. 2A, B). After rh-bFGF treatment, small red hyperplastic granulation scars were present in the central part of the wound, whereas OA-RD17 treatment had restored the wound to almost normal skin and no scar hyperplasia was observed (Fig. 2A, B). After 14 days of OA-RD17 treatment, the damaged epidermal and dermal structures were rehabilitated, and neo-epidermal thickness and regenerated granulation tissue approached normal skin thickness (Fig. 2C–F). Compared to rh-bFGF- and PBS-treated groups, OA-RD17 significantly promoted the expression of Ki67 in neoepidermis (Fig. 2G–H).

The expression of IL-1β, TNF-α, and IL-6 in wound tissue were higher in the OA-RD17- and rh-bFGF-treated groups than in the vehicle (PBS) group on day 8 post-operation (Fig. 2I, J, Additional file 1: Fig. S7A–C). However, OA-RD17 treatment showed the lowest expression of inflammatory factors after 14 days of treatment, indicating its inhibitory activity against excessive inflammation (Fig. 2J, S7B-C). Since OA-RD17 modulated macrophage polarization to reduce inflammation (Additional file 1: Fig. S5), we next investigated whether OA-RD17 regulated differential expression of inflammatory factors by macrophage polarization (Additional file 1: Fig. S8A, D). OA-RD17 treatment for 8 days significantly enhanced the positive staining intensity of MI macrophages compared to the PBS- and rh-bFGF-treated groups; in contrast, positive-staining intensity for MII macrophages was weak in all treatment groups, with no significant differences (Additional file 1: Fig. S8B, C). After 14 days of treatment, the OA-RD17-treated group showed lower positive-staining intensity for MI macrophages but higher positive-staining intensity for MII macrophages compared to the PBS- and rh-bFGF-treated groups (Additional file 1: Fig. S8E, F).

### OA-RD17 exhibited excellent therapeutic effects on ex vivo skin wounds in diabetic patients

**Fig. 3:**
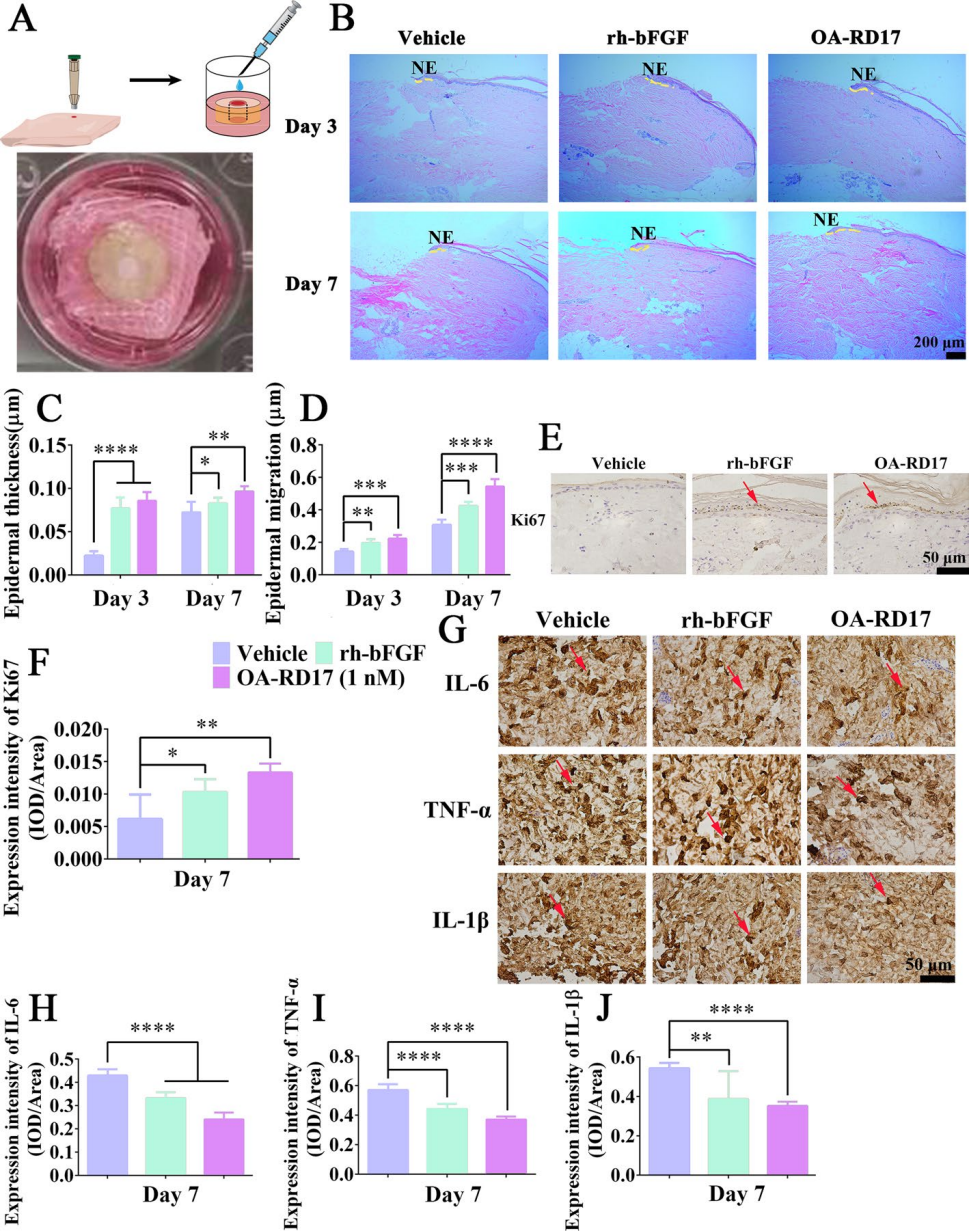
OA-RD17 significantly decreased inflammation and promoted epidermal regeneration in diabetic patient ex vivo skin wounds. **A** Illustration of diabetic patient ex vivo skin wound model. **B** Representative images of H&E staining of diabetic patient ex vivo skin wound tissues on days 3 and 7. Yellow dotted lines represent areas of neoepithelium; NE: neoplastic epithelium; scale bar 200 μm. **C** Thickness variations in neodermis in wound tissue on days 3 and 7 of OA-RD17 treatment. **D** Epidermal migration length variation in neodermis in wound tissue on days 3 and 7 of OA-RD17 treatment. **E** Representative images of immunohistochemical staining for epidermal Ki67 expression after one week of OA-RD17 treatment; positive staining is indicated by red arrows, scale bar 50 μm. **F** Quantification of positive-staining intensity of Ki67 expression in epidermis. **G** Representative images of immunohistochemical staining for inflammatory factors IL-6, TNF-α, and IL-1β in wound area on day 7 of OA-RD17 treatment; positive staining is indicated by red arrows, scale bar 50 μm. **H-J** Quantification of intensity of IL-6, TNF-α, and IL-1β expression in wound area. All data are expressed as mean ± SEM from three independent experiments performed in triplicate. *P < 0.05, **P < 0.01, ***P < 0.001, and ****P < 0.0001 indicate statistically significant difference compared to vehicle

Next, we established a diabetic patient ex vivo skin wound model to investigate the therapeutic effects of OA-RD17 on human skin tissues (Fig. 3A). OA-RD17 treatment significantly promoted epidermal regeneration and migration towards the center of the wound (Fig. 3B). After one week of OA-RD17 treatment, epidermal thickness and migration length reached 0.09 μm and 0.54 μm, respectively, 1.28- and 1.16-fold greater than that of rh-bFGF treatment (Fig. 3C, D). OA-RD17 treatment significantly promoted epidermal Ki67 expression, indicating that OA-RD17 promoted epidermal regeneration by enhancing epidermal cell proliferation (Fig. 3E, F). A persistent inflammatory response is a major obstacle in diabetic foot ulcer healing [3]. Hence, immunohistochemical staining was performed to reveal the effects of OA-RD17 on inflammatory factors expression in diabetic patient ex vivo skin wounds (Fig. 3G). Inflammatory factors (IL-6, TNF-α, and IL-1β) expression decreased under rh-bFGF and OA-RD17 treatment compared to PBS treatment, with the OA-RD17-treated group showing the lowest levels (Fig. 3H–J). In conclusion, OA-RD17 modulated inflammation, promoted the regeneration of epidermal and granulation tissue, and significantly accelerated the healing of mice and diabetic patient ex vivo skin wounds.

### OA-RD17 inhibited NF-κB to reduce inflammatory factors release and activated MAPK to promote macrophage proliferation and migration

**Fig. 4:**
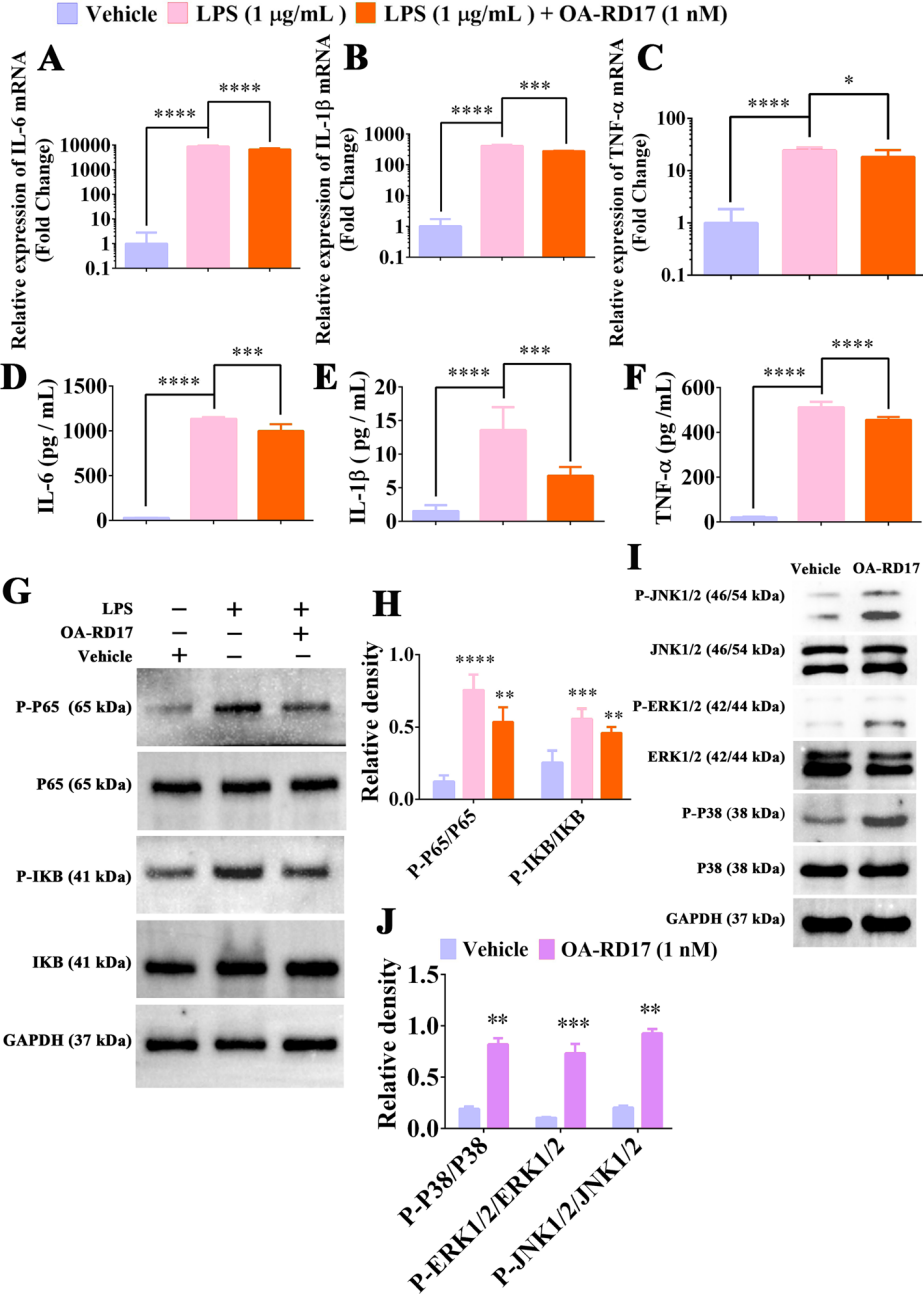
OA-RD17 inhibited NF-κB activation to suppress inflammatory factors expression and activated MAPK to promote macrophage proliferation and migration. **A**-**C** RT-qPCR was performed to detect changes in mRNA expression levels of IL-6, IL-1β, and TNF-α in macrophages treated with PBS, LPS, or LPS + OA-RD17. Relative expression was calculated using β-actin as an internal reference. **D**-**F** ELISA was conducted to determine IL-6, IL-1β, and TNF-α expression in macrophages treated with PBS, LPS, or LPS + OA-RD17. **G** OA-RD17 inhibited activation of NF-κB signaling pathway induced by LPS in macrophages. **H** Quantification of phosphorylation of P65 and IκB. **I** Western blotting was performed to detect effects of OA-RD17 treatment for 24 h on MAPK signaling pathway in macrophages. **J** Quantification of key proteins of macrophage MAPK signaling pathway (phosphorylated proteins of P38, ERK, and JNK). All data are expressed as mean ± SEM from three independent experiments, *P < 0.05, **P < 0.01, ***P < 0.001, and ****P < 0.0001

As OA-RD17 regulated the polarization of macrophages to reduce the inflammation in skin wounds, we next investigated the mechanism by which OA-RD17 regulated macrophage inflammatory factors expression. The expression of IL-6, IL-1β, and TNF-α in macrophage were significantly increased after LPS stimulation, but were reduced to 0.76-, 0.67-, and 0.75-fold of that of LPS treatment following the treatment of OA-RD17 (Fig. 4A–C). The expression of IL-6, IL-1β and TNF-α in LPS-treated macrophages was 1133.0 pg/mL, 13.5 pg/mL, and 511.4 pg/mL, whereas OA-RD17 significantly reduced the increased expression of IL-6, IL-1β and TNF-α (997.9 pg/mL, 6.7 pg/mL, and 455.1 pg/mL) induced by LPS stimulation (Fig. 4D–F).

The expression of IL-6, IL-1β and TNF-α is tightly associated to the NF-κB signaling pathway, therefore we determined the relationship between the inhibitory effects of OA-RD17 on inflammatory factor expression and the NF-κB signaling pathway (Fig. 4G). Phosphorylation of P65 and IκB increased significantly in macrophages stimulated by LPS (Fig. 4H). However, OA-RD17 significantly inhibited LPS-induced activation of the NF-κB signaling pathway, with phosphorylated P65 and IκB expression reduced to 0.71- and 0.82-fold that of LPS treatment, respectively (Fig. 4H). The above results indicated that OA-RD17 may inhibit the NF-κB signaling pathway, thereby suppressing the expression of inflammatory factors in macrophages.

The MAPK signaling pathway is tightly associated with cell proliferation and migration [48]. As shown in Fig. 4I-J, OA-RD17 significantly promoted the expression of phosphorylated P38, ERK, and JNK, indicating that OA-RD17 activated the MAPK signaling pathway in macrophages. HY-N1966, a specific inhibitor of MAPK signaling pathway, significantly reduced the promotion effects of OA-RD17 on macrophage proliferation and migration, suggesting that OA-RD17 promotes macrophage proliferation and migration via activation of the MAPK signaling pathway (Additional file 1: Fig. S9A–C).

### Analysis of RNA-seq

As the major cellular component of the epidermis, keratinocytes not only exhibit immune functions but are also the executors of the re-epithelialization, which is essential for wound healing [26]. Therefore, RNA-seq was used to reveal the molecular mechanism of OA-RD17 on keratinocytes and wound repair, which might contribute to the discovery of new drug targets and the development of novel wound repair agents. At the mRNA level, OA-RD17-regulated differentially expressed genes (DEGs), associated with ribosomal and extracellular components, and involved in nucleic acid binding, intranuclear trafficking of proteins and phosphorylation biological processes (Additional file 1: Fig. S10A–C). KEGG pathway enrichment analysis showed that OA-RD17-regulated DEGs were involved in signaling pathways related to cell proliferation and migration, providing strong evidence for the promotion of keratinocyte proliferation and migration by OA-RD17 (Additional file 1: Fig. S10D). Notably, OA-RD17 also up-regulated eight and down-regulated four differentially expressed miRNAs, which were involved in composition of the extracellular matrix and transduction factor complex as well as biological processes such as protein phosphorylation, signal transduction, and cell growth (Additional file 1: Fig. S11A–C). KEGG pathway enrichment analysis indicated that the miRNAs were involved in the signaling pathways related to cell proliferation and migration, such as MAPK, focal adhesion, and Wnt signaling pathways (Additional file 1: Fig. S11D).

### OA-RD17 reduced inflammatory factors release and activated MAPK signaling pathway to promote keratinocyte proliferation and migration

**Fig. 5:**
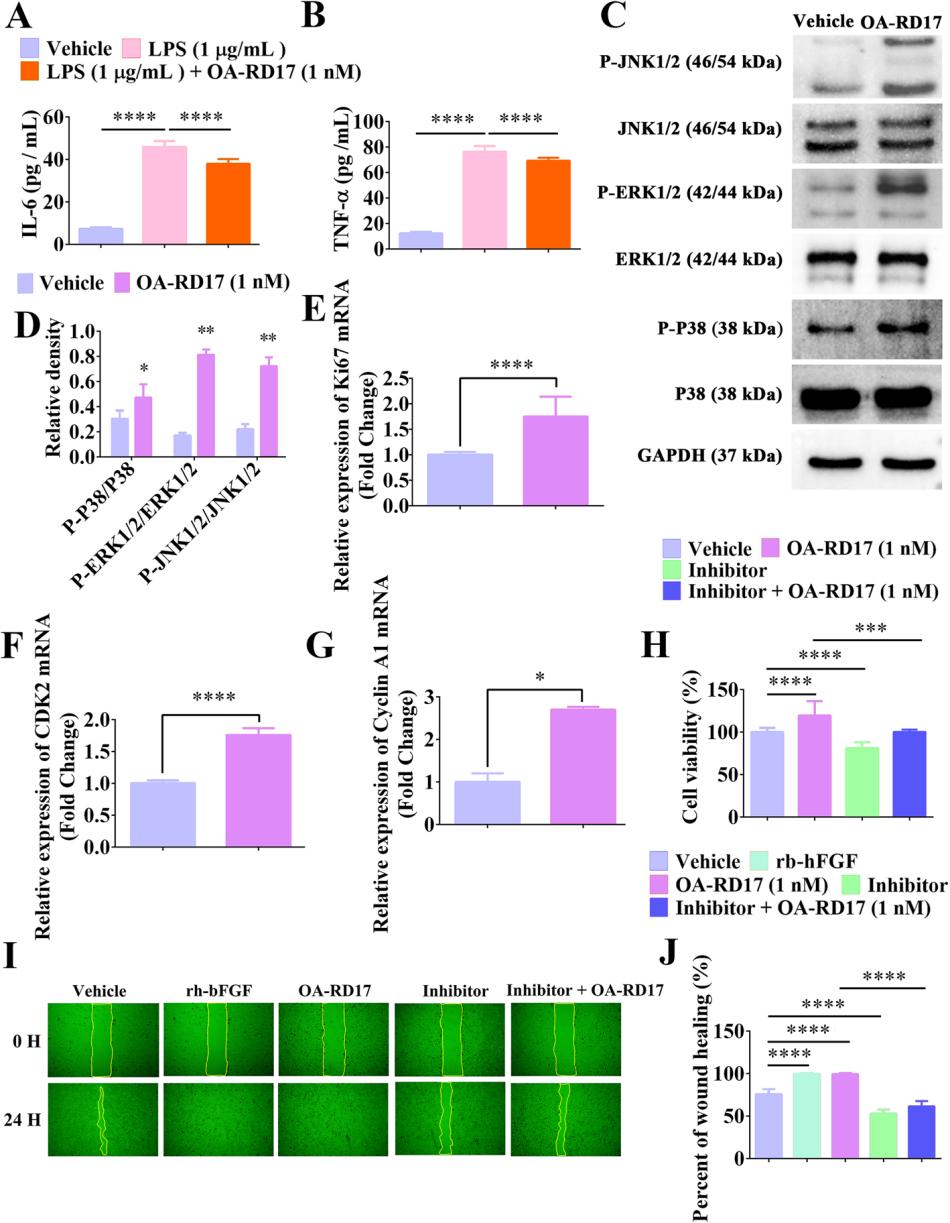
OA-RD17 suppressed inflammatory factor expression and activated the MAPK signaling pathway to promote keratinocyte proliferation and migration. **A**, **B** ELISA analysis of effects of OA-RD17 on inflammatory factors (IL-6 and TNF-α) release in keratinocytes after LPS stimulation. **C** Representative image of MAPK signaling pathway activation in keratinocytes after 24 h of OA-RD17 treatment, determined by western blotting. **D** Quantification of P38, ERK, and JNK protein phosphorylation. **E**–**G** RT-qPCR analysis of Ki67, CDK2, and Cyclin A1 mRNA expression in OA-RD17-treated keratinocytes. **H** Changes in pro-proliferation activity of OA-RD17 in keratinocytes after application of MAPK signaling pathway inhibitors. **I** Representative images of effects of OA-RD17 on keratinocyte scratch repair after application of MAPK signaling pathway inhibitors. **J** Quantification of effects of OA-RD17 on keratinocyte scratch repair rate after application of MAPK signaling pathway inhibitors. All data are expressed as mean ± SEM from three independent experiments, *P < 0.05, **P < 0.01, ***P < 0.001, and ****P < 0.0001

Keratinocytes are not only immune regulatory but also play a role in the re-epithelialization process [4]. As OA-RD17 regulated the expression of inflammatory factors in macrophages, we also explored the effects of OA-RD17 on the expression of inflammatory factors in keratinocytes. LPS stimulation increased the secretion of IL-6 and TNF-α in keratinocytes, whereas OA-RD17 significantly decreased their expression (Fig. 5A, B). RNA-seq indicated that OA-RD17-regulated DEGs mediated various signaling pathways associated to cell proliferation and migration, including MAPK signaling pathway, thus we explored the effect of OA-RD17 on MAPK signaling (Additional file 1: Fig. S10D, S11D). Compared with the PBS-treated group, OA-RD17 significantly facilitated the expression of phosphorylated P38, ERK, and JNK, suggesting that OA-RD17 significantly activated the MAPK signaling pathway in keratinocytes (Fig. 5C, D). OA-RD17 also promoted the expression of Ki67, CDK2, and Cyclin A1 in keratinocytes, indicating the pro-proliferative activity of OA-RD17 (Fig. 5E–G). In addition, inhibitors of the MAPK signaling pathway significantly decreased the promoting proliferation and scratch repair abilities of OA-RD17 on keratinocytes, indicating that OA-RD17 promoted keratinocyte proliferation and migration by activating MAPK signaling pathway (Fig. 5H–J). Thus, OA-RD17 regulated the secretion of inflammatory factors from keratinocytes and promoted keratinocyte proliferation and migration via activation of the MAPK signaling pathway.

### OA-RD17 activated the MAPK signaling pathway via TLR4 in keratinocytes

**Fig. 6:**
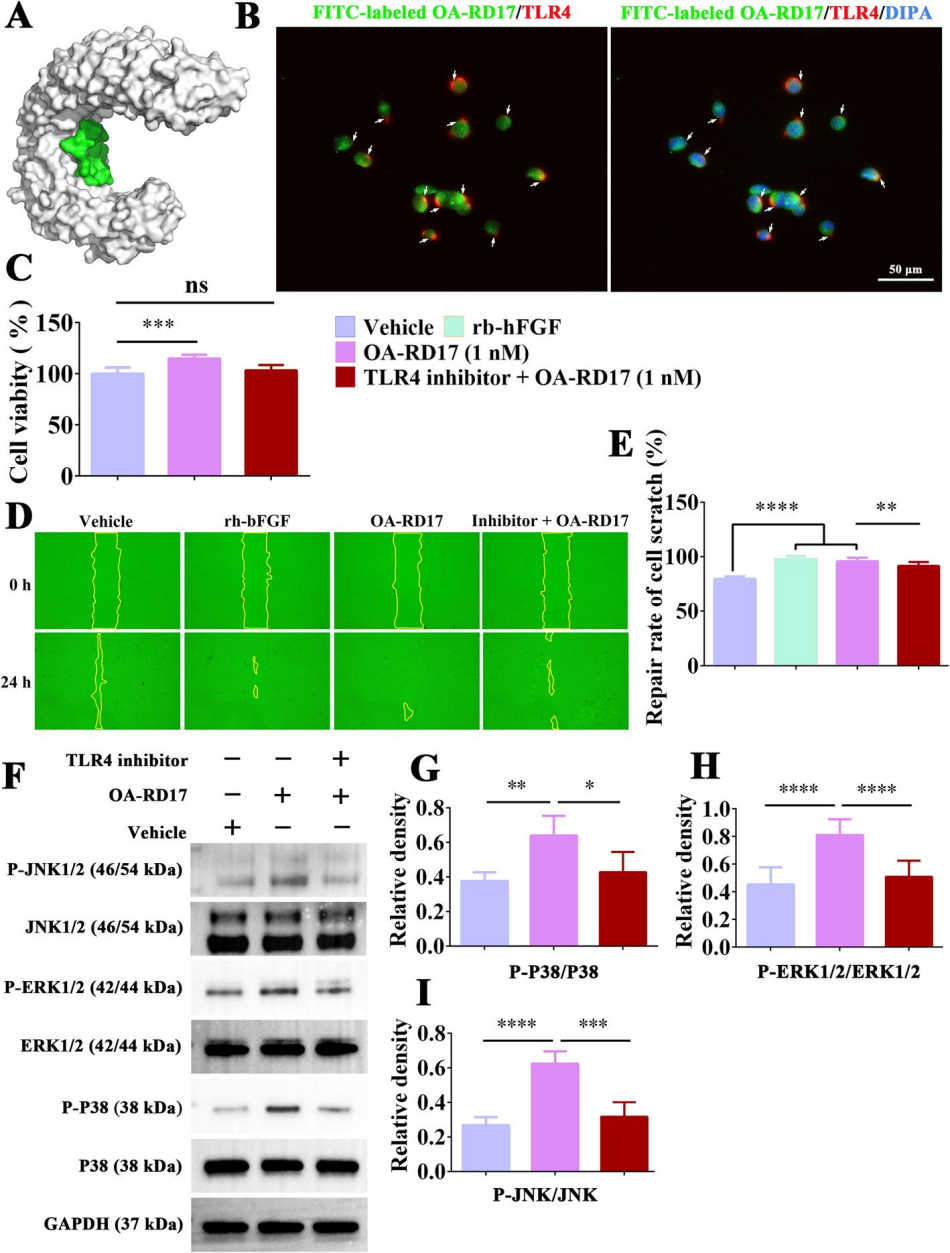
OA-RD17 promoted keratinocyte proliferation and migration via MAPK signaling pathway activation by TLR4. **A** Representative image of molecular docking of OA-RD17 with TLR4. **B** Representative images of immunofluorescence for co-localization of TLR4 and OA-RD17 in keratinocytes after treatment with FITC-labeled OA-RD17 for 1 h; green fluorescence for FITC-labeled OA-RD17, red fluorescence for TLR4, and DAPI blue for nuclei; the white arrows indicated the binding of TLR4 and OA-RD17; scale bar 50 µm. **C** Changes in pro-proliferative activity of OA-RD17 on keratinocytes 24 h after TLR4 inhibition. **D** Representative images of the effects of OA-RD17 on keratinocyte scratch healing after TLR4 inhibitor treatment. **E** Quantification of pro-keratinocyte scratch repair by OA-RD17 after TLR4 inhibition. **F** Western blot analysis of MAPK signaling pathway activation in OA-RD17-treated keratinocytes 24 h after TLR4 inhibition. **G**–**I** Quantification of OA-RD17-activated MAPK signaling pathway (P38, ERK, and JNK phosphorylation) in keratinocytes. All data are expressed as mean ± SEM from three independent experiments, ns, no significance, *P < 0.05, **P < 0.01, ***P < 0.001, and ****P < 0.0001

Peptides and proteins usually exert their functions by entering cells directly or by interacting with receptors to deliver extracellular signals for biological functions [49]. Molecular docking results showed that OA-RD17 could bound to the extracellular region of the TLR4 on the inner side of the cavity via hydrogen bonding and electrostatic interactions (Fig. 6A, Additional file 1: Fig. S12A–C). More importantly, co-localization of TLR4 and OA-RD17 pointed out that green fluorescence labeled OA-RD17 could bind to red fluorescence labeled TLR4 in keratinocytes, and the binding region was located outside the nucleus, suggesting that OA-RD17 may exert biological functions via TLR4 (Fig. 6B). Furthermore, the pro-keratinocyte proliferative activity of OA-RD17 was inhibited by the TLR4 inhibitor (Fig. 6C). The pro-keratinocyte scratch repair activity of OA-RD17 was also significantly suppressed by TLR4 inhibitor (Fig. 6D, E). The above results demonstrated that OA-RD17 facilitated keratinocyte proliferation and migration via the TLR4.

To investigate whether OA-RD17 promotes keratinocyte proliferation and migration via activation of the MAPK signaling pathway by the TLR4, western blotting was performed to detect the activation of MAPK signaling pathway in keratinocytes by OA-RD17 after treatment with TLR4 inhibitor (Fig. 6F). Compared to PBS treatment, OA-RD17 promoted the expression of phosphorylated P38, ERK, and JNK, whereas TLR4 inhibitor significantly suppressed the phosphorylation of P38, ERK, and JNK induced by OA-RD17 (Fig. 6G–I). In conclusion, OA-RD17 promotes keratinocyte proliferation and migration via activation of the MAPK signaling pathway by the TLR4.

### OA-RD17 upregulated miR-632 expression, and overexpression of miR-632 facilitated keratinocyte proliferation and migration

Based on RNA-seq, miR-632 showed significant and stable up-regulation under OA-RD17 treatment, suggesting that it may play an important role in the wound repair (Additional file 1: Fig. S11A). RT-qPCR also demonstrated that OA-RD17 (1 nM) significantly up-regulated miR-632 expression (3.9-fold) in keratinocytes compared with the PBS-treated group, consistent with the RNA-seq results (Additional file 1: Fig. S13A). Although miR-632 is known to promote cell proliferation and migration [31, 50], its effects on keratinocytes remain unknown. Therefore, we investigated the role of miR-632 in keratinocytes. MiR-632 mimic significantly upregulated miR-632 expression and promoted keratinocyte scratch repair compared to miR-632 mimic NC (Additional file 1: Fig. S13B–D). Overexpression of miR-632 also significantly promoted keratinocyte proliferation (Additional file 1: Fig. S13E). Furthermore, overexpression of miR-632 significantly promoted keratinocyte migration, consistent with the cell scratch assay (Additional file 1: Fig. S13F–G). Overexpression of miR-632 significantly promoted keratinocyte colony formation and exhibited considerable pro-proliferative effects (Additional file 1: Fig. S13H, I). Notably, overexpression of miR-632 significantly promoted Ki67, CDK2, and Cyclin A1 expression, which also demonstrated the pro-keratinocyte proliferation activity of miR-632 (Additional file 1: Fig. S13J–L).

### Down-regulation of miR-632 expression significantly inhibited keratinocyte proliferation and migration, while OA-RD17 mitigated its inhibitory effects

To systematically investigate the proliferative and migratory effects of miR-632 on keratinocytes and the regulation of miR-632 by OA-RD17, we inhibited miR-632 expression and treated with OA-RD17 to investigate their effect on the keratinocytes. The miR-632 inhibitor markedly reduced the expression of miR-632 in keratinocytes, while OA-RD17 inhibited the down-regulation of miR-632 expression by the miR-632 inhibitor (Additional file 1: Fig. S14A). Down-regulation of miR-632 expression significantly inhibited keratinocyte scratch repair (Additional file 1: Fig. S14B, C). However, the addition of OA-RD17 after down-regulation of miR-632 expression promoted scratch repair, suggesting that OA-RD17 may promote scratch repair through activation of the MAPK signaling pathway or up-regulation of miR-632 expression (Additional file 1: Fig. S14B, C). Similarly, down-regulation of miR-632 expression inhibited keratinocyte migration, whereas OA-RD17 significantly mitigated the inhibition of keratinocyte migration caused by down-regulation of miR-632 expression (Additional file 1: Fig. S14D, E). Down-regulation of miR-632 also significantly inhibited keratinocyte colony formation, while OA-RD17 promoted colony formation (Additional file 1: Fig. S14F, G). The proliferative activity of keratinocytes was inhibited by down-regulation of miR-632, whereas OA-RD17 significantly promoted keratinocyte proliferation following down-regulation of miR-632 (Additional file 1: Fig. S14H). The expression of Ki67, Cyclin A1, and CDK2 in keratinocytes were inhibited by the down-regulation of miR-632 (Additional file 1: Fig. S14I–K). However, after downregulation of miR-632, OA-RD17 significantly promoted Ki67, CDK2, and Cyclin A1 (Additional file 1: Fig. S14I–K).

### OA-RD17 upregulated miR-632 by activating MAPK pathway via TLR4, and miR-632 promoted keratinocyte proliferation and migration by targeting GSK3β to activate Wnt/β-catenin signaling pathway

**Fig. 7:**
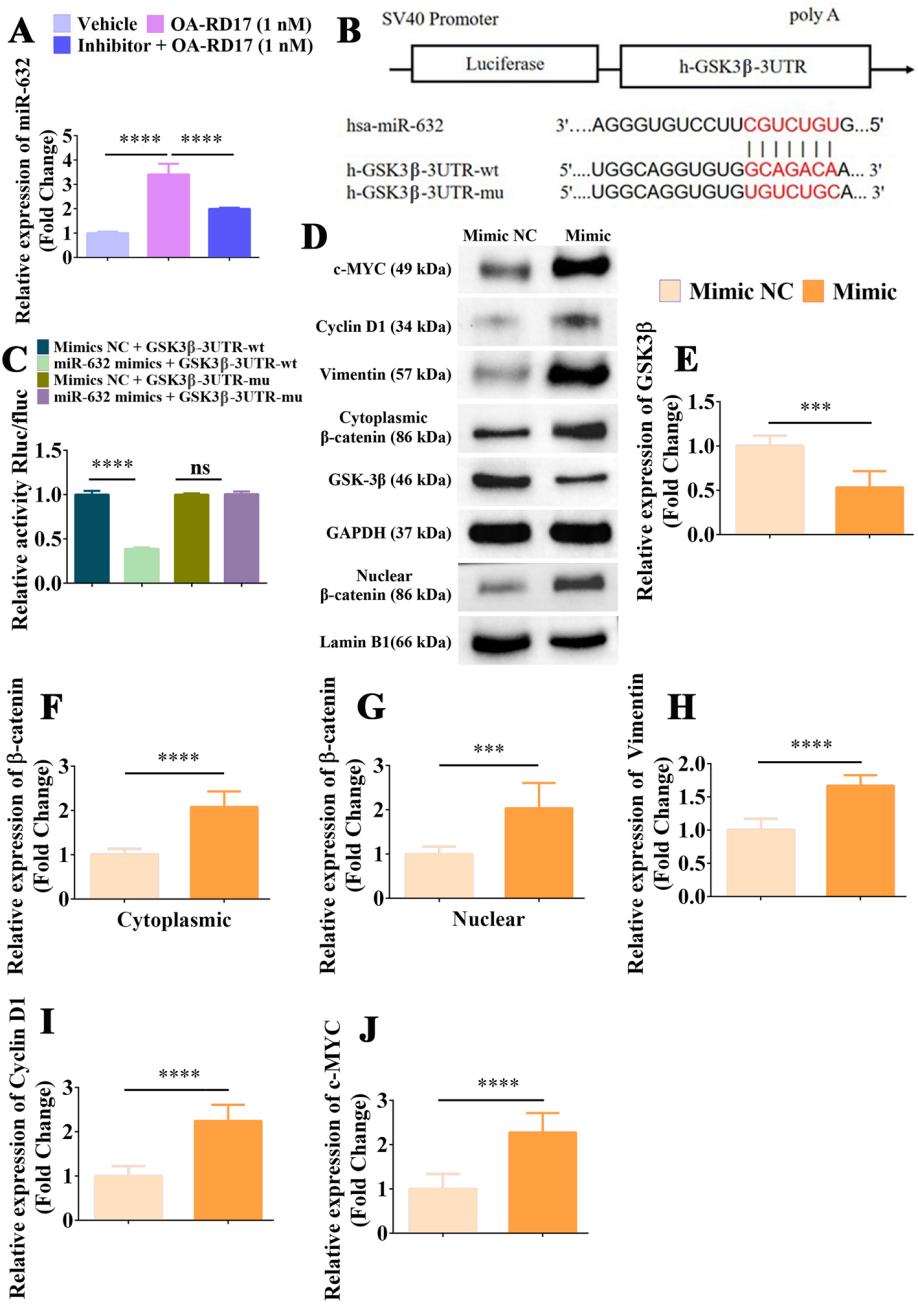
MiR-632 promoted β-catenin expression by targeting GSK3β and activated the Wnt/β-catenin signaling pathway to promote keratinocyte proliferation and migration. **A** Changes in miR-632 expression in OA-RD17-treated keratinocytes after application of MAPK signaling pathway inhibitors for 24 h, detected by RT-qPCR. **B** Prediction of binding sites of miR-632 to GSK3β. **C** Luciferase reporter assay of miR-632 targeting GSK3β. **D** Effects of miR-632 up-regulation on Wnt/β-catenin signaling pathway, detected by western blotting. **E** Up-regulation of miR-632 expression significantly inhibited expression of GSK3β protein. **F**–**J** Up-regulation of miR-632 expression significantly promoted expression of β-catenin, nuclear accumulation of β-catenin, Vimentin, Cyclin D1, and c-MYC proteins. All data are expressed as mean ± SEM from three independent experiments, ns, no significance, ***P < 0.001, and ****P < 0.0001

MiR-632 is essential for keratinocytes proliferation and migration, and OA-RD17 significantly up-regulated miR-632 expression to affect keratinocyte proliferation and migration. However, the regulation of miR-632 by OA-RD17 and the mechanism of miR-632 in regulating keratinocyte functions remain unknown. Therefore, we next investigated the regulation of miR-632 by OA-RD17 and the molecular mechanisms of miR-632 to promote the proliferation and migration on keratinocytes. OA-RD17 activated MAPK signaling pathway and RNA-seq showed that miRNAs were associated with MAPK signaling pathway, thus we hypothesized that OA-RD17 might activate MAPK signaling pathway via TLR4 to upregulate miR-632. Interestingly, both specific MAPK signaling pathway and TLR4 inhibitor significantly suppressed the up-regulation of miR-632 by OA-RD17, demonstrating that OA-RD17 activated the MAPK signaling pathway through the TLR4 to up-regulate miR-632 expression (Fig. 7A, Additional file 1: Fig. S15A).

The miRNAs usually exert biological functions by targeting specific genes [29]. The TargetScan algorithm (https://www.targetscan.org/vert72/) predicted GSK3β to be direct target of miR-632 (Fig. 7B). Moreover, the direct targeting of miR-632 by GSK3β was also verified by the luciferase reporter assay, indicating that miR-632 may exert biological functions by regulating GSK3β (Fig. 7C). GSK3β is a negative regulatory protein for Wnt/β-catenin signaling pathway activation [31]. Here, GSK3β expression was suppressed by overexpression of miR-632 (Additional file 1: Fig. S15B). Notably, overexpression of miR-632 significantly increased the mRNA expression levels of β-catenin, Vimentin, Cyclin D1, and c-MYC (Additional file 1: Fig. S15C–F). Western blotting indicated that overexpression of miR-632 significantly inhibited GSK3β expression, but increased β-catenin, Vimentin, Cyclin D1, and c-MYC expression, as well as nuclear accumulation of β-catenin, indicating that up-regulation of miR-632 expression activated the Wnt/β-catenin signaling pathway (Fig. 7D–J). As shown in Additional file 1: Fig. S15G, H, β-catenin inhibitor significantly suppressed the promoting activity of miR-632 on keratinocyte scratch healing. In addition, the treatment of β-catenin inhibitor significantly inhibited the pro-keratinocyte proliferation ability of miR-632 (Additional file 1: Fig. S15I). These results demonstrated that OA-RD17 upregulated miR-632 expression by activating the MAPK signaling pathway through TLR4, and miR-632 promoted keratinocyte proliferation and migration by targeting GSK3β to activate the Wnt/β-catenin signaling pathway.

### Down-regulation of miR-632 promoted GSK3β expression and inhibited Wnt/β-catenin activation, while OA-RD17 mitigated these inhibitory effects

At the mRNA level, inhibition of miR-632 expression significantly increased GSK3β expression, and decreased the expression levels of β-catenin and Wnt/β-catenin signaling pathway downstream factors Vimentin, Cyclin D1, and c-MYC (Additional file 1: Fig. S16A–E). However, OA-RD17 significantly alleviated the inhibition of Wnt/β-catenin signaling pathway activation by miR-632 inhibitor (Additional file 1: Fig. S16A–E). Next, western blotting was performed to reveal the effects of down-regulation miR-632 and the addition of OA-RD17 after miR-632 down-regulation on the activation of the Wnt/β-catenin signaling pathway (Additional file 1: Fig. S17A). Results showed that down-regulation of miR-632 expression significantly promoted GSK3β protein expression, inhibited β-catenin, Vimentin, Cyclin D1, and c-MYC expression, and inhibited Wnt/β-catenin signaling pathway activation (Additional file 1: Fig. S17B–G). In contrast, OA-RD17 reversed the increase in GSK3β expression and decrease in β-catenin, Vimentin, Cyclin D1, and c-MYC expression caused by the down-regulation of miR-632 (Additional file 1: Fig. S17B–G). These results demonstrated that down-regulation of miR-632 expression significantly inhibited Wnt/β-catenin signaling pathway activation, while OA-RD17 up-regulated miR-632 expression to promote Wnt/β-catenin signaling pathway activation. In summary, our study firstly demonstrated that miR-632 could activate the Wnt/β-catenin signaling pathway and then promote the proliferation and migration of keratinocytes. Moreover, OA-RD17 promoted keratinocyte proliferation, migration and up-regulated miR-632 expression by activating the MAPK pathway via the TLR4, and miR-632 could activate the Wnt/β-catenin signaling pathway to promote keratinocyte proliferation and migration in a positive feedback regulatory manner.

### Up-regulation of miR-632 expression significantly promoted full-thickness skin wound regeneration in rats

**Fig. 8:**
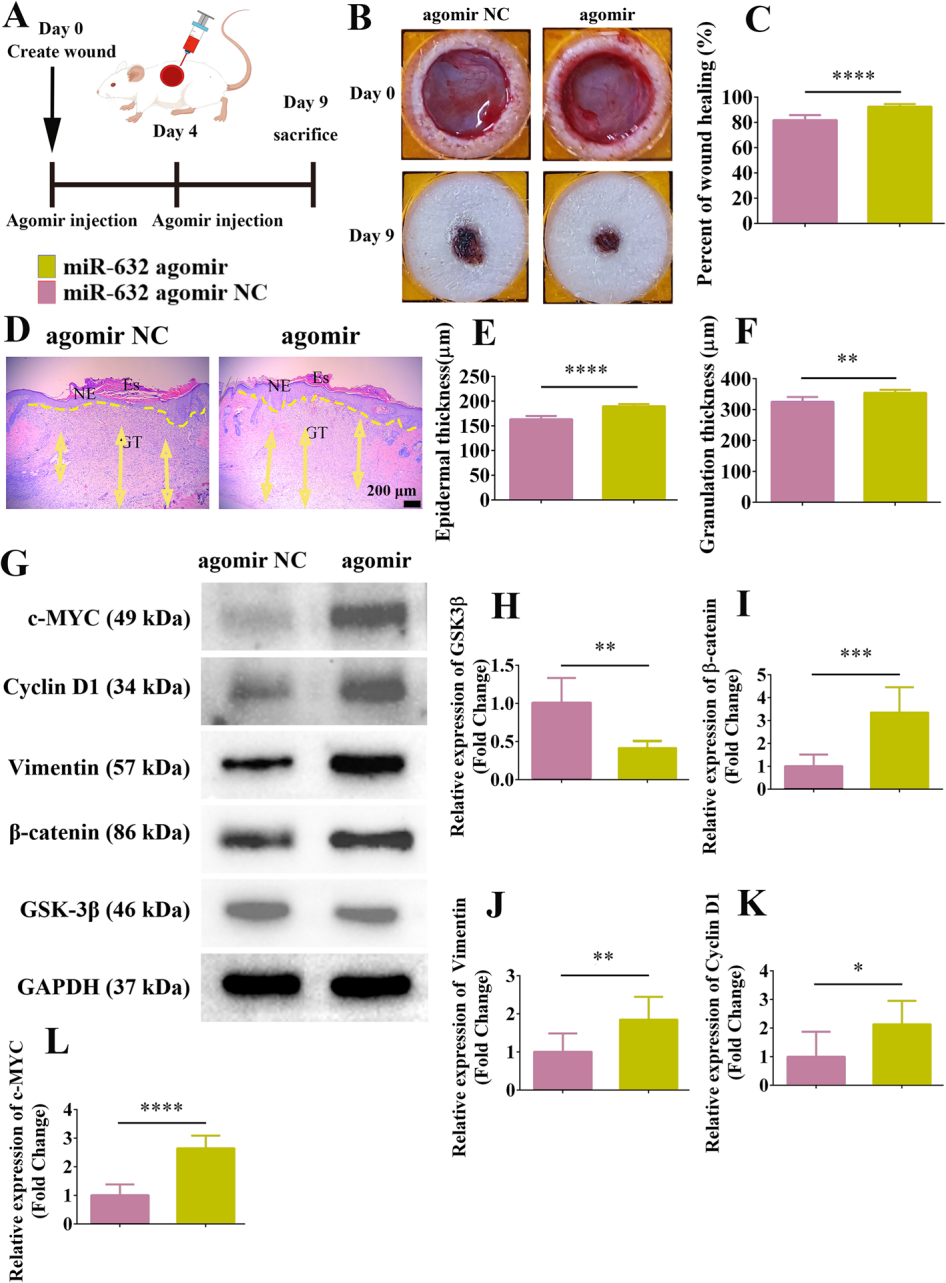
Up-regulation of miR-632 expression activated Wnt/β-catenin signaling pathway to promote skin wound regeneration in rats. **A** Schematic of full-thickness skin wound in SD rats and subcutaneous injection of miR-632 agomir on postoperative days 0 and 4, with subsequent study manipulations. **B**, **C** Representative images of wound repair in SD rats treated with miR-632 agomir for 9 days and quantification of wound repair rate. **D** Representative H&E staining of wound tissue after 9 days of miR-632 agomir treatment in SD rats; Yellow dotted lines represent areas of neoepithelium; Es: eschar; NE: neoepithelium; GT: regenerated granulation tissue; Yellow arrows represent quantified areas of regenerated granulation tissue; scale bar 200 μm. **E**, **F** Quantification of epidermal regeneration thickness and regenerated granulation tissue in wound area. **G** Western blot analysis of Wnt/β-catenin signaling pathway activation in wound region of SD rats after 9 days of miR-632 agomir treatment. **H**–**L** Quantification of GSK3β, β-catenin, Vimentin, Cyclin D1, and c-MYC expression after up-regulation of miR-632. All data are expressed as mean ± SEM from three independent experiments, *P < 0.05, **P < 0.01, ***P < 0.001, and ****P < 0.0001

As miR-632 showed excellent pro-healing potential at the cellular level, we next explored its therapeutic effects on full-thickness skin wounds in Sprague-Dawley (SD) rats (Fig. 8A). Treatment with miR-632 agomir for 9 days showed excellent therapeutic effects on rat skin wounds, with the wound repair rate reaching 92.4%, significantly higher than that of the miR-632 agomir NC group (81.7%) (Fig. 8B, C). After 9 days of miR-632 agomir treatment, the neo-epidermis completely covered the wound area, whereas the miR-632 agomir NC-treated group showed significantly epidermal defects (Fig. 8D). Epidermal thickness and granulation tissue regeneration in SD rats was significantly higher in the miR-632 agomir-treated group than in the miR-632 agomir NC-treated group (Fig. 8E, F). In addition, Ki67 expression in the wound epidermal tissue was 1.89-fold higher after miR-632 agomir treatment than miR-632 agomir NC treatment (Additional file 1: Fig. S18A, B). Western blotting demonstrated that GSK3β expression in the rat skin was significantly inhibited after miR-632 agomir treatment compared to miR-632 agomir NC treatment (Fig. 8G, H). Furthermore, miR-632 agomir treatment significantly promoted the expression of β-catenin, Vimentin, Cyclin D1, and c-MYC, consistent with the up-regulation of miR-632 at the cellular level, suggesting that miR-632 agomir up-regulation of miR-632 activated the Wnt/β-catenin signaling pathway to promote skin wound regeneration in SD rats (Fig. 8I–L).

## Discussion

In the current study, we identified a novel pro-healing peptide OA-RD17, which suppressed inflammation and promoted regeneration of epidermis and granulation, exhibiting excellent therapeutic on skin wounds in mice and diabetic patient ex vivo skin wounds. Mechanistically, OA-RD17 promoted macrophage proliferation and migration by activating the MAPK signaling pathway, inhibited NF-κB signaling pathway and promoted macrophage polarization to the MII phenotype to suppress excessive inflammation. Moreover, OA-RD17 not only inhibited inflammation in keratinocytes, but also promoted keratinocyte proliferation, migration and up-regulated miR-632 expression by activating the MAPK pathway via the TLR4, and miR-632 could activate the Wnt/β-catenin signaling pathway to promote keratinocyte proliferation and migration in a positive feedback regulatory manner, thereby promoting skin wound regeneration. Using OA-RD17 as a molecular probe, our study first evidenced that the ‘TLR4/MAPK’ and ‘Wnt/β-catenin’ signaling pathways are tightly associated with keratinocyte proliferation and migration and promoted skin wound healing. Our research also highlighted the potential of OA-RD17 and miR-632 as promising drug candidates for wound healing, and the crucial role of amphibian-derived peptides in providing novel strategies for skin wound intervention.

The proliferation and migration of macrophages into wounds could not only regulate immune responses, but also secrete cytokines to activate keratinocytes [51]. Moreover, keratinocyte proliferation and migration are essential for re-epithelialization process [26]. Here, OA-RD17 significantly promoted the proliferation and migration of macrophages and keratinocytes to accelerate re-epithelialization and regenerative granulation tissue formation, thus accelerating wound healing in mice (Figs. 1, 2, Additional file 1: Fig. S3). Re-epithelialization is essential for wound healing, and is an important parameter to assess the quality of wound healing [22]. Importantly, compared to other pro-healing peptides, e.g., AH90, tylotoin, RL-QN15, cathelicidin-OA1, OM-LV20, and OA-GP11 dimer [16, 17, 37, 52–54], OA-RD17 exhibited potent pro-healing activity on diabetic patient ex vivo skin wounds, demonstrating its better clinical applications (Fig. 3B–F). Altogether, the newly identified peptide OA-RD17 showed excellent therapeutic effects on skin wound rehabilitation.

An excessive inflammation can prevent proper wound repair and cause chronic non-healing wounds [3]. Our results showed that OA-RD17 significantly inhibited the expression of inflammatory factors in both mouse full-thickness skin wounds and diabetic patient ex vivo skin wounds, thereby accelerating skin wound healing (Fig. 1I–K, 3I, J, Additional file 1: Fig. S7). Moreover, in the early stages of wound healing, the secretion of pro-inflammatory cytokines promotes cytokine secretion and clearance of necrotic tissue, however, a persistent inflammatory response will severely impede wound healing [4, 55]. OA-RD17 also regulated inflammatory response intensity at different stages of skin wound healing (Fig. 2I, J, Additional file 1: Fig. S7), not only defending against pathogens and clearing necrotic tissue but also suppressing the excessive inflammation and accelerating re-epithelialization to promote wound healing.

Next, we investigated the mechanism of OA-RD17 on macrophages and keratinocytes to promote wound healing. The MAPK signaling pathway is essential in the proliferation and migration of macrophages and keratinocytes [56, 57]. Our results showed that OA-RD17 promoted macrophage proliferation and migration via activation of the MAPK signaling pathway (Fig. 4I, J, Additional file 1: Fig. S9). Macrophages play important roles in the regulation of inflammatory response [24, 58]. Previous studies have also shown that the Ot-WHP and OA-GP11 dimer peptide regulates the NF-κB signaling pathway in macrophages to modulate inflammation and accelerate wound healing [17, 46]. In our study, OA-RD17 inhibited the NF-κB signaling pathway in macrophages and significantly promoted the polarization of MI macrophages to MII macrophages, thereby suppressing the inflammatory response. MI macrophages have pro-inflammatory activity while MII macrophages have anti-inflammatory activity, and the transition of macrophages from MI to MII phenotype can suppresses excessive inflammation to promote wound healing [47, 59].

Extracellular molecules usually exert their biological functions by interacting with transmembrane receptors or entering the cell directly [49]. The results of molecular docking and co-localization confirmed that OA-RD17 could bind to TLR4 (Fig. 6A, B). TLR4 can regulate inflammation by NF-κB and MAPK, and TLR4-deficient mice significantly impair skin wound healing [27]. However, whether ‘TLR4/MAPK’ signaling pathway affects keratinocyte proliferation and migration has not yet been reported. Interestingly, TLR4 inhibitor significantly suppressed the promotion of keratinocyte proliferation and migration and MAPK signaling pathway activation by OA-RD17, suggesting that OA-RD17 promotes keratinocyte proliferation and migration by activation of the MAPK signaling pathway via TLR4 (Fig. 6). Thus, using OA-RD17 as a molecular probe, these findings provide the first solid evidence that the ‘TLR4/MAPK’ signaling pathway regulates keratinocyte proliferation and migration.

MiRNAs are involved in nearly all stages of wound healing and play important roles in the process of wound repair [60]. Therefore, miRNAs are recognized as important emerging drugs or drug targets for skin wound healing strategies [40, 61]. In the present study, OA-RD17 upregulated miR-632 expression through activation of the MAPK signaling pathway by TLR4, suggesting that it may have an important role in the wound repair. MiR-632 is known to exhibit pro-proliferative and migratory effects on hepatocellular and laryngeal carcinoma cells, but its biological function on keratinocytes remains unclear [50, 62]. Our results showed that miR-632 had important effects on keratinocyte proliferation and migration, and OA-RD17 could exert biological functions on keratinocytes by regulating the expression of miR-632 (Additional file 1: Fig. S13, S14). MiR-632 significantly promoted the proliferation and migration of keratinocytes, suggesting that it may have important therapeutic effects on skin wound repair. Therefore, investigation of the mechanism of miR-632 on keratinocytes and its therapeutic potential for wound healing may provide new insight into skin wound healing.

MiRNAs regulate biological processes by targeting their target genes. Here, luciferase reporter analysis demonstrated the targeting relationship between miR-632 and GSK3β (Fig. 7C). GSK3β is a negative regulator of the Wnt/β-catenin signaling pathway [31]. The Wnt/β-catenin signaling pathway plays an important role in skin wound repair by regulating cell proliferation and differentiation [63, 64]. However, it remains controversial whether activation of Wnt/β-catenin signaling promotes skin wound healing by regulating keratinocytes [35, 65]. Our results demonstrated that miR-632 regulated keratinocyte proliferation and migration by modulating Wnt/β-catenin signaling, and OA-RD17 could regulate miR-632 expression to affect Wnt/β-catenin signaling activation, thus promoting keratinocyte proliferation and migration (Fig. 7, S15B–F, S16, S17). More importantly, the up-regulation of miR-632 significantly activated Wnt/β-catenin signaling, and promoted re-epithelialization and granulation tissue regeneration, thereby accelerating the full-thickness skin wounds healing in SD rats (Fig. 8). Taken together, our study firstly demonstrated that miR-632 activated Wnt/β-catenin signaling pathway to promoted keratinocyte proliferation and migration, and re-epithelialization, thus accelerating skin wound healing.

Poor pharmacokinetics greatly limited the clinical utility of drugs. Here, although the peptide was applied topically to the skin surface for cutaneous wound treatment, the pharmacokinetics of the peptide should still be examined. Furthermore, peptides are susceptible to modification by endogenous (e.g., tyrosinase, metalloproteinases, elastase) and exogenous (e.g., produced by colonizing microorganisms) enzymes at the wound site, resulting in their inactivation [66, 67]. Therefore, exploring the drug metabolism and enhancing enzyme tolerance of OA-RD17 is crucial for the future development of OA-RD17-based drugs for skin wound treatment. To improve the stability of peptides, our research teams have dedicated to combining highly active pro-healing peptides with nanomaterials and hydrogels, which significantly enhance the stability and prolong the duration of effects of peptides and boost the clinical application prospects of peptides [5–7]. In addition, the limitations of our study were that we should further investigate the molecular mechanism of up-regulation miR-632 expression induced by OA-RD17 through TLR4/MAPK molecular axis, and the effect of knockdown of miR-632 expression on wound healing and Wnt/β-catenin pathway in SD rats.

## Conclusions

In summary, we identified a novel amphibian-derived peptide (OA-RD17) with excellent pro-healing activity. Furthermore, using OA-RD17 as a molecular probe, we firstly demonstrated the activation of TLR4/MAPK molecular axis and Wnt/β-catenin signaling pathway promoted proliferation and migration of keratinocytes, thereby accelerating skin wounds healing. Our results identified the TLR4 and Wnt/β-catenin signaling pathway as potential therapeutic targets for skin regeneration and miR-632 as a promising nucleic acid drug for skin wound regeneration.

## Supplementary Information

**Additional file 1:** **Table S1.** Acute toxicity test in mice. **Table S2.** Primers used for qRT-PCR. **Table S3.** Characteristics of diabetic patients. **Figure S1.** Structural features of OA-RD17. **Figure S2.** Hemolytic activity of OA-RD17. Hemolytic activity of OA-RD17 on mouse erythrocytes. **Figure S3.** Pro-healing ability of OA-RD17 at cellular level. **Figure S4.** OA-RD17 promoted migration and proliferation of primary mouse keratinocytes and macrophages. **Figure S5.** OA-RD17 promoted macrophage polarization from MI to MII phenotype. **Figure S6.** Hematoxylin and eosin (H&E) staining of deep second-degree burn in mice. H&E staining indicated pathological changes in normal skin and burned skin (1 h after burn) of mice, scale bar 200 μm. H&E staining of mouse skin burned for 1 h was obtained from every mouse burn model. **Figure S7.** OA-RD17 significantly suppressed the expression of IL-6 and TNF-α. **Figure S8.** Polarization of macrophages in wound area of mice with deep second-degree burns on days 8 and 14. **Figure S9.** MAPK signaling pathway inhibitors significantly inhibited proliferation- and migration-promoting activity of OA-RD17 on macrophages. **Figure S10.** RNA sequencing of mRNA levels of differentially expressed genesinvolved in biological processes, components, and signaling pathways following OA-RD17 treatment in keratinocytes. **Figure S11.** RNA sequencing of differentially expressed miRNAs involved in biological components, processes, and signaling pathways following OA-RD17 treatment in keratinocytes. **Figure S12.** Molecular docking of TLR4 and OA-RD17. **Figure S13.** OA-RD17 significantly up-regulated miR-632, which significantly promoted keratinocyte proliferation and migration. **Figure S14.** Inhibition of miR-632 expression significantly inhibited keratinocyte proliferation and migration, while OA-RD17 restored effect of miR-632 down-regulation on keratinocyte proliferation and migration. **Figure S15.** Up-regulation of miR-632 expression significantly promoted Wnt/β-catenin signaling pathway activation. **Figure S16.** Expression of GSK3β, β-catenin, c-MYC, Cyclin D1, and Vimentin mRNA after down-regulation of miR-632. **Figure S17.** Down-regulation of miR-632 significantly inhibited Wnt/β-catenin signaling pathway activation. **Figure S18.** MiR-632 significantly promoted the expression of Ki67 in epidermis of wound area.

## Data Availability

The datasets used and/or analyzed in the current study are available from the corresponding author upon reasonable request.

## Abbreviations

rh-bFGF: Recombinant human basic fibroblast growth factor
FBS: Fetal bovine serum
DMEM: Dulbecco’s Modified Eagle Medium
PBS: Phosphate-buffered saline
HaCaT: Human immortalized keratinocyte cell line
LPS: Lipopolysaccharide
H&E: Hematoxylin and eosin
TNF-α: Tumor necrosis factor alpha
IL-1β: Interleukin-1β
IL-6: Interleukin-6
INOS: Inducible nitric oxide synthase
ARG: Arginase
CAMs: Cell adhesion molecules
DAPI: 4′,6-Diamidino-2-phenylindole
HRP: Horseradish peroxidase
ELISA: Enzyme-linked immunosorbent assay
DEGs: Differentially expressed genes
GO: Gene Ontology
KEGG: Kyoto Encyclopedia of Genes and Genomes
MAPK: Mitogen-activated protein kinase
TLR4: Toll-like receptor 4
NF-κB: Nuclear factor‐kappa B
