# Correction: A frog peptide provides new strategies for the intervention against skin wound healing

**DOI:** 10.1186/s11658-026-00877-0

**Published:** 2026-03-03

**Authors:** Chao Li, Zhe Fu, Tao Jin, Yixiang Liu, Naixin Liu, Saige Yin, Zhuo Wang, Yubing Huang, Yinglei Wang, Yingxuan Zhang, Jiayi Li, Yutong Wu, Li He, Jing Tang, Ying Wang, Xinwang Yang

**Affiliations:** 1 Department of Anatomy and Histology & Embryology, Faculty of Basic Medical Science, Kunming Medical University, Yunnan, 650500 Kunming China; 2 Key Laboratory of Chemistry in Ethnic Medicinal Resources & Key Laboratory of Natural Products Synthetic Biology of Ethnic Medicinal Endophytes, State Ethnic Affairs Commission & Ministry of Education, School of Ethnic Medicine, Yunnan Minzu University, Yunnan, 650504 Kunming China; 3 Department of Biochemistry and Molecular Biology, Faculty of Basic Medical Science, Kunming Medical University, Yunnan, 650500 Kunming China; 4 Department of Dermatology, First Affiliated Hospital of Kunming Medical University, Yunnan, 650032 Kunming China; 5 Department of Orthopedics, 920th Hospital of Joint Logistics Support Force of PLA, Yunnan, 650032 Kunming China

**Correction: Cellular & Molecular Biology Letters (2023) 28:61**

10.1186/s11658-023-00468-3

In this article [1], the wrong figure appeared as Figs. 1, 3 and 5, S3, S5, S8, and S14; the correct figures should have appeared as shown below.

**Correct** **Figure 1:**

**Fig. 1:**
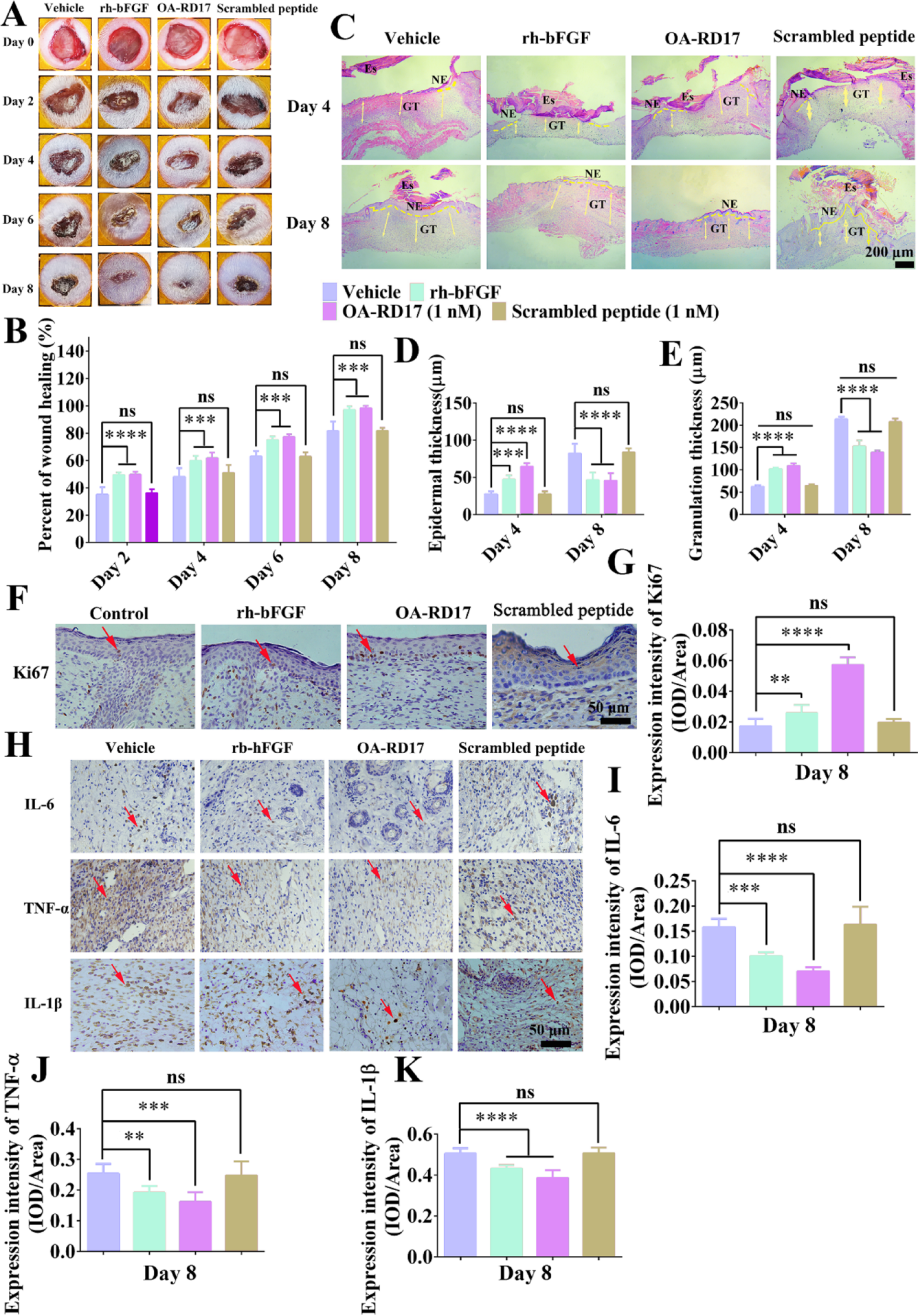
OA-RD17 inhibited inflammation, promoted re-epithelialization and granulation regeneration, and accelerated full-thickness wound healing in mice. **A** Representative images of full-thickness wound healing in mice after local application of PBS, rh-bFGF (100 ng/mL), OA-RD17 (1 nM), or scrambled peptide (1 nM) on postoperative days 0, 2, 4, 6, and 8. **B** Quantification of OA-RD17 on full-thickness wound regeneration in mice on postoperative days 2, 4, 6, and 8. **C** Representative H&E staining of wound tissue in mice on postoperative days 4 and 8. Yellow dotted lines represent areas of neoepithelium; Es: eschar; NE: neoepithelium; GT: regenerated granulation tissue; Yellow arrows represent quantified areas of regenerated granulation tissue; scale bar 200 μm. **D, E** Quantification of neo-epidermal and regranulation tissue thickness on postoperative days 4 and 8. **F** Representative images of immunohistochemical staining of epidermal cell proliferation factor Ki67 expression in epidermal region of wound tissues on postoperative day 8; red arrows indicate positive staining, scale bar 50 μm. **G** Quantitative expression of epidermal cell proliferation factor Ki67; positive expression is defined as intensity of positive staining per unit area. **H** Representative immunohistochemical images of inflammatory factors IL-6, TNF-α, and IL-1β in wound area of mice on postoperative day 8; red arrows indicate positive staining, scale bar 50 μm. **I-K** Quantification of IL-6, TNF-α, and IL-1β expression in wound area on day 8 after treatment; positive expression is defined as intensity of positive staining per unit area. All data are expressed as mean ± SEM from three independent experimentsperformed in quintuplicate; ns, no significance; **P < 0.01, ***P < 0.001, and ****P < 0.0001 indicate statistically significant difference compared to vehicle

**Correct** **Figure 3:**

**Fig. 3:**
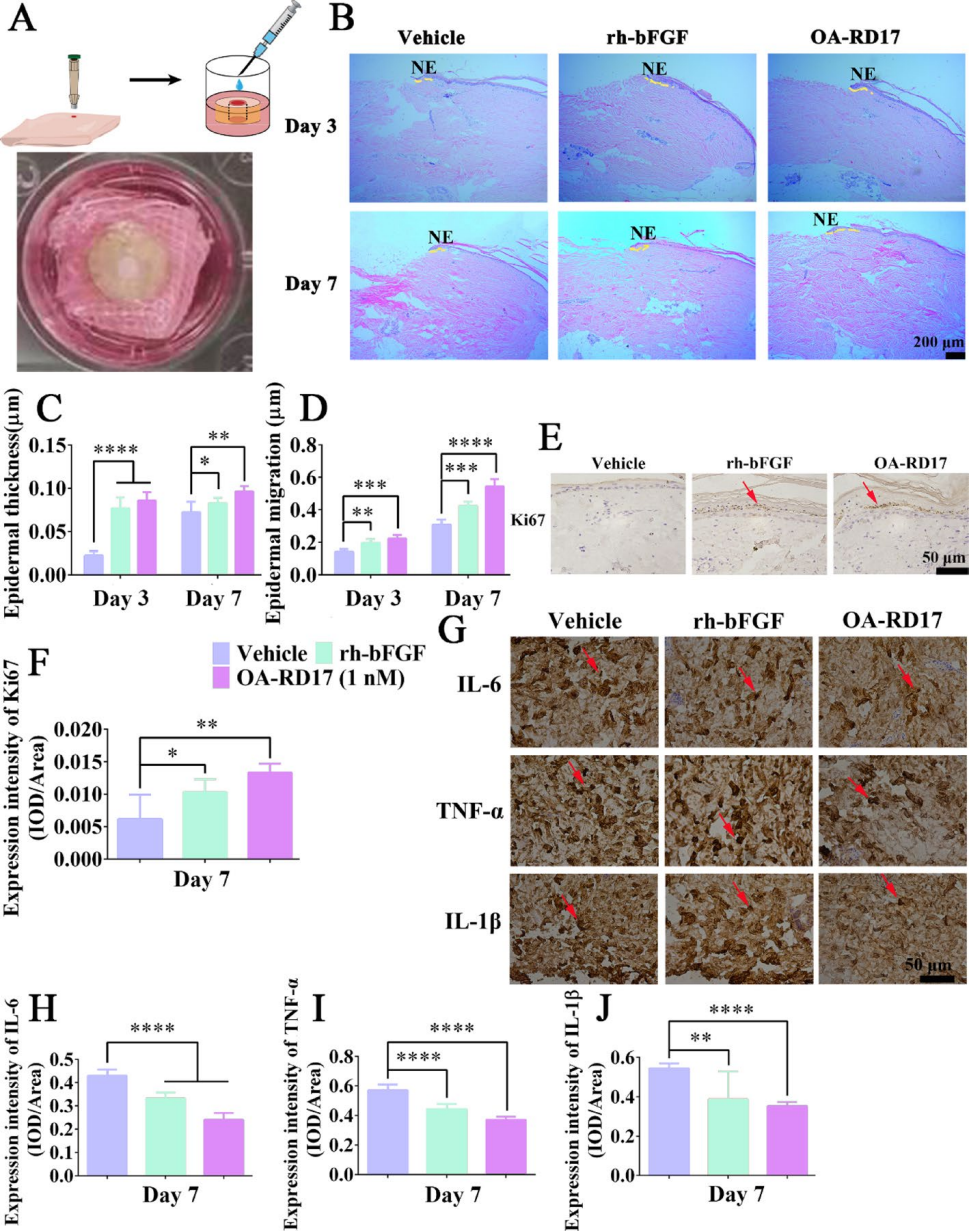
OA-RD17 significantly decreased inflammation and promoted epidermal regeneration in diabetic patient ex vivo skin wounds. **A** Illustration of diabetic patient ex vivo skin wound model. **B** Representativeimages of H&E staining of diabetic patient ex vivo skin wound tissues on days 3 and 7. Yellow dotted lines represent areas of neoepithelium; NE: neoplastic epithelium; scale bar 200 μm. **C** Thickness variations in neodermis in wound tissue on days 3 and 7 of OA-RD17 treatment. **D** Epidermal migration length variation in neodermis in wound tissue on days 3 and 7 of OA-RD17 treatment. **E** Representative images of immunohistochemical staining for epidermal Ki67 expression after one week of OA-RD17 treatment; positive staining is indicated by red arrows, scale bar 50 μm. **F** Quantification of positive-staining intensity of Ki67 expression in epidermis. **G** Representative images of immunohistochemical staining for inflammatory factors IL-6, TNF-α, and IL-1β in wound area on day 7 of OA-RD17 treatment; positive staining is indicated by red arrows, scale bar 50 μm. **H-J** Quantification of intensity of IL-6, TNF-α, and IL-1β expression in wound area. All data are expressed as mean ± SEM from three independent experiments performed in triplicate. *P < 0.05, **P < 0.01, ***P < 0.001, and ****P < 0.0001 indicate statistically significant difference compared to vehicle

**Correct** **Figure 5:**

**Fig. 5:**
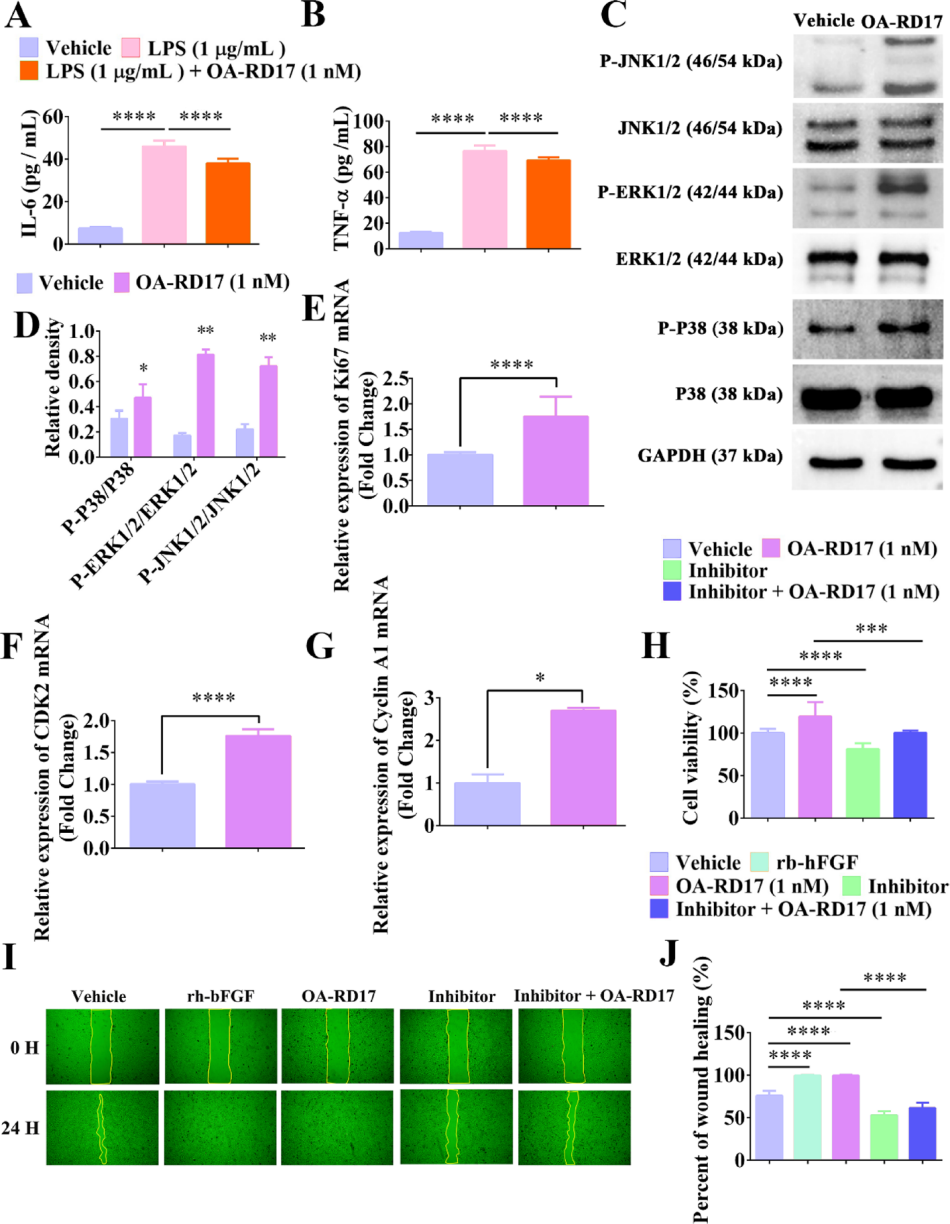
OA-RD17 suppressed inflammatory factor expression and activated the MAPK signaling pathway to promote keratinocyte proliferation and migration. **A, B** ELISA analysis of effects of OA-RD17 on inflammatory factors (IL-6 and TNF-α) release in keratinocytes after LPS stimulation. **C** Representative image of MAPK signalling pathway activation in keratinocytes after 24 h of OA-RD17 treatment, determined by western blotting. **D** Quantification of P38, ERK, and JNK protein phosphorylation. **E–G** RT-qPCR analysis of Ki67, CDK2, and CyclinA1 mRNA expression in OA-RD17-treated keratinocytes. **H** Changes in pro-proliferation activity of OA-RD17 in keratinocytes after application of MAPK signaling pathway inhibitors. I Representative images ofeffects of OA-RD17 on keratinocyte scratch repair after application of MAPK signaling pathway inhibitors. **J** Quantification of effects of OA-RD17 on keratinocyte scratch repair rate after application of MAPK signalling pathway inhibitors. All data are expressed as mean ± SEM from three independent experiments, *P 0.05, **P < 0.01, ***P < 0.001, and ****P < 0.0001

**Correct Figure S3**:

**Fig. S3:**
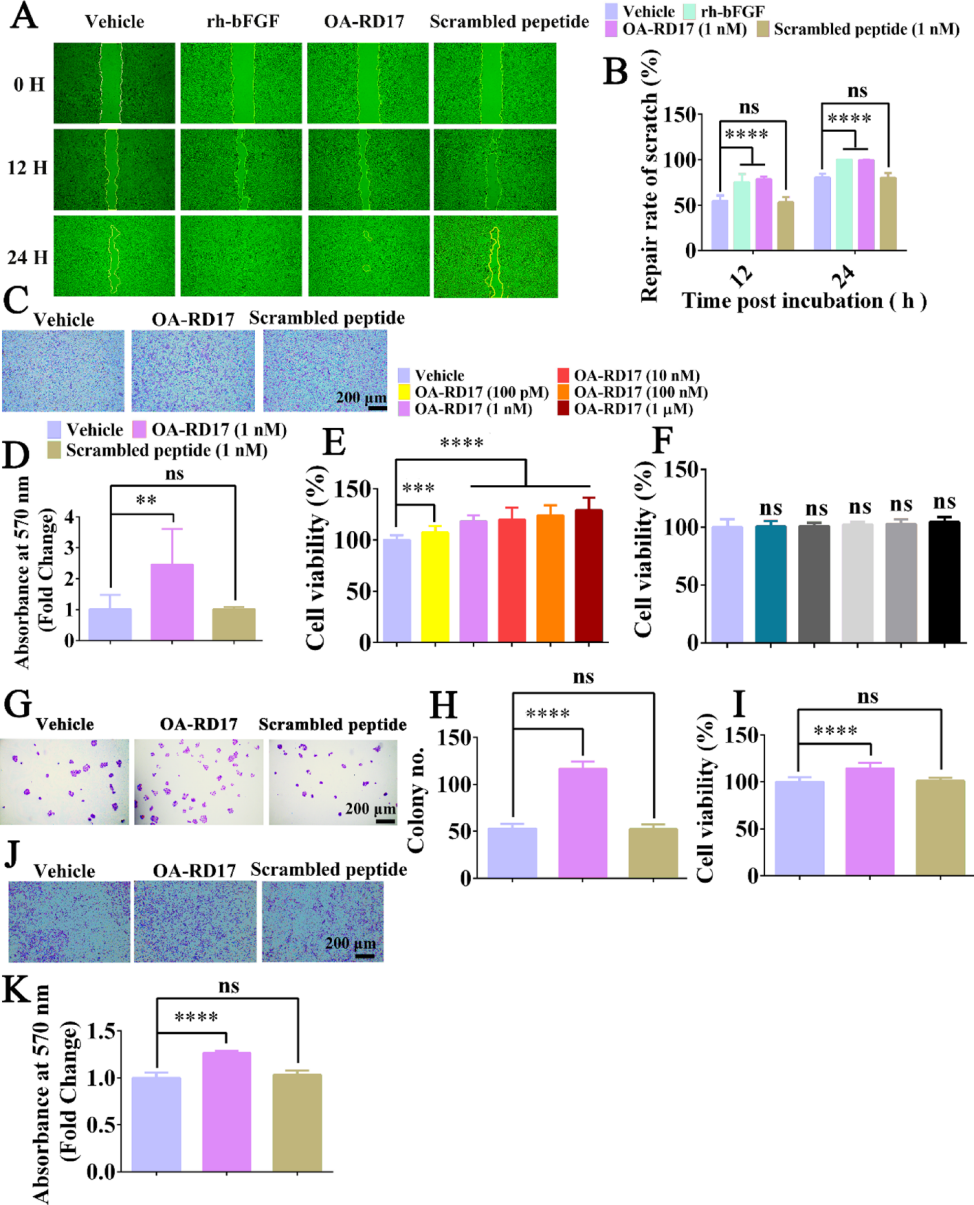
Pro-healing ability of OA-RD17 at cellular level

**Correct Figure S5**:

**Fig. S5:**
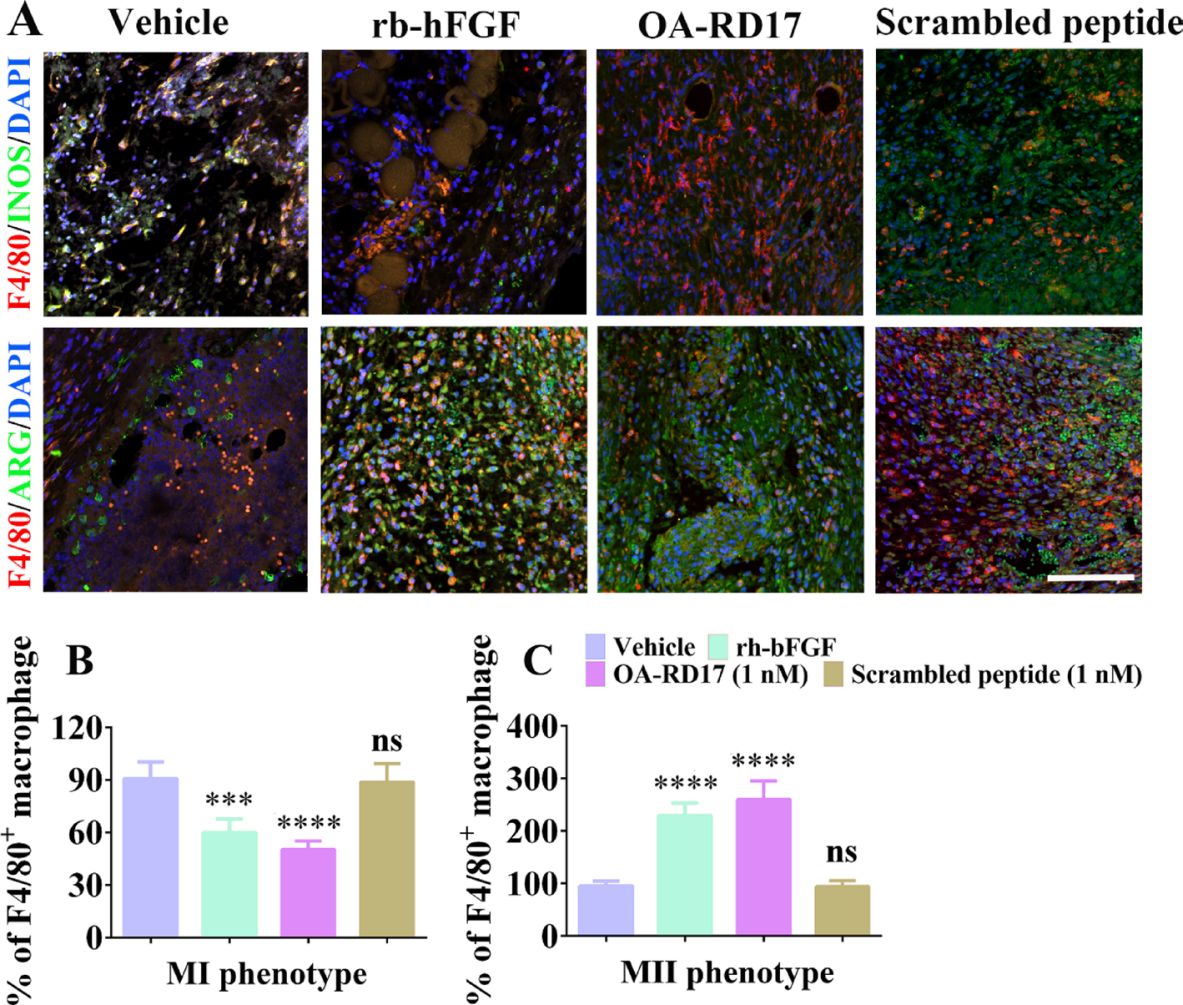
OA-RD17 promoted macrophage polarization from MI to MII phenotype

**Correct Figure S8**:

**Fig. S8:**
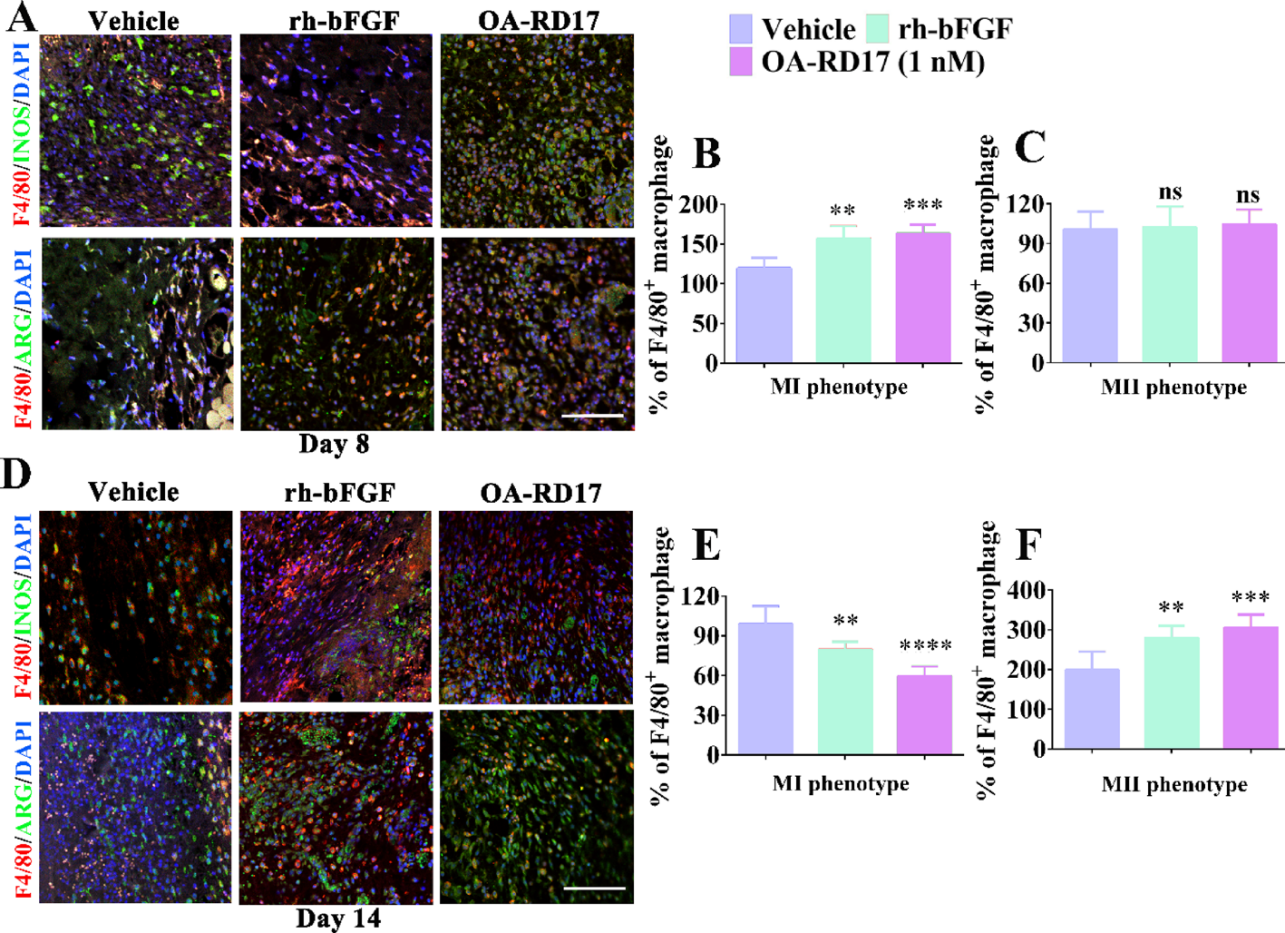
Polarization of macrophages in wound area of mice with deep second-degree burns on days 8 and 14

**Correct Figure S14**:

**Fig. S14:**
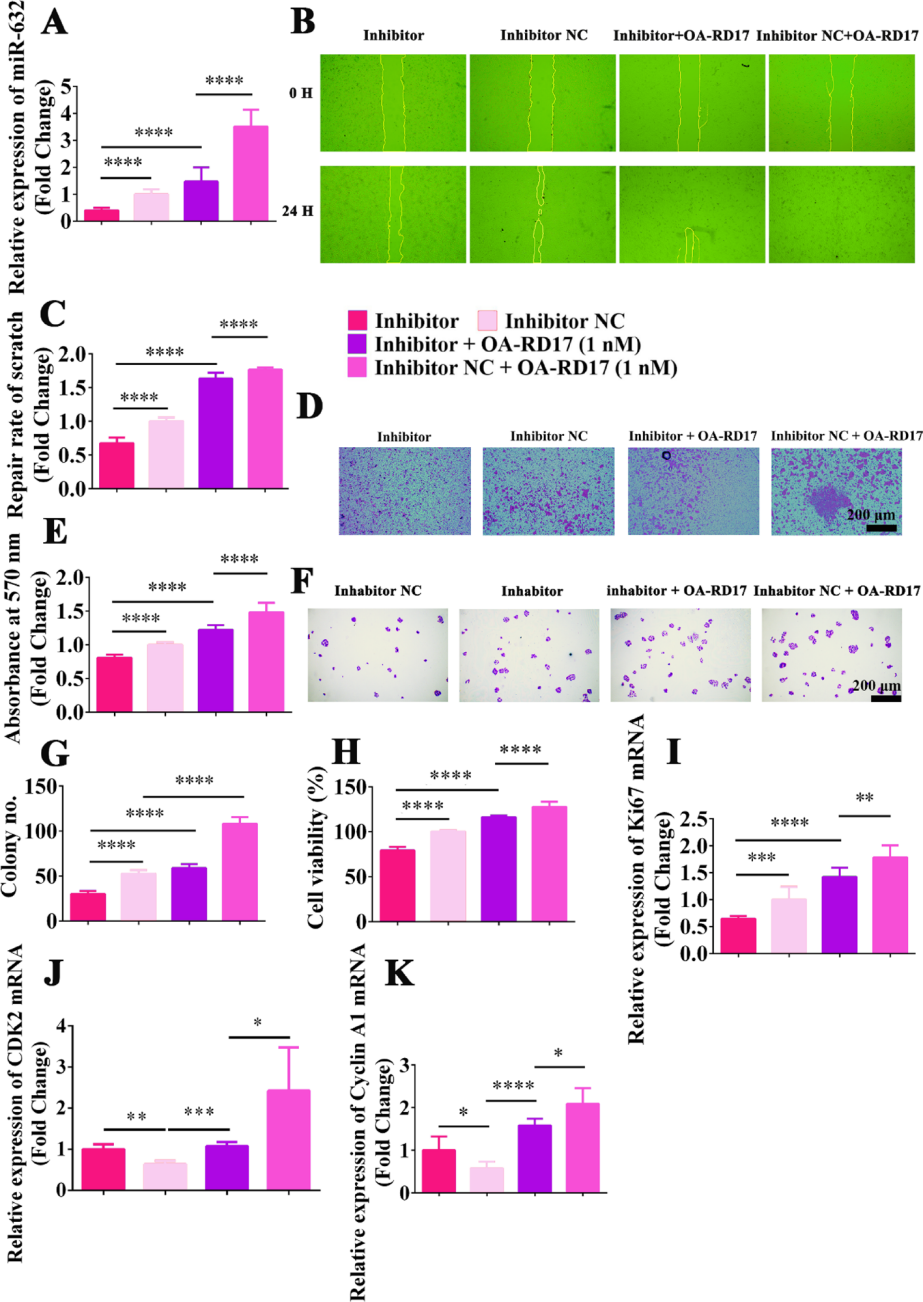
Inhibition of miR-632 expression significantly inhibited keratinocyte proliferation and migration, while OA-RD17 restored effect of miR-632 down-regulation on keratinocyte proliferation and migration
