# Supplementary material for: A frog peptide provides new strategies for the intervention against skin wound healing
Source: Cell Mol Biol Lett. 2023 Jul 28;28:61. doi: 10.1186/s11658-023-00468-3 (PMC10375744; doi:10.1186/s11658-023-00468-3)
Supplement: Supplementary file 1 — Additional file 1: Table S1. Acute toxicity test in mice. Table S2. Primers used for qRT-PCR. Table S3. Characteristics of diabetic patients. Figure S1. Structural features of OA-RD17. Figure S2. Hemolytic activity of OA-RD17. Hemolytic activity of OA-RD17 on mouse erythrocytes. Figure S3. Pro-healing ability of OA-RD17 at cellular level. Figure S4. OA-RD17 promoted migration and proliferation of primary mouse keratinocytes and macrophages. Figure S5. OA-RD17 promoted macrophage polarization from MI to MII phenotype. Figure S6. Hematoxylin and eosin (H&E) staining of deep second-degree burn in mice. H&E staining indicated pathological changes in normal skin and burned skin (1 h after burn) of mice, scale bar 200 μm. H&E staining of mouse skin burned for 1 h was obtained from every mouse burn model. Figure S7. OA-RD17 significantly suppressed the expression of IL-6 and TNF-α. Figure S8. Polarization of macrophages in wound area of mice with deep second-degree burns on days 8 and 14. Figure S9. MAPK signaling pathway inhibitors significantly inhibited proliferation- and migration-promoting activity of OA-RD17 on macrophages. Figure S10. RNA sequencing of mRNA levels of differentially expressed genesinvolved in biological processes, components, and signaling pathways following OA-RD17 treatment in keratinocytes. Figure S11. RNA sequencing of differentially expressed miRNAs involved in biological components, processes, and signaling pathways following OA-RD17 treatment in keratinocytes. Figure S12. Molecular docking of TLR4 and OA-RD17. Figure S13. OA-RD17 significantly up-regulated miR-632, which significantly promoted keratinocyte proliferation and migration. Figure S14. Inhibition of miR-632 expression significantly inhibited keratinocyte proliferation and migration, while OA-RD17 restored effect of miR-632 down-regulation on keratinocyte proliferation and migration. Figure S15. Up-regulation of miR-632 expression significantly promoted Wnt/β-catenin signaling pathwa [file 11658_2023_468_MOESM1_ESM.docx]

**Additional file**

| Group | Female  (number) | | Male  (number) | Death | | Original weight (g) | | Weight change (g) |
| --- | --- | --- | --- | --- | --- | --- | --- | --- |
| PBS | | 3 | 3 | | 0 | | 21.32, 25.68, 27.31,  22.31,26.61, 22.57 | +0.21, +0.14, +0.36,  +0.12, +0.37, +0.28 |
| OA-RD17 (0.1µg/ kg) | | 3 | 3 | | 0 | | 21.94, 23.34, 23.69,  24.57, 23.65, 24.35 | +0.19, +0.17, +0.27,  +0.27, +0.44, +0.33 |
| OA-RD17 (1µg/ kg) | | 3 | 3 | | 0 | | 22.52, 27.31, 25.38,  23.39, 27.38, 25.38 | +0.27, +0.22, +0.16,  +0.32, +0.26, +0.27 |
| OA-RD17 (10µg/ kg) | | 3 | 3 | | 0 | | 24.09, 22.37, 26.64,  25.71, 25.28, 26.42 | +0.11, +0.27, +0.22,  +0.25, +0.49, +0.31 |
| OA-RD17 (100µg/kg) | | 3 | 3 | | 0 | | 25.57, 28.18, 28.21,  23.75, 24.96, 27.62 | +0.14, +0.31, +0.19,  +0.22, +0.36, +0.25 |
| OA-RD17 (1 mg/kg ) | | 3 | 3 | | 0 | | 23.62, 26.28, 25.42,  22.89, 26.39, 25.47 | +0.23, +0.34, +0.41,  +0.12, +0.37, +0.28 |

**Table S1. Acute toxicity test in mice.**

**Table S2. Primers used for qRT-PCR.**

| Gene | Forward | Reverse |
| --- | --- | --- |
| IL-6 | 5’-CAA GAA AGA CAA AGC CAG AGT CCT T-3’ | 5’-TGG ATG GTC TTG GTC CTT AGC C-3’ |
| TNF-α | 5′-ACA AGC CTG TAG CCC ACG-3′ | 5′-TCC AAA GTA GAC CTG CCC-3′ |
| IL-1β | 5’-TGC AGA GTT CCC CAA CTG GTA CAT C-3′ | 5’-GTG CTG CCT AAT GTC CCC TTG AAT C-3′ |
| β-actin | 5’-AGT GTG ACG TTG ACA TCC GTA AAG A-3′ | 5’-GGA CAG TGA GGC CAG GAT GG-3′ |
| Ki67 | 5′-GGA TCG TCC CAG TGG AAG AG-3′ | 5′-CAA ACA AGC AGG TGC TGA GG-3′ |
| Cyclin A1 | 5′-ATA ACG ACG GGA AGA GCG GG-3′ | 5′-CTC CAT CCC AAG TGA CGA GC-3′ |
| miR-632 | 5’-GACGGGAGGCGGAGCGGGGA-3′ |  |
| U6 | 5′-ATTGGAACGATACAGAGAAGATT-3′ | 5′-GGAACGCTTCACGAATTTG-3′ |
| GSK-3β | 5 - CTG TTC CGA AGT TTA GCC TAT AT -3 | 5- ACA AGA GGT TCT GCG GTT TA -3 |
| β-catenin | 5-CACAAGCAGAGTGCTGAAGGTGC-3 | 5-AAGGAGGCCTTCCATCCCTTC-3 |
| Vimentin | 5’-CCGAAAACACCCTGCAATCTTTC-3’ | 5’-CACATCGATTTGGACATGCTGT-3’ |
| Cyclin D1 | 5-GTGGCCTCTAAGATGAAGGAG-3 | 5-GAACTTCACATCTGTGGCACAG-3 |
| c-MYC | 5'‑AAAGGCCCCCAAGGTAGTTA‑3' | 5'‑GCACAAGAGTTCCGTAGCTG‑3' |
| GAPDH | 5’-TTG TAA CCA ACT GGG ACG ATA TGG-3’ | 5’-GAT CTT GAT CTT CAT GGT GCT AGG-3’ |

**Table S3. Characteristics of diabetic patients.**

| Patient | Age (years) | Sex | Location |
| --- | --- | --- | --- |
| Patient 1  Patient 2  Patient 3 | 62  49  54 | Female  Female  Male | Left second toe  Right leg  Right first toe |


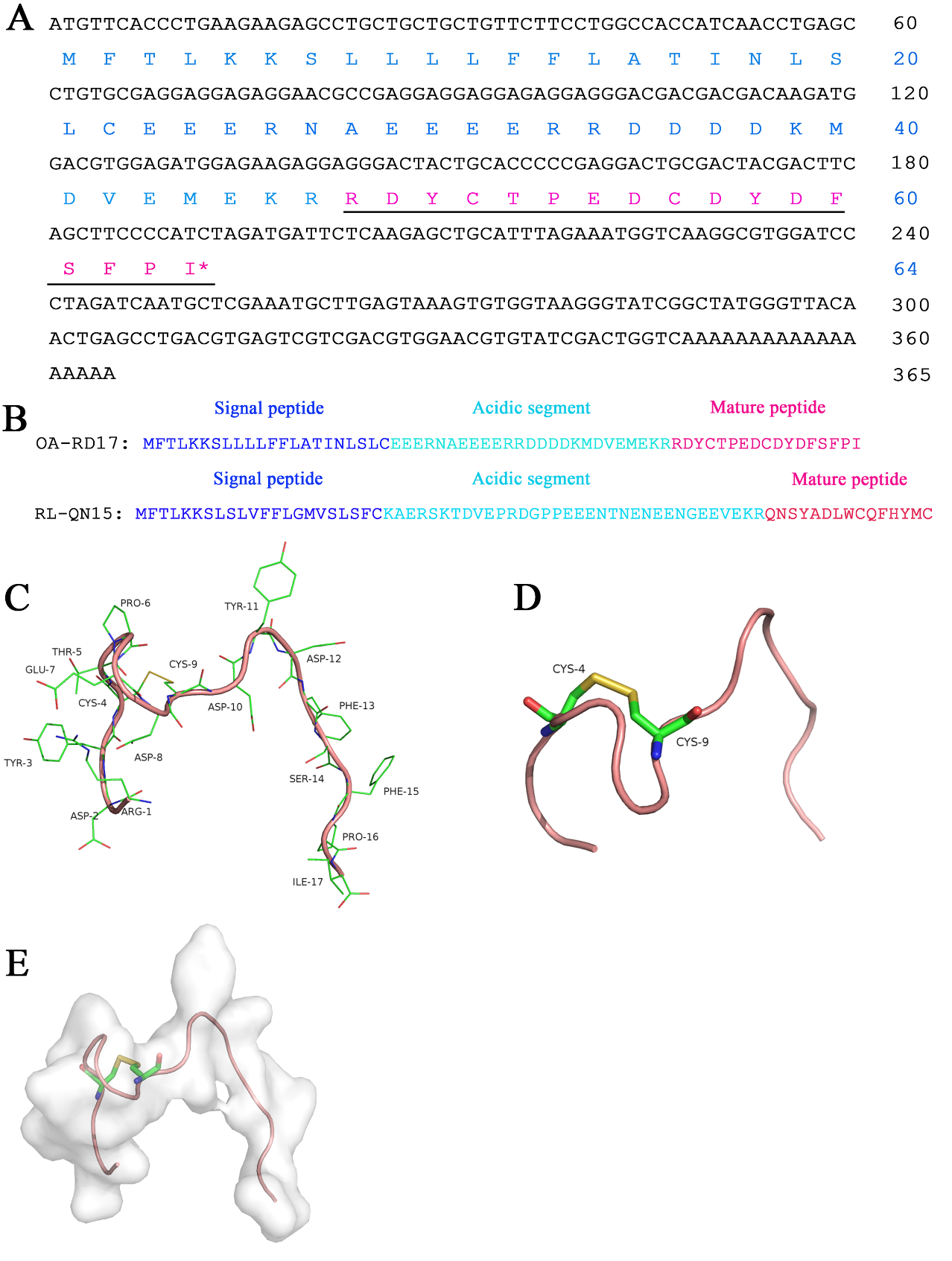


**Figure S1. Structural features of OA-RD17.**

A. cDNA sequence (363 bp) encoded a precursor peptide consisting of 64 amino acid residues, with red amino acid sequence showing mature peptide of OA-RD17.

B. Sequence alignment of proprecursor peptides of OA-RD17 and RL-QN15. Blue sequence is signal peptide of OA-RD17, green sequence is acidic segment of OA-RD17, and red sequence is mature peptide sequence.

C. Prediction of advanced structure of OA-RD17 and amino acid residues.

D. Advanced structure of OA-RD17 predicted by PEP-FOLD3. Blue: nitrogen atom; red: oxygen atom; yellow: sulfur atom; light red: disulfide bridge.

E. Spatial structure of OA-RD17. White part represents peptide surface.


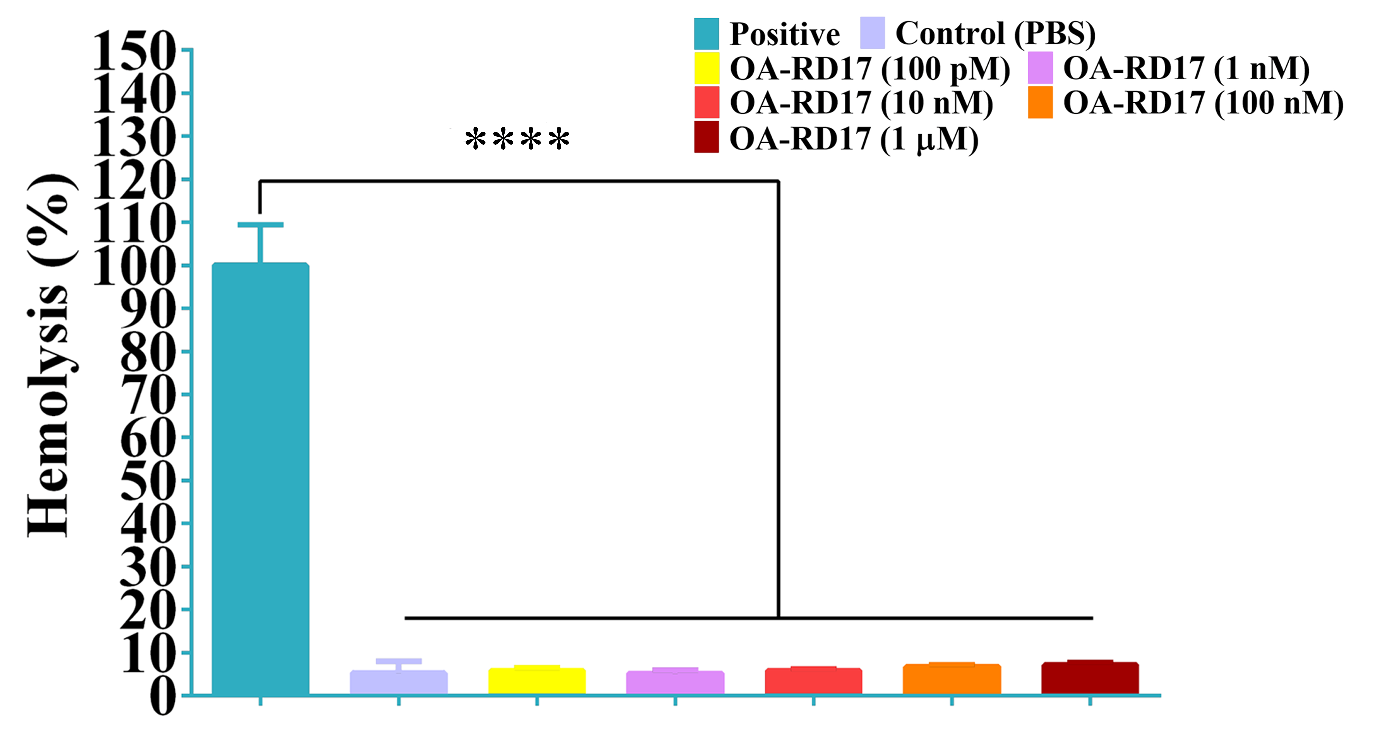


**Figure S2. Hemolytic activity of OA-RD17.**

Hemolytic activity of OA-RD17 (100 pM, 1 nM, 10 nM, 100 nM, and 1 μM) on mouse erythrocytes. All data are expressed as mean ± SEM from three independent experiments performed in quintuplicate, *****P* < 0.0001 indicates statistically significant difference compared to positive control.


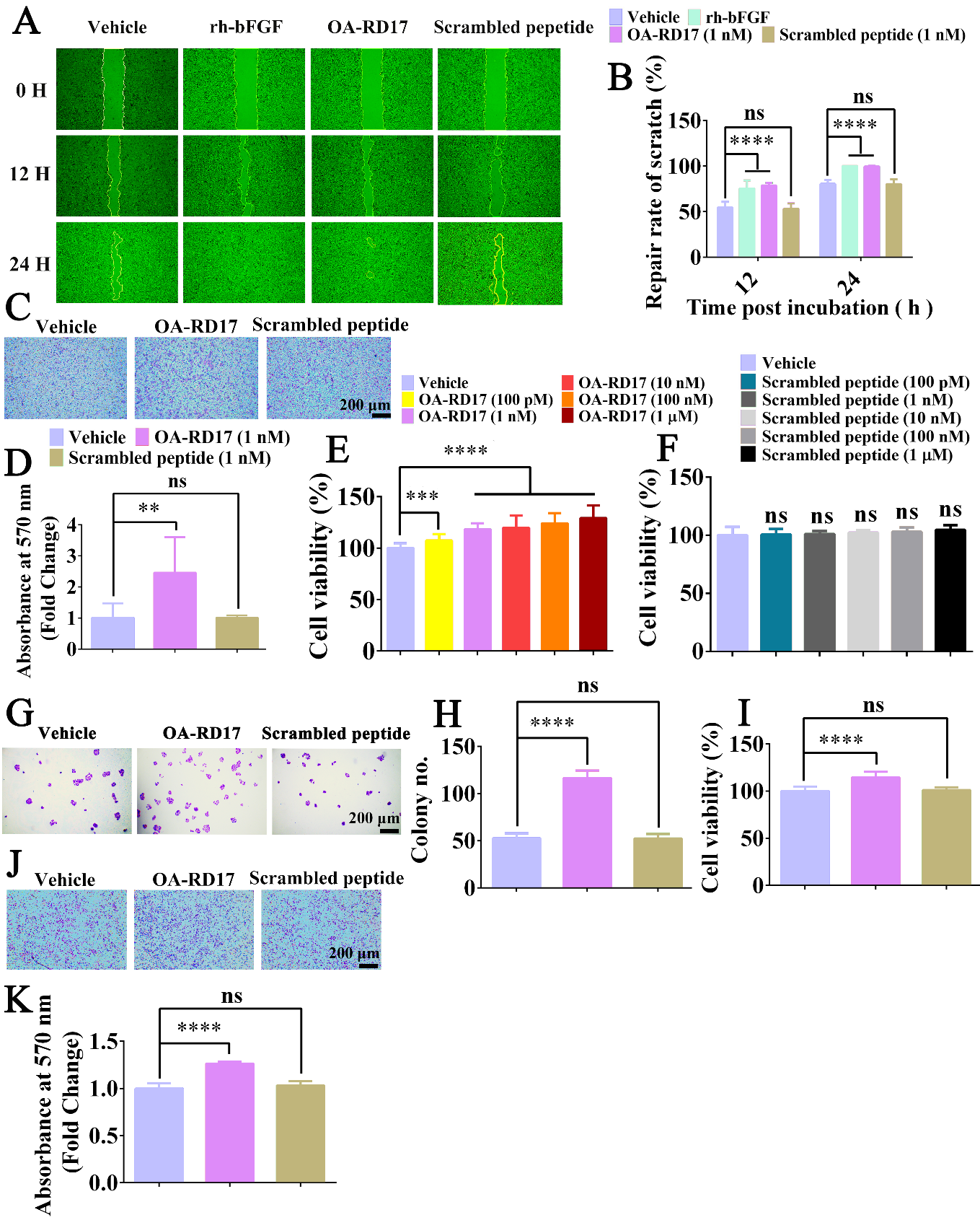


**Figure S3. Pro-healing ability of OA-RD17 at cellular level.**

1. Representative graphs showing ability of OA-RD17 to promote keratinocyte scratch healing at 0, 12, and 24 h.
2. Quantification of keratinocyte scratch repair at 12 and 24 h.

C. Representative image of keratinocyte migration after incubation with OA-RD17 for 24 h, scale bar 200 μm.

D. Quantification of keratinocyte migration after OA-RD17 treatment for 24 h.

E. Promoting effects of OA-RD17 (100 pM, 1 nM, 10 nM, 100 nM, and 1 μM) on keratinocyte proliferation after incubation for 24 h.

F. Promoting effects of scrambled peptide (100 pM, 1 nM, 10 nM, 100 nM, and 1 μM) on keratinocyte proliferation after co-incubation for 24 h.

G. Representative graph of keratinocyte colony formation after two weeks incubation with OA-RD17, scale bar 200 μm.

H. Quantification of keratinocyte colony formation number.

I. Proliferation ability of macrophages after incubation with OA-RD17 for 24 h.

J. Representative image of macrophage migration after 24 h incubation of OA-RD17. Scale bar 200 μm.

K. Quantification of macrophage migration number.

All data are expressed as mean ± SEM from three independent experiments performed in quintuplicate. ns, no significance; ***P* < 0.01, ****P* < 0.001, and *****P* < 0.0001 indicate statistically significant difference compared to vehicle.


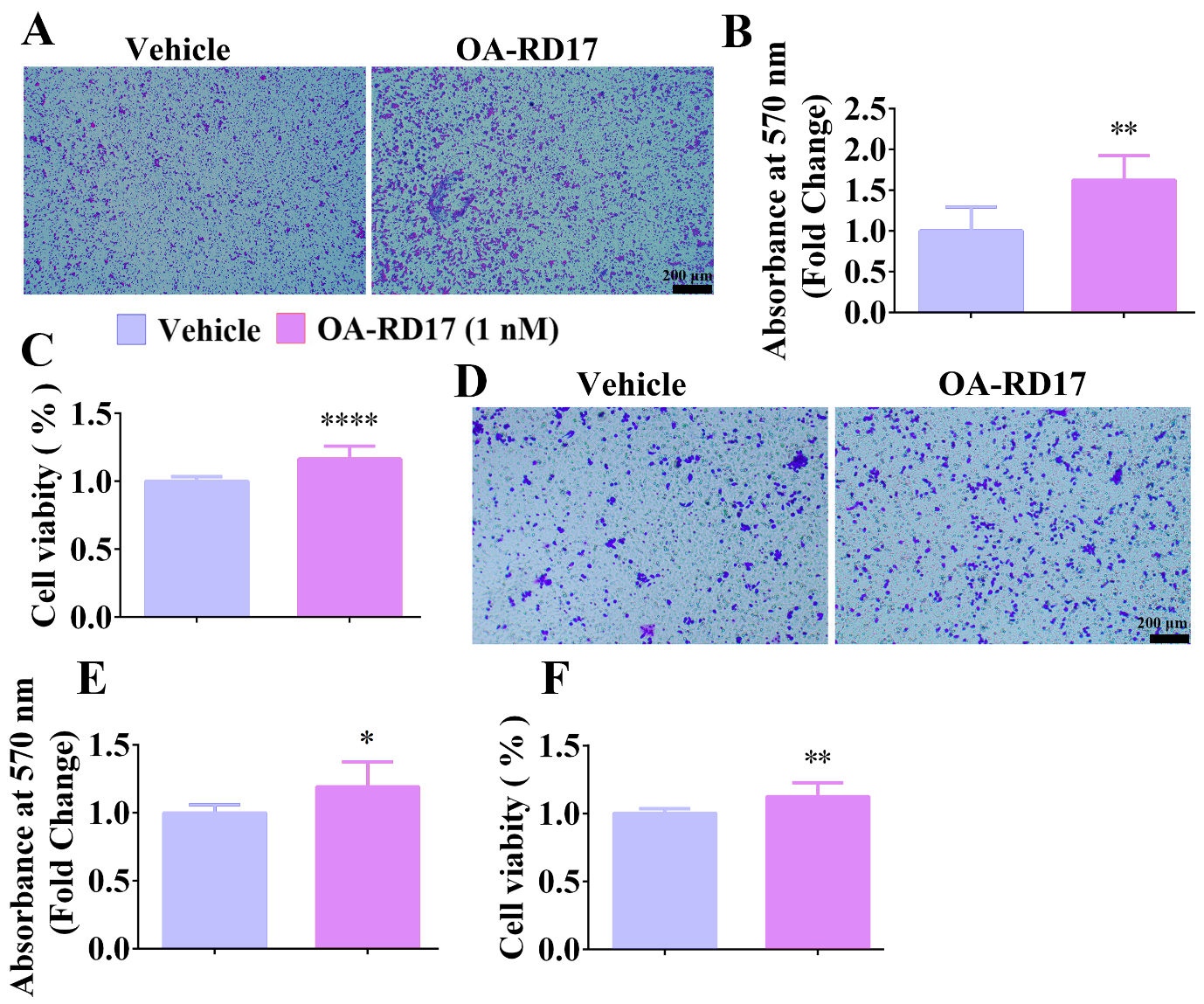


**Figure S4.** **OA-RD17 promoted migration and proliferation of primary mouse keratinocytes and macrophages.**

A. Representative images of migration of primary keratinocytes from Kunming mice after incubation with OA-RD17 for 24 h, scale bar 200 μm.

B. Quantification of primary keratinocyte migration after OA-RD17 treatment for 24 h.

C. Promoting effects of OA-RD17 (1 nM) on primary keratinocytes from Kunming mice proliferation after incubation for 24 h.

D. Representative images of migration of primary macrophages from Kunming mice after incubation with OA-RD17 for 24 h, scale bar 200 μm.

E. Quantification of primary macrophages migration after OA-RD17 treatment for 24 h.

F. Promoting effects of OA-RD17 (1 nM) on primary macrophages from Kunming mice proliferation after incubation for 24 h.

All data are expressed as mean ± SEM from three independent experiments performed in triplicate. **P* < 0.05, ***P* < 0.01, and *****P* < 0.0001 indicate statistically significant difference compared to vehicle.

**
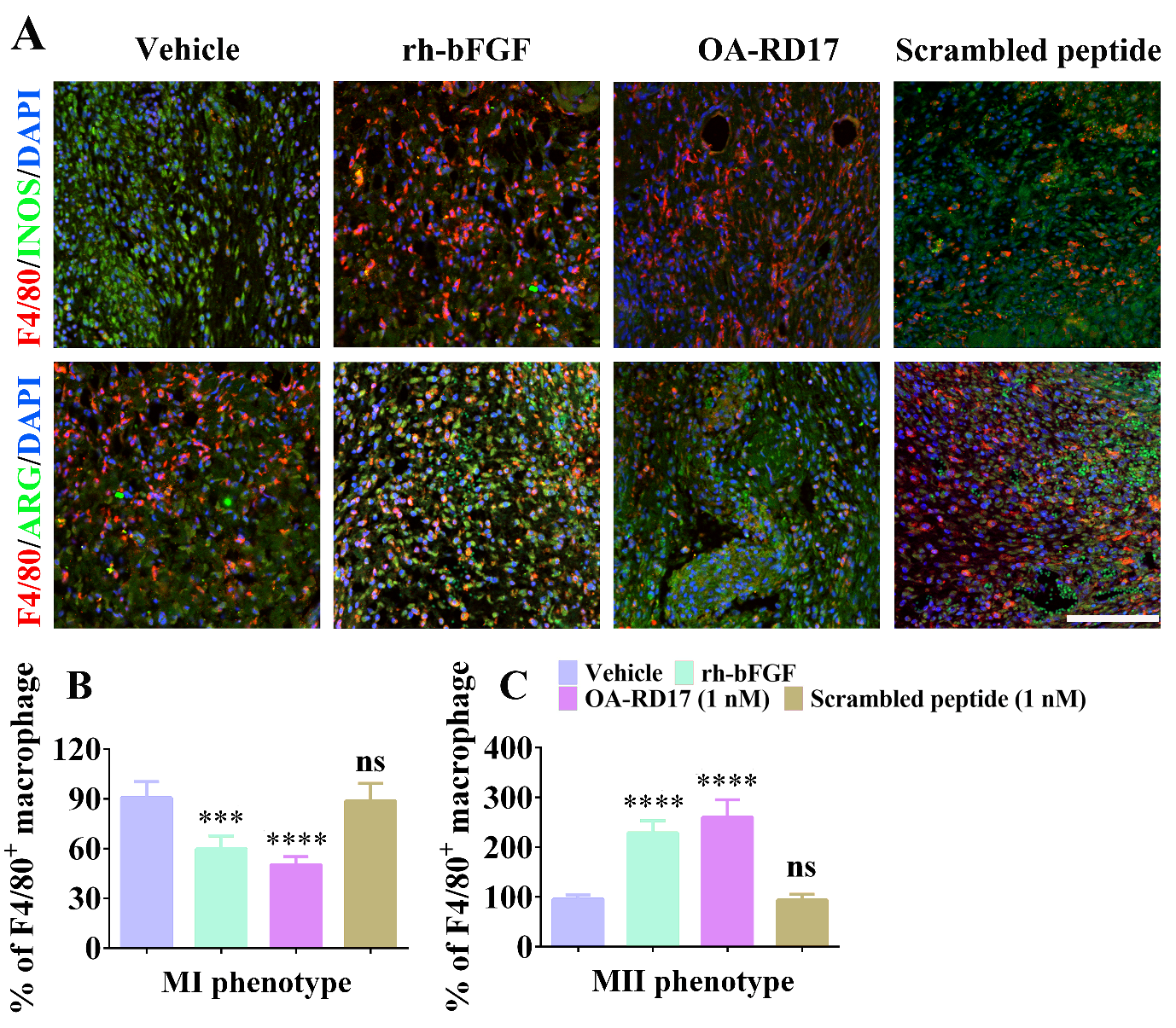
**

**Figure S5.OA-RD17 promoted macrophage polarization from MI to MII phenotype.** Representative images of immunofluorescence staining of MⅠ and MⅡ macrophage phenotypes after 8 days of treatment with PBS, rh-bFGF (100 ng/ml), scrambled peptide (1 nm), or OA-RD17 (1 nm); F4/80 red fluorescence for macrophages, iNOS green fluorescence for MⅠ macrophages, Arg green fluorescence for MⅡ macrophages, and DAPI blue for nuclei; scale bar 50 µm.

1. Quantification of positive-staining intensity of MI phenotype macrophages.
2. Quantification of positive-staining intensity of MⅡ phenotype macrophages.

All data are expressed as mean ± SEM from three independent experiments performed in quintuplicate. ns, no significance; ****P* < 0.001 and *****P* < 0.0001 indicate statistically significant difference compared to vehicle.


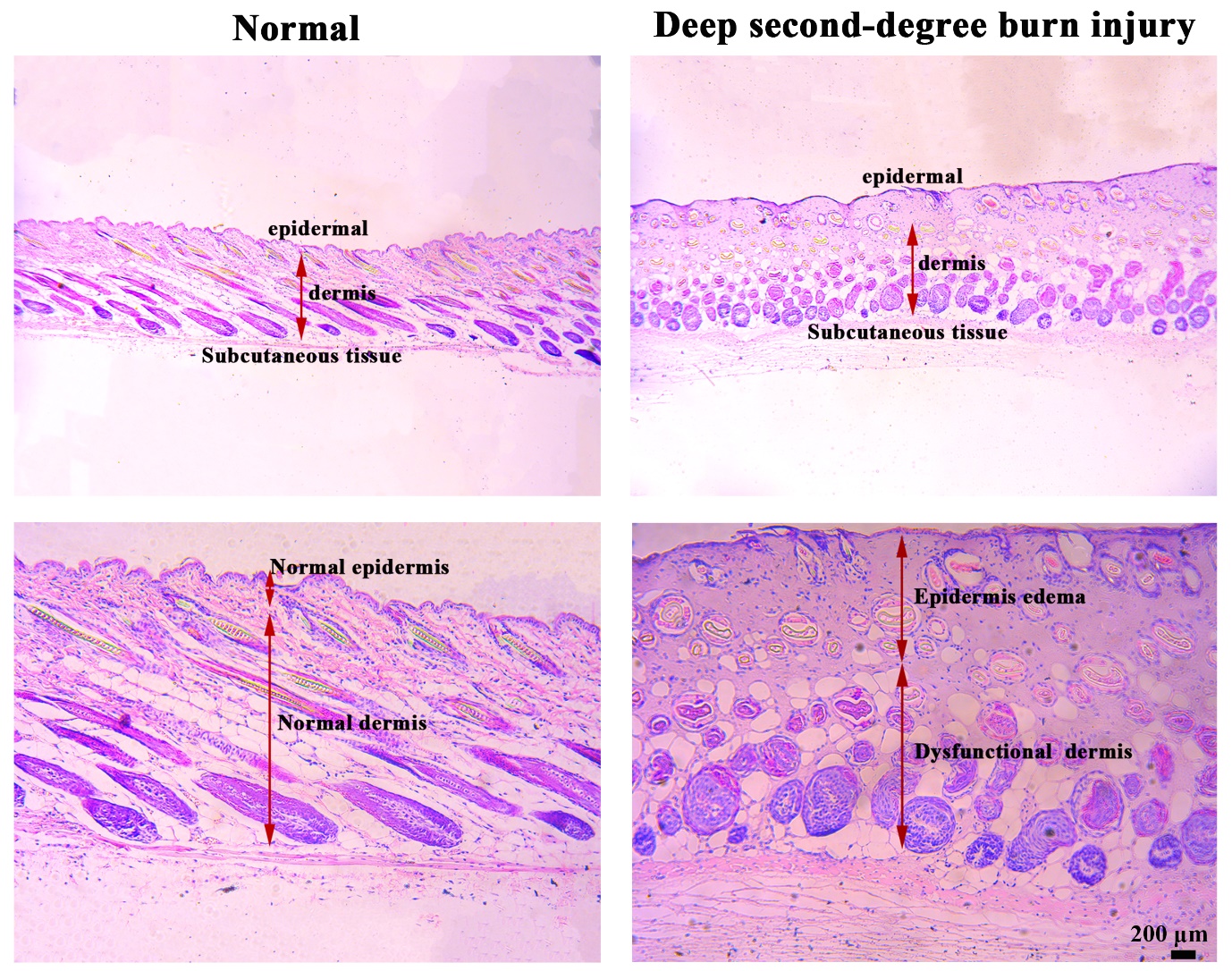


**Figure S6. Hematoxylin and eosin (H&E) staining of deep second-degree burn in mice.** H&E staining indicated pathological changes in normal skin and burned skin (1 h after burn) of mice, scale bar 200 μm. H&E staining of mouse skin burned for 1 h was obtained from every mouse burn model.


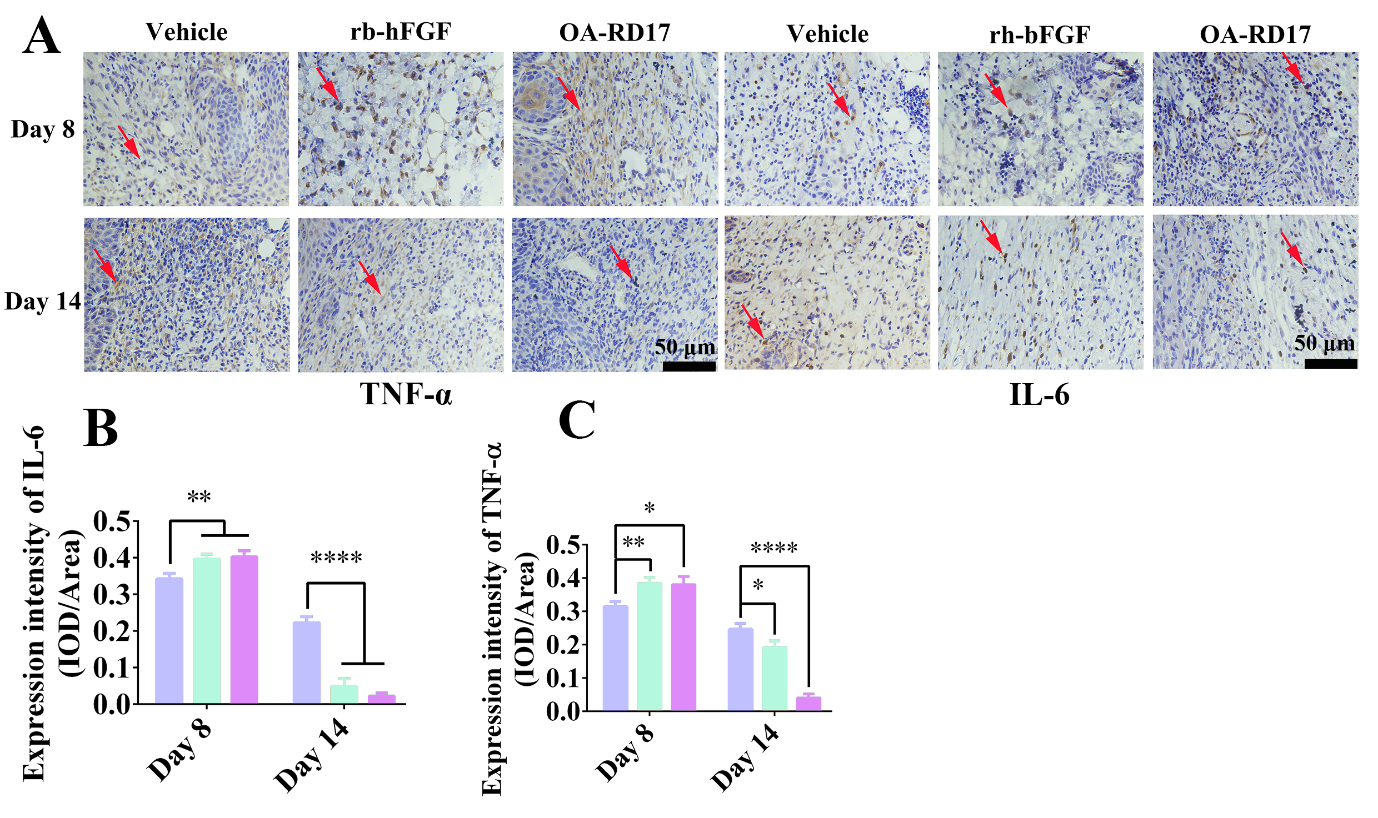


**Figure S7.** **OA-RD17 significantly suppressed the expression of IL-6 and TNF-α**

A. Representative images of immunohistochemical staining of inflammatory factors IL-6 and TNF-α in wound area on days 8 and 14 after treatment with PBS, rh-bFGF (100 ng/mL), or OA-RD17 (1 nM). Red arrows indicate positive staining, scale bar 50 μm.

B-C. Quantification of IL-6 and TNF-α expression in wound area; positive expression is defined as intensity of positive staining per unit area.

All data are expressed as mean ± SEM from three independent experiments performed in quintuplicate. **P* < 0.05, ***P* < 0.01 and *****P* < 0.0001 indicate statistically significant difference compared to vehicle.

**
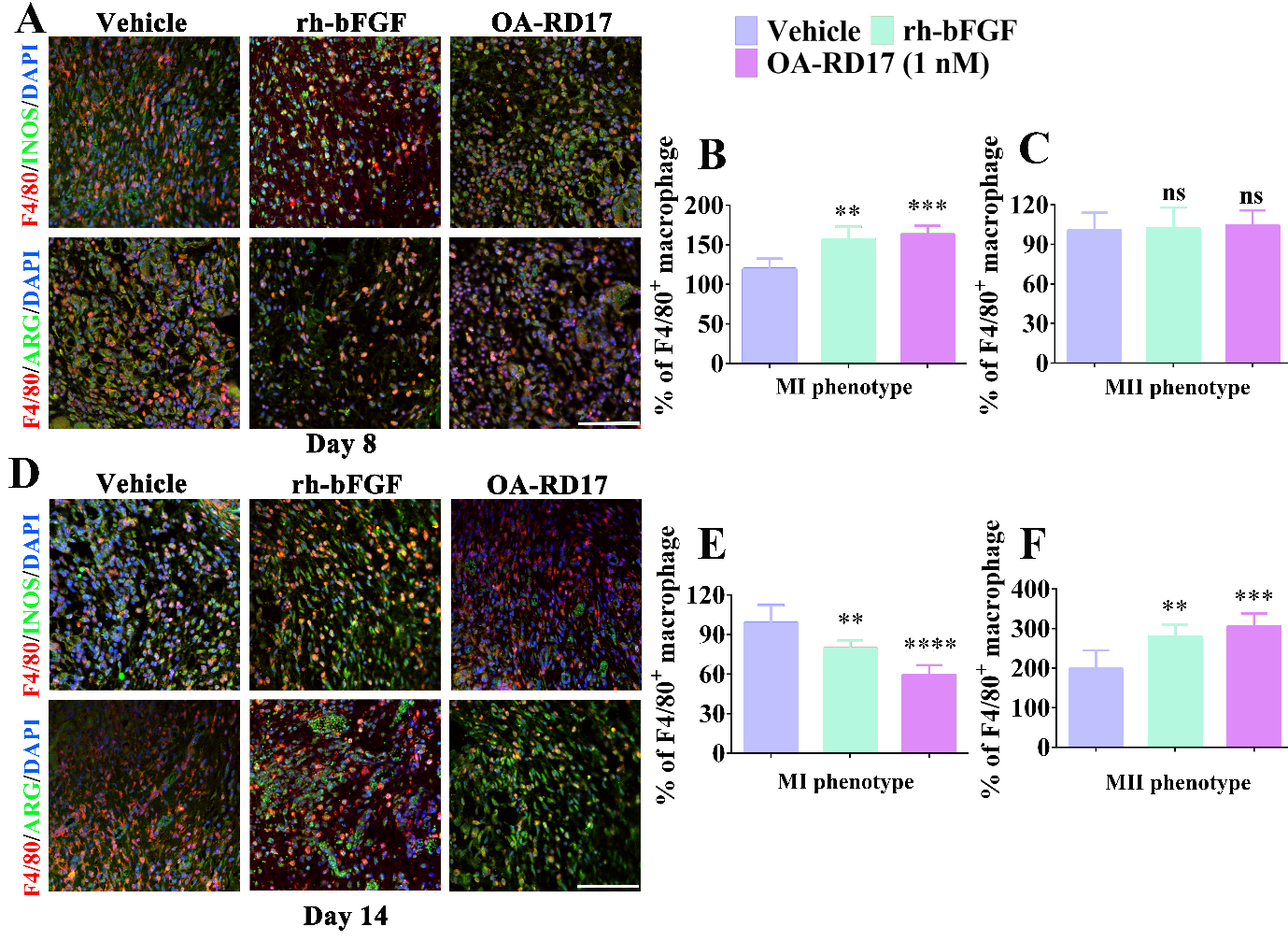
**

**Figure S8. Polarization of macrophages in wound area of mice with deep second-degree burns on days 8 and 14.**

1. Representative graphs of immunofluorescence staining of MⅠ and MⅡ macrophage expression in mice treated with PBS, rh-bFGF (100 ng/mL), or OA-RD17 (1 nM) on day 8; F4/80 red fluorescence for macrophages, INOS green fluorescence for MⅠ macrophages, ARG green fluorescence for MⅡ macrophages, and DAPI blue for nuclei; scale bar 50 µm.
2. Quantification of positive-staining intensity of MⅠ phenotype macrophages on day 8 of treatment with PBS, rh-bFGF, and OA-RD17.
3. Quantification of positive-staining intensity of MⅡ phenotype macrophages on day 8 of treatment with PBS, rh-bFGF, and OA-RD17.
4. Representative graphs of immunofluorescence staining of MⅠ and MⅡ macrophage expression in mice treated with PBS, rh-bFGF (100 ng/mL), or OA-RD17 (1 nM) on day 14; F4/80 red fluorescence for macrophages, INOS green fluorescence for MⅠ macrophages, ARG green fluorescence for MⅡ macrophages, and DAPI blue for nuclei; scale bar 50 µm.
5. Quantification of positive-staining intensity of MⅠ phenotype macrophages on day 14 of treatment with PBS, rh-bFGF, and OA-RD17.
6. Quantification of positive-staining intensity of MⅡ phenotype macrophages on day 14 of treatment with PBS, rh-bFGF, and OA-RD17.

All data are expressed as mean ± SEM from three independent experiments performed in quintuplicate, ns, no significance, ***P* < 0.01, ****P* < 0.001, and *****P* < 0.0001 indicate statistically significant difference compared to vehicle.


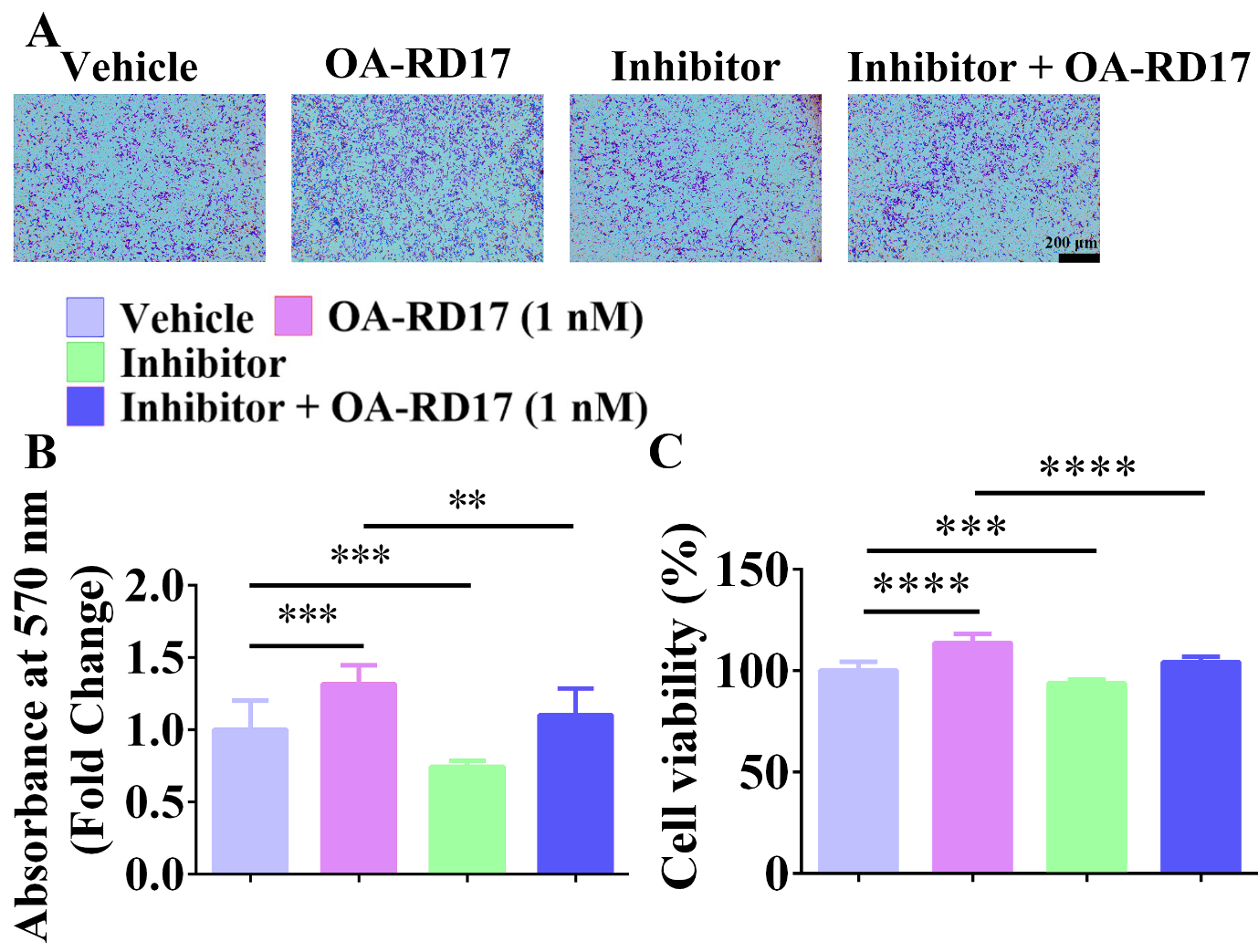


**Figure S9. MAPK signaling pathway inhibitors significantly inhibited proliferation- and migration-promoting activity of OA-RD17 on macrophages.** A. Transwell plots of effect of OA-RD17 on macrophage migration following application of MAPK signaling pathway inhibitors, scale bar 200 μm.

B. Quantification of macrophage migration number.

C. Changes in pro-proliferative activity of OA-RD17 on mouse macrophages following application of MAPK signaling pathway inhibitors.

All data are expressed as mean ± SEM from three independent experiments performed in quintuplicate, ***P* < 0.01, ****P* < 0.001, and *****P* < 0.0001.

**
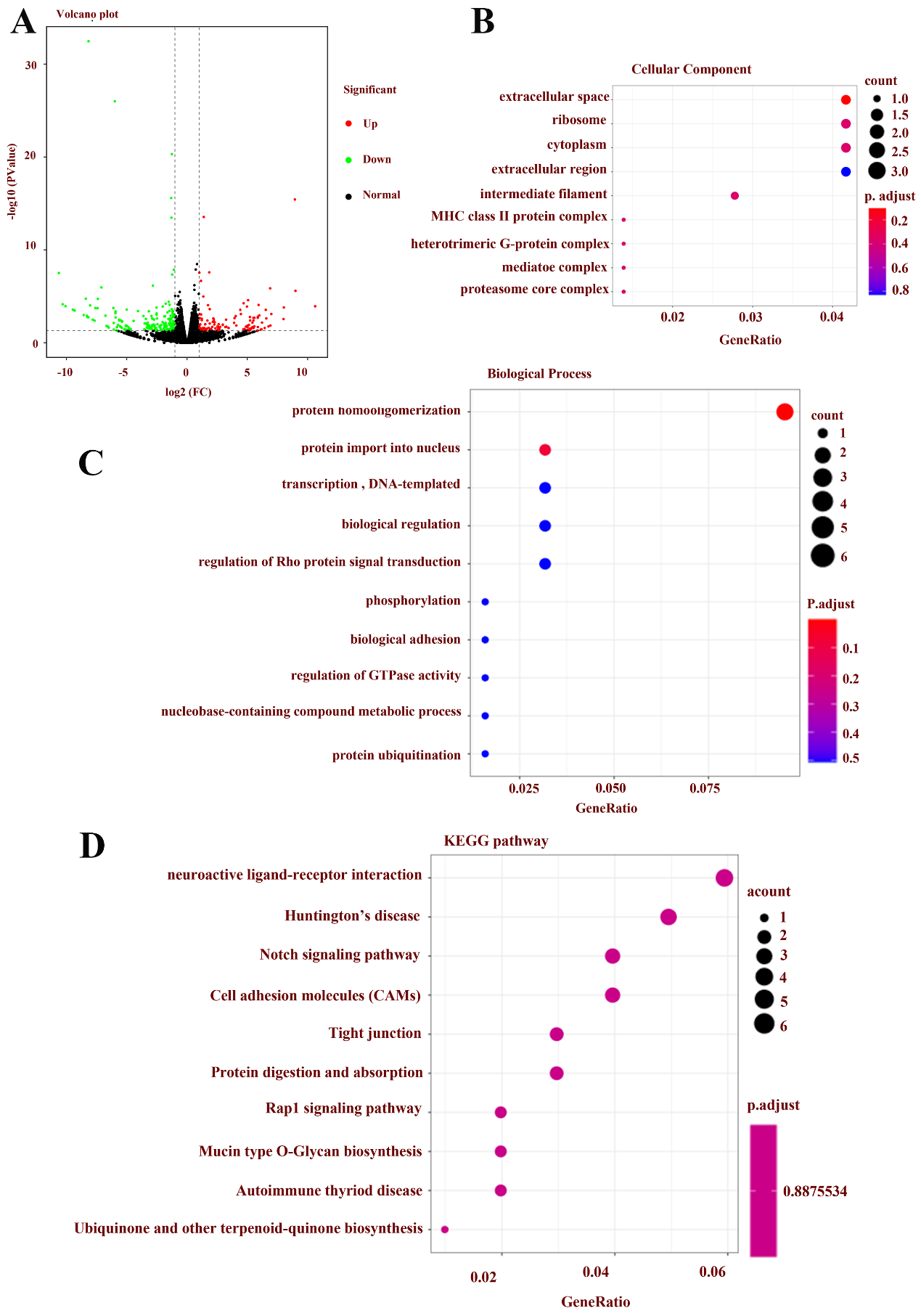
**

**Figure S10. RNA sequencing of mRNA levels of differentially expressed genes (DEGs) involved in** **biological processes, components, and signaling pathways following OA-RD17 treatment in keratinocytes.**

A. Volcano map of differentially expressed mRNAs following OA-RD17 treatment of keratinocytes, with 126 DEGs up-regulated and 195 DEGs down-regulated.

B. Differentially expressed mRNAs involved in biological components.

C. Differentially expressed mRNAs involved in biological processes.

D. KEGG enrichment analysis of signaling pathways in differentially expressed mRNAs regulated by OA-RD17. The ubiquinone and other terpenoid quinone biosynthesis is non-human pathway.


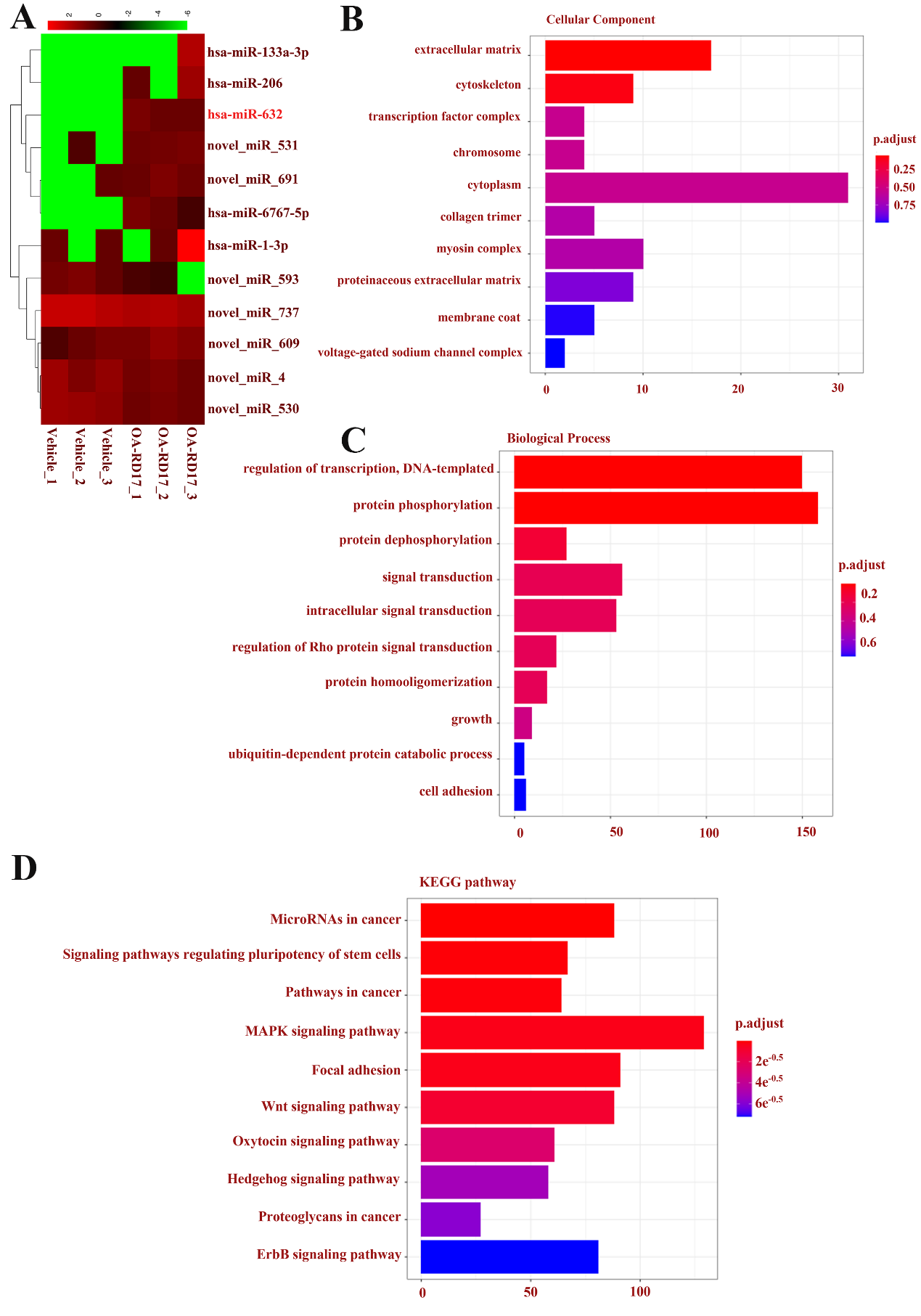


**Figure S11. RNA sequencing of differentially expressed miRNAs involved in biological components, processes, and signaling pathways following OA-RD17 treatment in keratinocytes.**

A. OA-RD17 treatment of keratinocytes up-regulated eight differentially expressed miRNAs and down-regulated four differentially expressed miRNAs.

B. Differentially expressed miRNAs involved in biological components.

C. Differentially expressed miRNAs involved in biological processes.

D. KEGG enrichment analysis of signaling pathways in differentially expressed miRNAs.


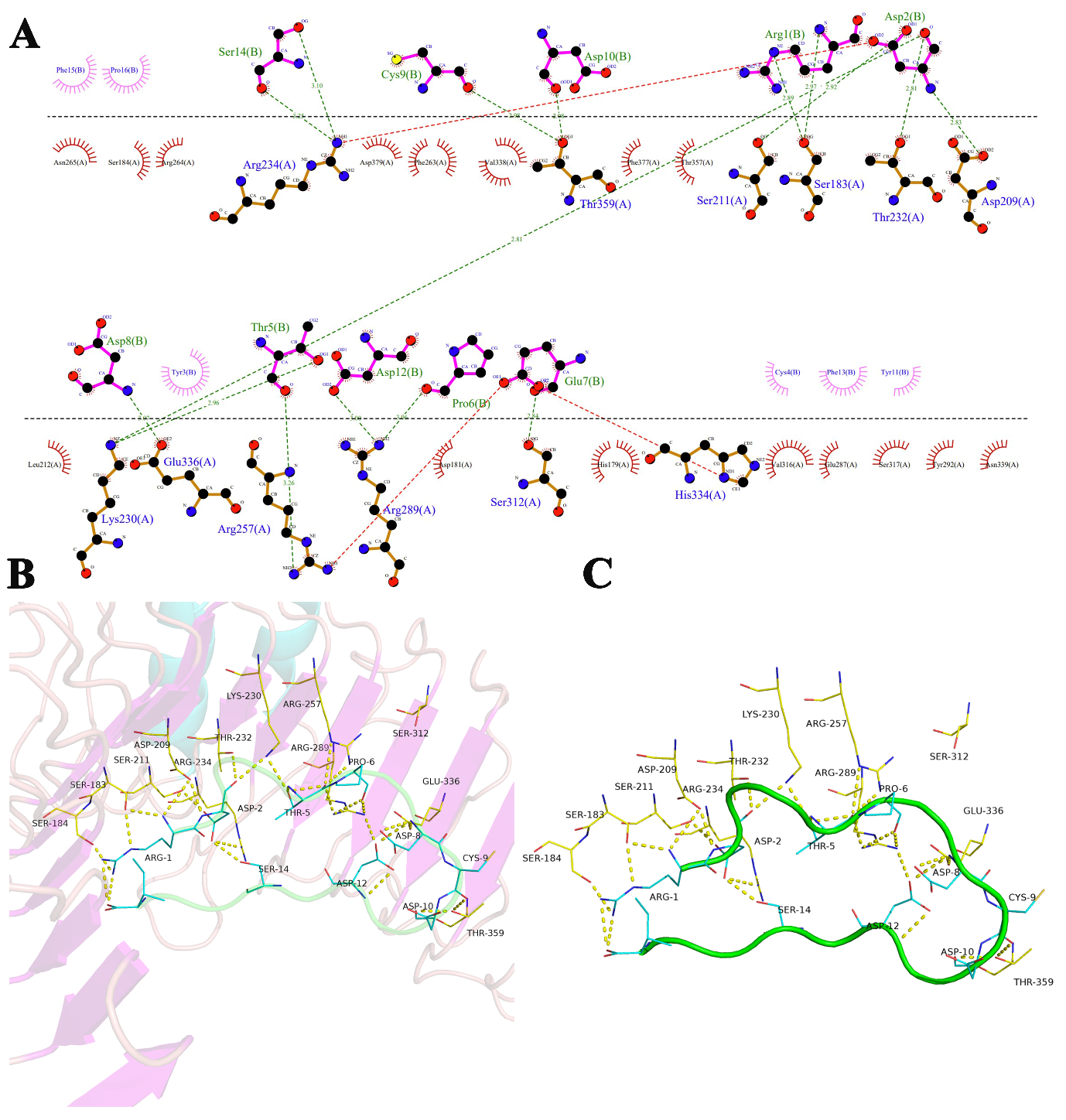


**Figure S12. Molecular docking of TLR4 and OA-RD17.**

A. TLR4 and OA-RD17 showed multiple interacting residues and formed multiple hydrogen bonds to promote stable binding of TLR4 to OA-RD17. A represents TLR4, B represents OA-RD17.

B. Multiple amino acid residues of OA-RD17 bonded to hydrophobic region of TLR4 via hydrogen bonding and electrostatic interactions.

C. Amino acid residues at different sites of OA-RD17 bonded to TLR4 amino acid residues via hydrogen bonding and electrostatic interactions.


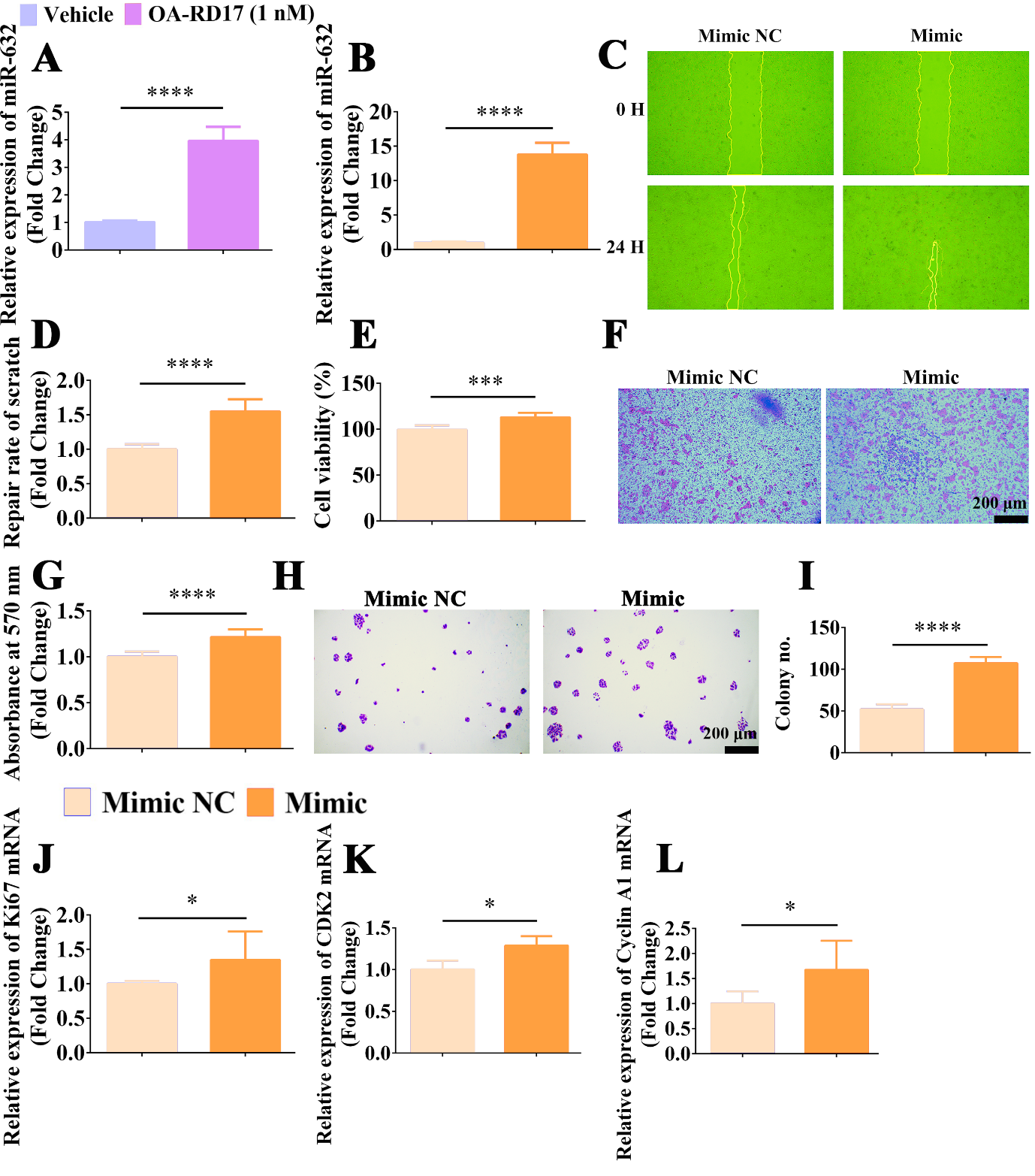


**Figure S13. OA-RD17 significantly up-regulated miR-632, which significantly promoted keratinocyte proliferation and migration.**

A-B. RT-qPCR detected the expression of miR-632 in keratinocytes treated with OA-RD17 and miR-632 mimic for 24 h.

C-D. Representative graph of keratinocyte scratch repair after up-regulation of miR-632 expression and quantification of keratinocyte scratch repair rate.

E. Changes in proliferation ability of keratinocytes following up-regulation of miR-632 expression.

F-G. Representative plots of migrating keratinocytes at 24 h after up-regulation of miR-632 expression, determined by Transwell assay, scale bar 200 μm; quantification of number of migrating keratinocytes.

H-I. Representative graph of cell colony formation after two weeks of up-regulation of miR-632 expression in keratinocytes, scale bar 200 μm; quantification of colony formation in keratinocytes.

J-L. Expression of Ki67, CDK2, and Cyclin A1 in keratinocytes after 24 h of up-regulation of miR-632, detected by RT-qPCR.

All data are expressed as mean ± SEM from three independent experiments performed in quintuplicate, **P <* 0.05, ****P <* 0.001, and *****P <* 0.0001 indicate statistically significant difference compared to NC mimic.


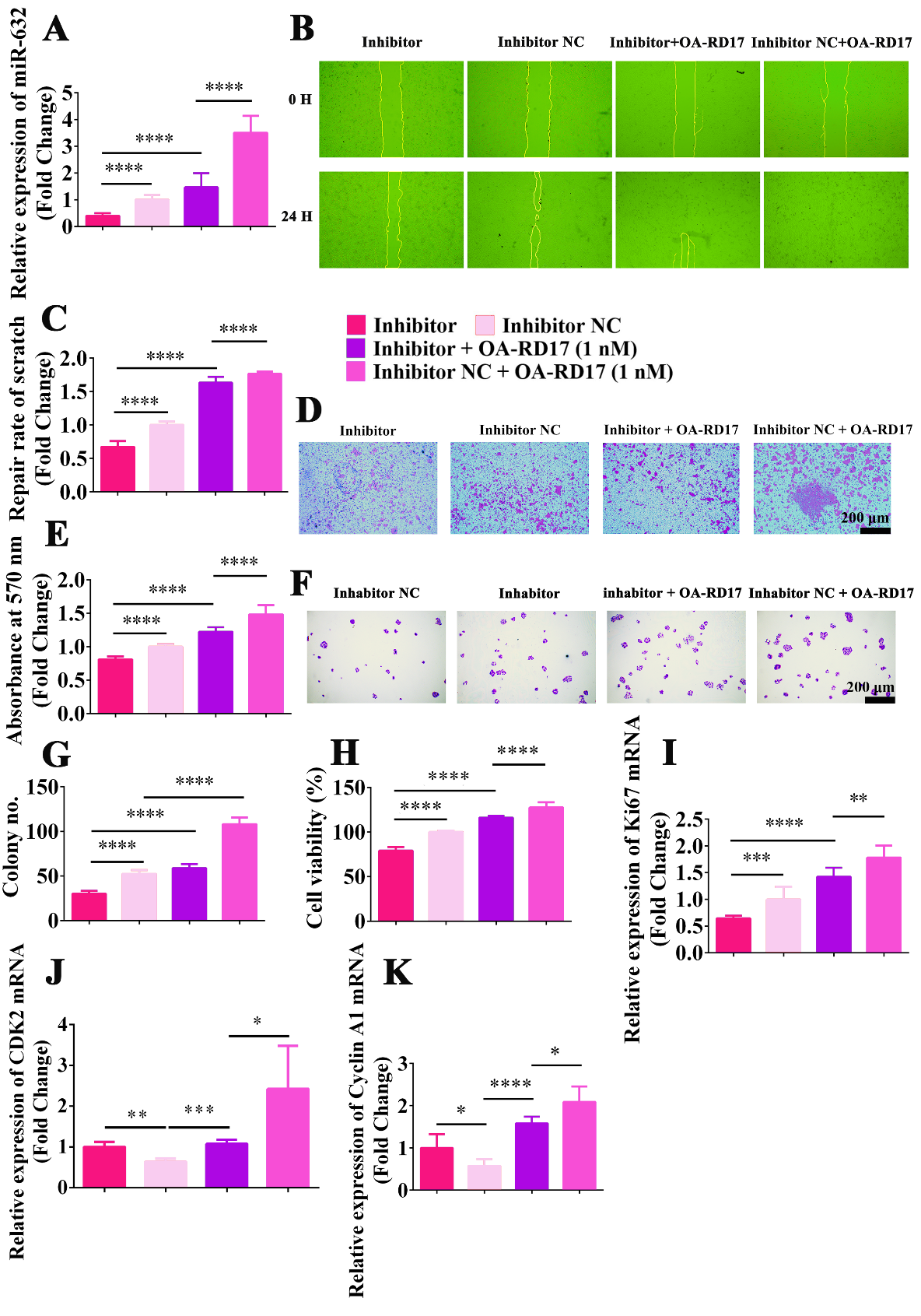


**Figure S14. Inhibition of miR-632 expression significantly inhibited keratinocyte proliferation and migration, while OA-RD17 restored effect of miR-632 down-regulation on keratinocyte proliferation and migration.**

A. Changes in miR-632 expression after miR-632 inhibitor treatment of keratinocytes for 24 h and in miR-632 expression after miR-632 inhibitor co-treatment with OA-RD17, detected via RT-qPCR.

B-C. Representative images of keratinocyte scratch repair and quantification of scratch repair after miR-632 inhibitor, NC, and miR-632 inhibitor co-treatment with OA-RD17 for 24 h.

D-E. Representative Transwell graphs of keratinocyte migration, scale bar 200 μm, and quantification of keratinocyte migration number after 24 h of miR-632 inhibitor, NC, and miR-632 inhibitor co-treatment with OA-RD17.

F-G. Representative images of keratinocyte colony formation after miR-632 inhibitor, NC, and miR-632 inhibitor co-treatment with OA-RD17, scale bar 200 μm; quantification of keratinocyte colony formation number.

H. Changes in proliferation activity of keratinocytes after miR-632 inhibitor, NC, and miR-632 inhibitor co-treatment with OA-RD17 for 24 h.

I-K. Expression levels of Ki67, CDK2, and Cyclin A1 in keratinocytes treated with miR-632 inhibitor, NC, and miR-632 inhibitor co-treated with OA-RD17 for 24 h, determined by RT-qPCR.

All data are expressed as mean ± SEM from three independent experiments performed in quintuplicate, **P* < 0.05, ***P* < 0.01, ****P* < 0.001, and *****P* < 0.0001.


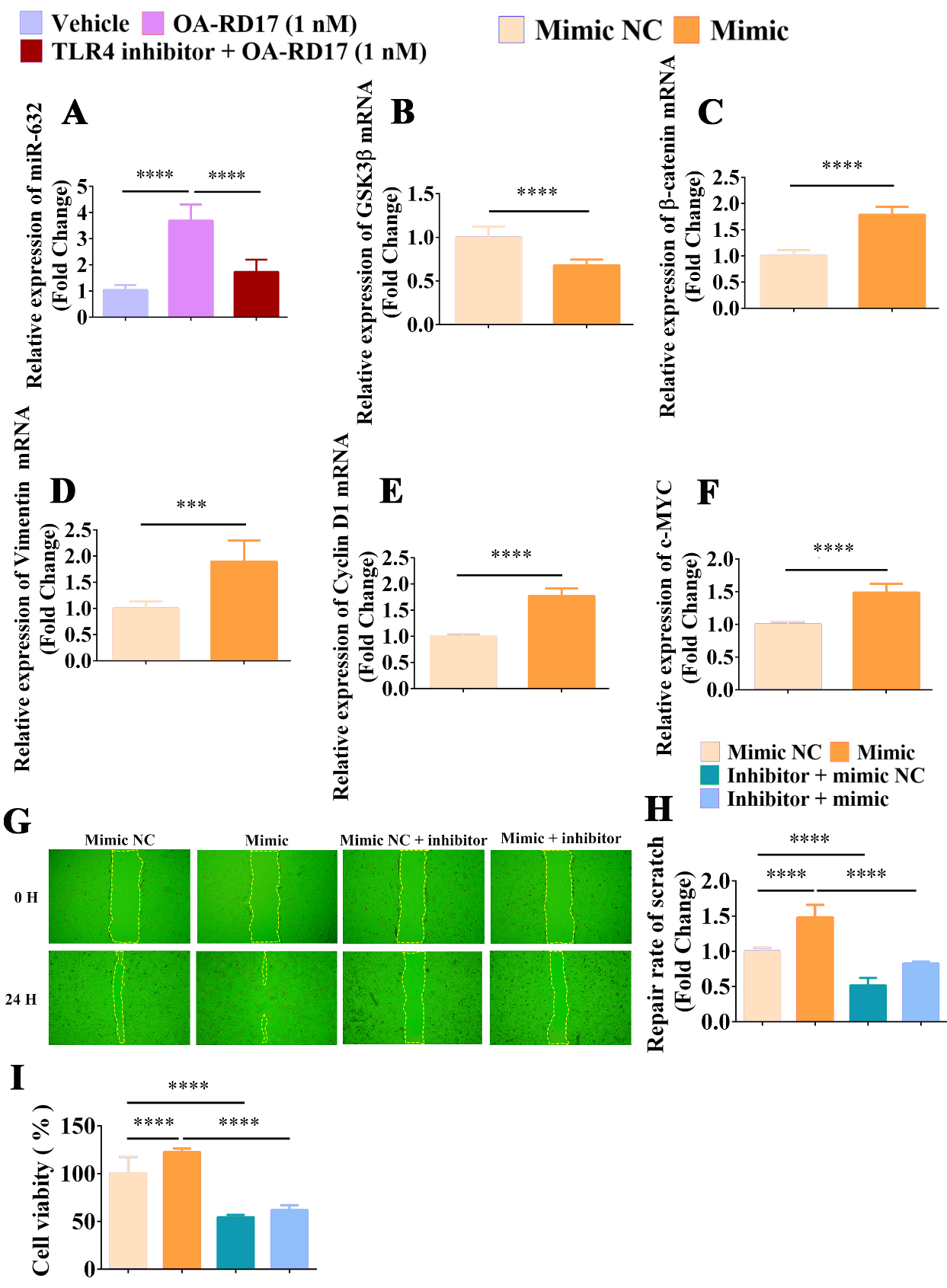


**Figure S15. Up-regulation of miR-632 expression significantly promoted Wnt/β-catenin signaling pathway activation.**

A. After treatment with TLR4 specific inhibitor, the expression levels of miR-632 in OA-RD17-treated (for 24 h) keratinocytes were measured by RT- qPCR.

B-F. GSK3β, β-catenin, c-MYC, Cyclin D1, and Vimentin mRNA expression after up-regulation of miR-632 expression, detected by RT-qPCR.

G-H. Representative images of miR-632 pro-keratinocyte scratch repair after β-catenin inhibitor application, and quantification of keratinocyte scratch repair.

I. Changes in pro-keratinocyte proliferative activity of miR-632 after application of β-catenin inhibitors.

All data are expressed as mean ± SEM from three independent experiments performed in quintuplicate, ****P* < 0.001 and *****P* < 0.0001.

**
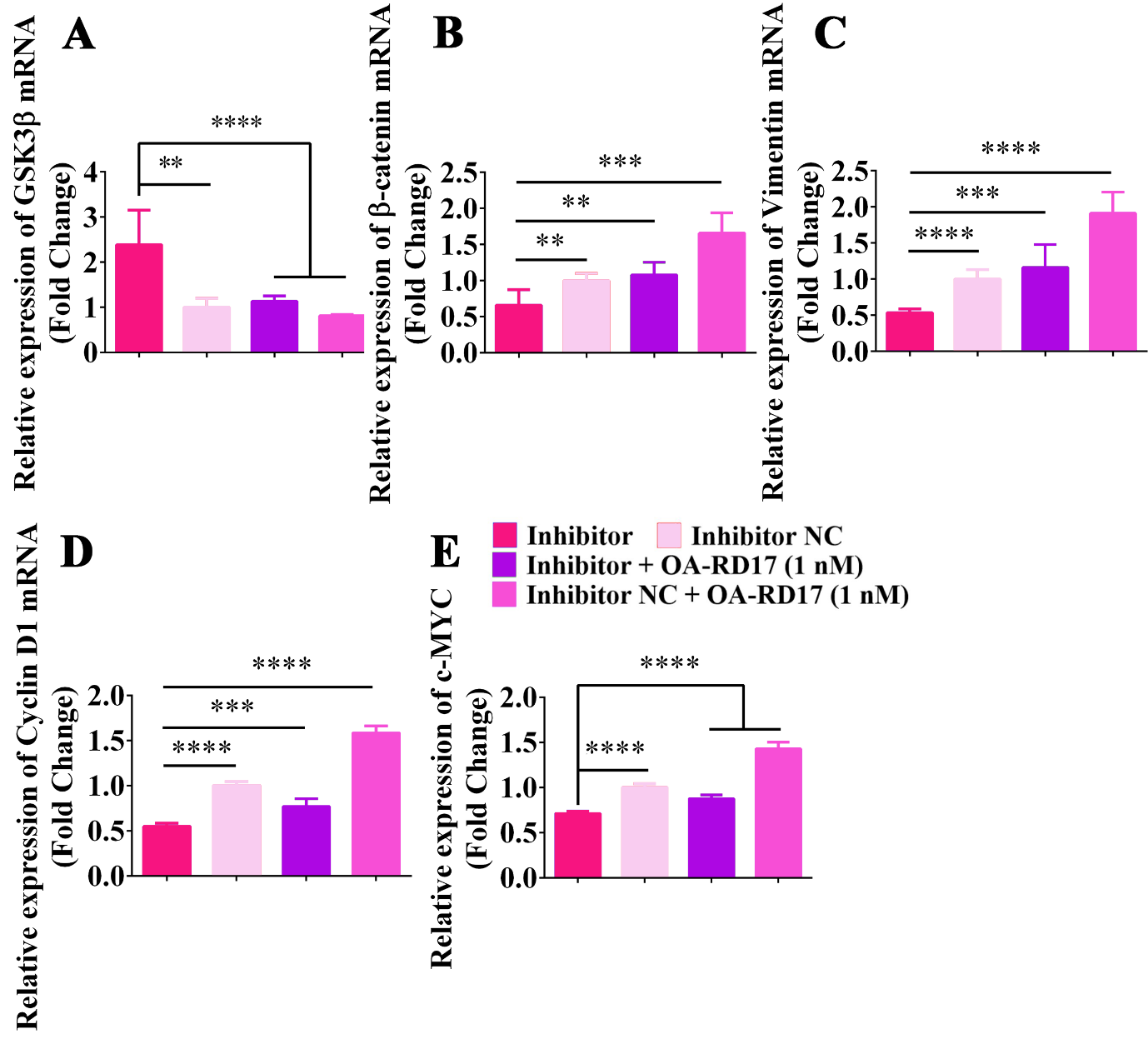
**

**Figure S16. Expression of GSK3β, β-catenin, c-MYC, Cyclin D1, and Vimentin mRNA after down-regulation of miR-632.**

A-E. Expression of GSK3β, β-catenin, c-MYC, Cyclin D1, and Vimentin mRNA were detected by RT-qPCR after down-regulation of miR-632 expression.

All data are expressed as mean ± SEM from three independent experiments performed in quintuplicate, ***P* < 0.01, ****P* < 0.001, and *****P* < 0.0001.

**
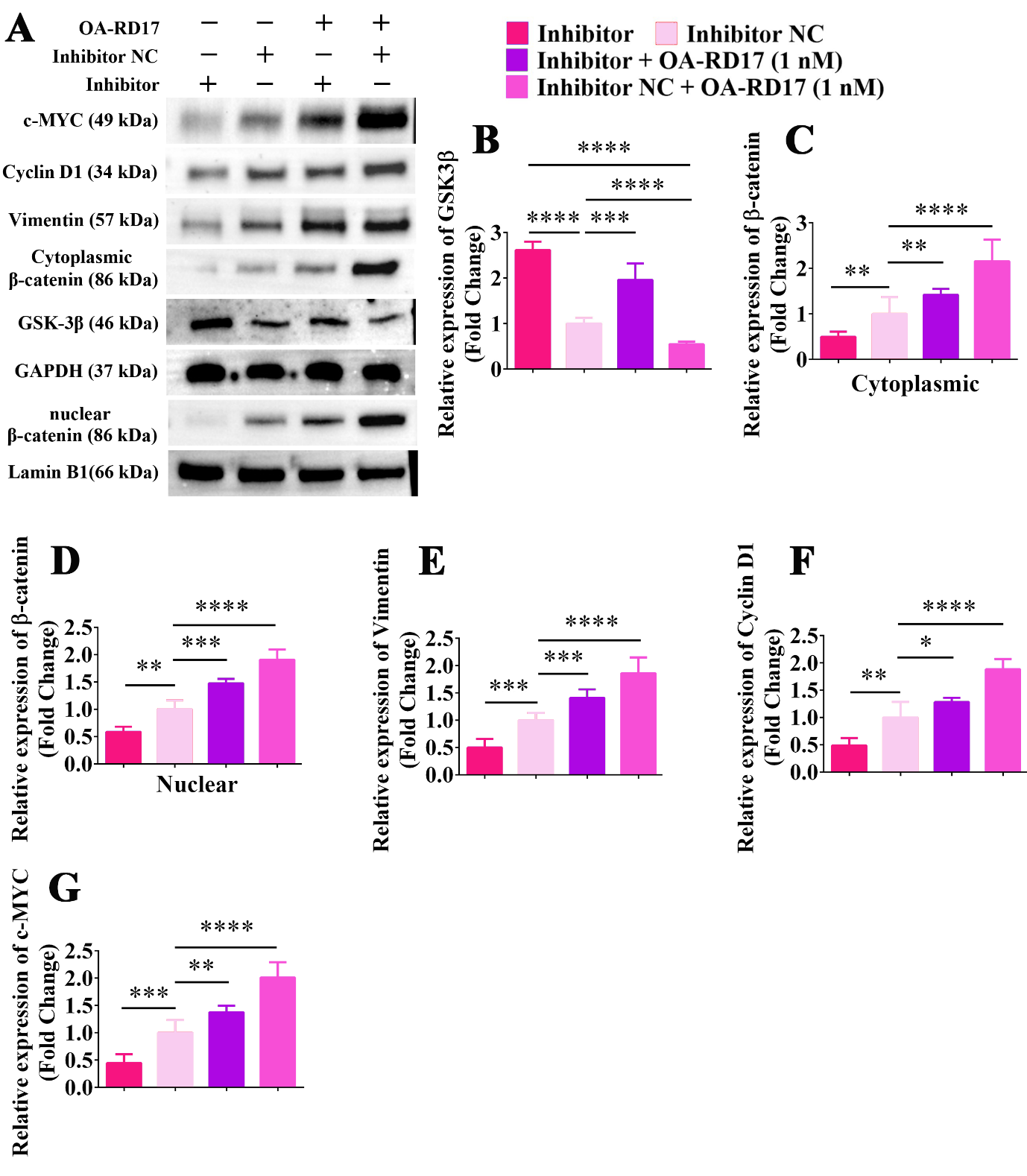
**

**Figure S17. Down-regulation of miR-632 significantly inhibited Wnt/β-catenin signaling pathway activation.**

A. Effects on Wnt/β-catenin signaling pathway after down-regulation of miR-632, detected by western blotting.

B-G. Quantification of GSK3β, β-catenin, Vimentin, Cyclin D1, and c-MYC expression after down-regulation of miR-632.

All data are expressed as mean ± SEM from three independent experiments, **P* < 0.05, ***P* < 0.01, ****P* < 0.001, and *****P* < 0.0001.


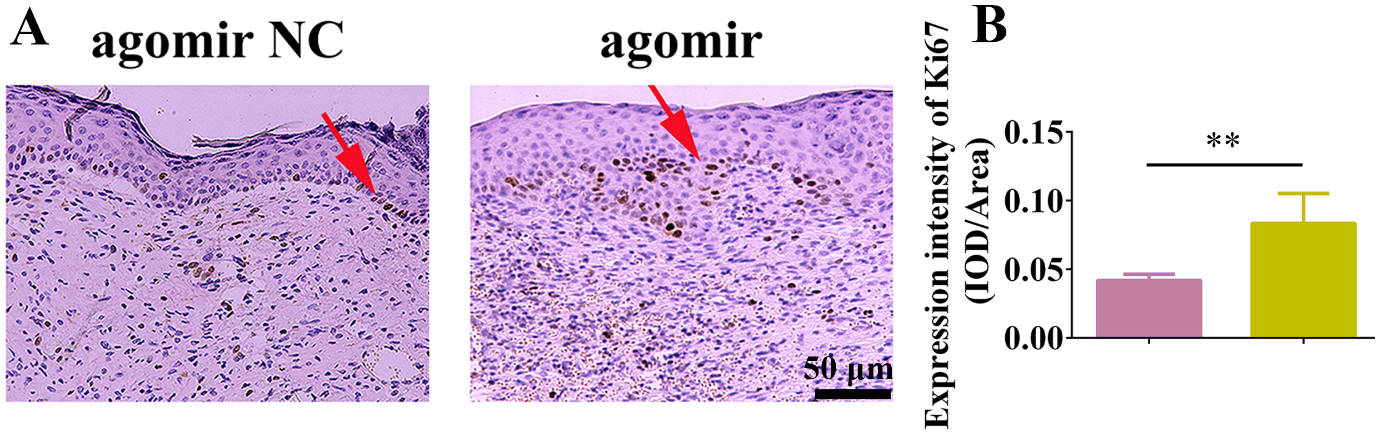


**Figure S18. MiR-632 significantly promoted the expression of Ki67 in epidermis of wound area.**

A. Representative images of immunohistochemical staining of Ki67 expression in epidermis of wound area.

B. Quantification the expression of Ki67 in epidermis of wound area.

All data are expressed as mean ± SEM from three independent experiments, ***P <* 0.01.
